# PLK1 promotes the mitotic surveillance pathway by controlling cytosolic 53BP1 availability

**DOI:** 10.15252/embr.202357234

**Published:** 2023-10-27

**Authors:** Matteo Burigotto, Vincenza Vigorito, Colin Gliech, Alessia Mattivi, Sabrina Ghetti, Alessandra Bisio, Graziano Lolli, Andrew J Holland, Luca L Fava

**Affiliations:** ^1^ Armenise‐Harvard Laboratory of Cell Division, Department of Cellular, Computational and Integrative Biology – CIBIO University of Trento Trento Italy; ^2^ Department of Molecular Biology and Genetics Johns Hopkins University School of Medicine Baltimore MD USA; ^3^ Laboratory of Radiobiology, Department of Cellular, Computational and Integrative Biology – CIBIO University of Trento Trento Italy; ^4^ Laboratory of Protein Crystallography and Structure‐Based Drug Design, Department of Cellular, Computational and Integrative Biology – CIBIO University of Trento Trento Italy; ^5^ Oncology Research Amgen Research Thousand Oaks CA USA; ^6^ Present address: Comprehensive Cancer Centre, School of Cancer and Pharmaceutical Sciences King's College London UK; ^7^ Present address: Organelle Dynamics Laboratory The Francis Crick Institute London UK; ^8^ Present address: Department of Mechanistic Cell Biology Max Planck Institute of Molecular Physiology Dortmund Germany

**Keywords:** 53BP1, kinetochore, mitotic stopwatch pathway, mitotic surveillance pathway, PLK1, Cell Cycle, Post-translational Modifications & Proteolysis, Signal Transduction

## Abstract

53BP1 acts at the crossroads between DNA repair and p53‐mediated stress response. With its interactors p53 and USP28, it is part of the mitotic surveillance (or mitotic stopwatch) pathway (MSP), a sensor that monitors the duration of cell division, promoting p53‐dependent cell cycle arrest when a critical time threshold is surpassed. Here, we show that Polo‐like kinase 1 (PLK1) activity is essential for the time‐dependent release of 53BP1 from kinetochores. PLK1 inhibition, which leads to 53BP1 persistence at kinetochores, prevents cytosolic 53BP1 association with p53 and results in a blunted MSP. Strikingly, the identification of CENP‐F as the kinetochore docking partner of 53BP1 enabled us to show that measurement of mitotic timing by the MSP does not take place at kinetochores, as perturbing CENP‐F‐53BP1 binding had no measurable impact on the MSP. Taken together, we propose that PLK1 supports the MSP by generating a cytosolic pool of 53BP1 and that an unknown cytosolic mechanism enables the measurement of mitotic duration.

## Graphical abstract

Following an abnormally extended mitosis, cells activate p53 via 53BP1. Surprisingly, 53BP1 kinetochore localization is dispensable for this process, whereas PLK1 activity, a negative regulator of 53BP1 localization, appears to be essential.

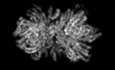

## Introduction

Development and homeostasis of multicellular organisms critically depend on the fidelity of cell division, granting genome stability over a multitude of subsequent mitoses. Mitosis is a highly coordinated set of events involving the abrupt functional and morphological reorganization of the cell in a short time, ultimately leading to the generation of two genetically identical cells. Mitotic duration is determined by the lag between Cyclin B/CDK1 activation, kick‐starting mitotic entry, and the complete activation of an E3 ubiquitin ligase called anaphase‐promoting complex/cyclosome (APC/C), which in turn promotes mitotic exit. Staggered waves of Cyclin B/CDK1 and APC/C activity constitute the core cell cycle clock, driving the alternance between interphase and mitosis (Murray & Kirschner, 1989; Pines, 2011). However, the timing of cell division also depends on an additional rheostat: the spindle assembly checkpoint (SAC). The SAC acts by delaying APC/C activation until all chromosomes are bi‐oriented on the mitotic spindle, thereby lowering the frequency of chromosome segregation errors (Collin *et al*, 2013).

Due to the high metabolic demand of the mitotic status and the inability of the SAC to completely hinder Cyclin B degradation, an arrest in mitosis cannot be sustained indefinitely (Brito & Rieder, 2006; Doménech *et al*, 2015). Moreover, merotelic kinetochore‐microtubule attachments cannot always be corrected thanks to the activity of the SAC (Gregan *et al*, 2011; Dudka *et al*, 2018). Thus, damaged or stressed cells often undergo an extension of the mitotic duration that is mediated by the SAC, yet this might not suffice to grant complete fidelity. Seminal work by Uetake and Sluder (2010) revealed the presence of an additional fail‐safe mechanism by demonstrating that mitoses whose duration exceeds a critical threshold (90 min in their experimental conditions) yield a progeny that become arrested in the subsequent G_1_ phase in a p53‐dependent manner. Moreover, three independent genetic screens uncovered that the activation of the p53‐p21 axis upon centrosome depletion relies on two additional factors, namely the oligomeric multidomain scaffold 53BP1 and the ubiquitin‐specific protease USP28 (Fong *et al*, 2016; Lambrus *et al*, 2016; Meitinger *et al*, 2016). 53BP1 and USP28 display the ability to form a ternary protein complex with p53 and support its ability to transactivate target genes such as p21 (Cuella‐Martin *et al*, 2016). This pathway is commonly called the mitotic surveillance pathway (MSP). The MSP is operational in somatic cell divisions and has measurable activity during embryonic development starting at around E7 in mice (Xiao *et al*, 2021). Moreover, in mouse models of microcephaly, in which mitotic delay in neural progenitor cells is achieved by deletion of centrosomal proteins, aberrant activation of the MSP appeared crucial to the etiology of the microcephalic phenotype (Phan *et al*, 2021; Phan & Holland, 2021). However, the mechanism governing MSP complex assembly and activity remains unclear.

Kinetochores (KTs) are multi‐protein assemblies built on centromeric loci, pivotal for the interactions between sister chromatids and the spindle microtubules (Pesenti *et al*, 2016). In addition to merely structural roles, they are important for SAC signaling, thereby defining the mitotic duration (Foley & Kapoor, 2013; Lara‐Gonzalez *et al*, 2021). Strikingly, 53BP1 was reported to localize to the outermost layer of KTs, known as the fibrous corona, the same substructure responsible for igniting SAC response (Jullien *et al*, 2002; Fong *et al*, 2016; Lambrus *et al*, 2016). Moreover, 53BP1 association with KTs appears to be dependent on the duration of mitosis, but independent of the SAC activation status (Fong *et al*, 2016). Whether the transient association of 53BP1 with KTs or its subsequent dissociation are important for MSP activation remains to be established. Here, we demonstrate that the fibrous corona protein CENP‐F and 53BP1 interact directly and that this is key to 53BP1 recruitment at the KT. Using gene editing, we introduced a single amino acid substitution engineering the endogenous *CENPF* locus in human cells. Leveraging this mutant, we show that KT‐localization of 53BP1 is not necessary for MSP functionality. More importantly, while PLK1 inhibition abnormally sequesters 53BP1 at KTs and prevents MSP activation in wild‐type cells, our *CENPF* mutant cells enabled us to show that cytosolic 53BP1 is able to bind p53 and to support MSP activation even in the absence of PLK1 activity. Thus, the MSP relies on a yet‐to‐be‐identified cytosolic clock.

## Results & Discussion

### CENP‐F is the 53BP1 KT receptor

**Figure EV1:**
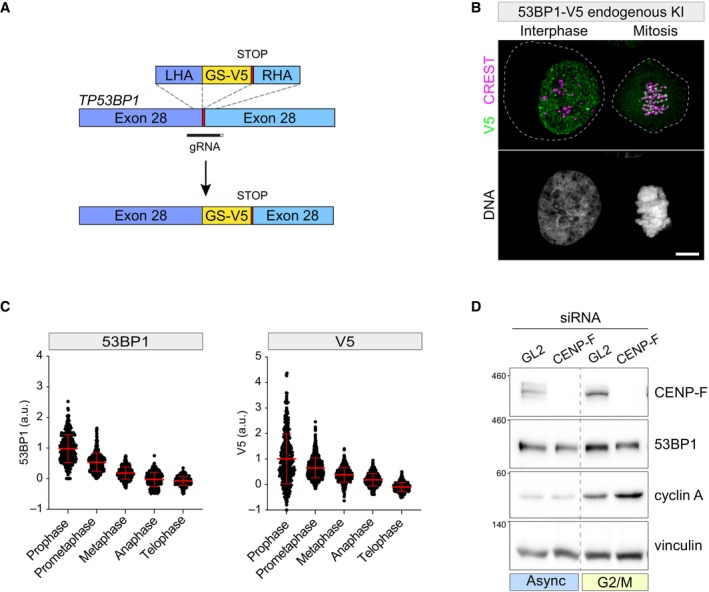
53BP1‐V5 endogenous knock‐in does not perturb 53BP1 localization and dynamics Schematic of the knock‐in strategy to introduce the V5 sequence into the endogenous *TP53BP1* locus. LHA: left homology arm; RHA: right homology arm; GS‐V5: Gly‐Ser linker followed by V5‐epitope tag.Representative fluorescence micrograph of 53BP1‐V5 cells co‐stained with the indicated antibodies. An interphase (left) and a mitotic cell (right) are shown. The dashed lines indicate the plasma membrane of the two cells. Scale bar: 5 μm.Dot plots showing 53BP1 (in RPE1 WT cells, left panel) or V5 (in RPE1 53BP1‐V5 cells, right panel) fluorescence intensity at individual KTs across the indicated cell cycle phases. Mean values (red lines) ± SD are reported, normalized on the prophase sample. *N* ≥ 349 KTs were assessed from 10 cells for each mitotic phase; a.u. = arbitrary units.HeLa S3 cells were transfected with the indicated siRNA and either treated with thymidine for 24 h and released in fresh medium for 10 h (G_2_/M), or left untreated (async = asynchronous). Cells were subjected to immunoblotting using the indicated antibodies. Source data are available online for this figure.

To unbiasedly characterize the KT receptor of 53BP1, we employed CRISPR/Cas9 (Ghetti *et al*, 2021) to engineer the endogenous *TP53BP1* locus of hTERT‐RPE1 cells (hereafter referred to as RPE1) introducing a biallelic V5‐epitope tag at the C‐terminus of the protein (Fig EV1A). V5‐tagged 53BP1 displayed a behavior indistinguishable from the untagged protein, showing a pan‐nuclear distribution with some discrete foci in interphase cells and being recruited at KTs upon mitotic entry (Fig EV1B). Moreover, the 53BP1‐V5 fusion protein mirrored the untagged one, accumulating at γH2AX‐positive foci in irradiated cells (Appendix Fig S1) and displaying similar mitotic phase‐dependent dynamics at the KT, peaking in prophase and gradually disappearing from this structure during mitotic progression (Fig EV1C). Thus, tagging of 53BP1 with V5 did not interfere with protein–protein interactions relevant to its dynamic localization.

**Figure 1:**
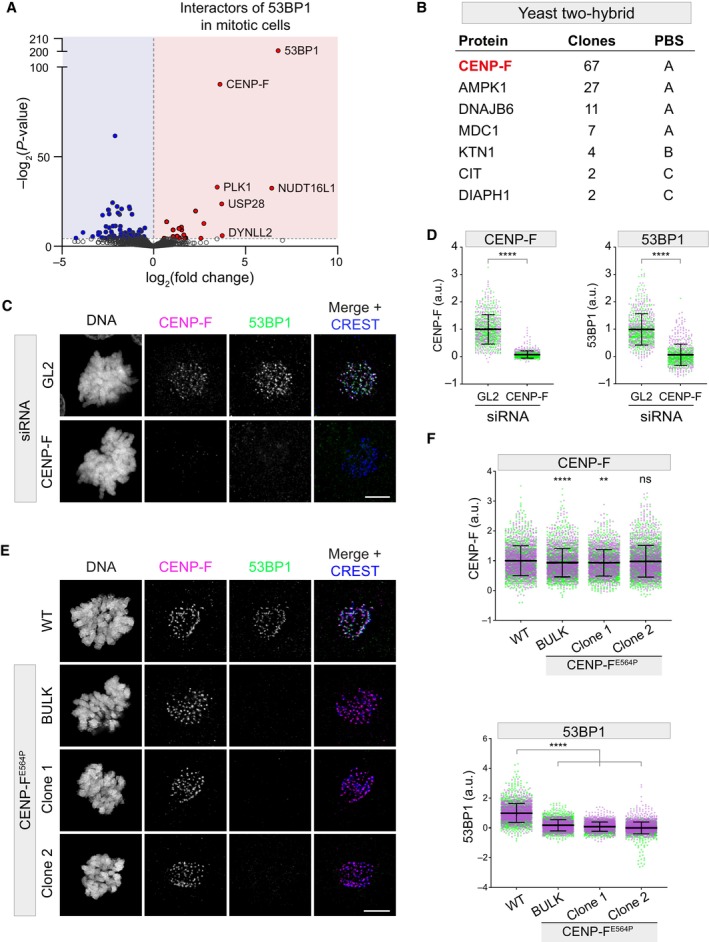
CENP‐F is the 53BP1 kinetochore receptor Volcano plot showing mitosis‐specific 53BP1 interacting proteins identified by mass spectrometry. Each dot depicts a single protein. The *x*‐axis represents the log_2_(fold change) over the untagged control; the *y*‐axis shows significance expressed as −log_10_(*P*‐value).List of the main prey fragments retrieved by the yeast two‐hybrid screen. The name of the protein, the number of clones, along with the specific protein binding score (PBS) are reported.HeLa S3 cells were transfected with the indicated siRNA and subjected to immunofluorescence using the indicated antibodies. Scale bar: 5 μm.Dot plots showing the intensity of the indicated proteins at individual KTs from images obtained as in (C). Each dot represents a particular KT. Mean values (black lines) ± SD (calculated on the entire dataset) are reported, normalized on the GL2 sample; a.u. = arbitrary units. *N* = 2 biological replicates are shown, one replicate in green, one in magenta. Significance was tested using an unpaired Mann–Whitney test (****P < 0.0001). The total number of KTs displayed is reported in the second tab of Dataset EV3.Representative fluorescent micrographs of RPE1 cells of the indicated genotype, co‐stained with the indicated antibodies. Scale bar: 5 μm.Dot plots showing the intensity of the indicated proteins at individual KTs from images obtained as in (E). Each dot represents a particular KT. Mean values (black lines) ± SD (calculated on the entire dataset) are reported, normalized on the WT sample; a.u. = arbitrary units. *N* = 2 biological replicates are shown, one replicate in green, one in magenta. Significance was tested using a Kruskal–Wallis test (****P < 0.0001; **P = 0.0019; n.s. = non‐significant). The total number of KTs displayed is reported in the second tab of Dataset EV3. Source data are available online for this figure.

To define the mitotic interactors of 53BP1, we synchronized RPE1 53BP1‐V5 knock‐in cells in prometaphase and performed immunoprecipitation against the V5‐tag, followed by LC–MS. MS analysis detected 21 specific binding partners (Dataset EV1), including some known 53BP1 interactors, such as NUDT16L1/TIRR, DYNLL2, USP28, and PLK1 (Fig 1A). The KT fibrous corona protein CENP‐F scored among the top enriched hits (Fig 1A), suggesting that it may play a role in 53BP1 localization at the KT. In an orthogonal search to define the KT‐binding partner(s) of 53BP1, we performed a yeast two‐hybrid screen using the KT‐binding domain of 53BP1 (Jullien *et al*, 2002) as bait against a human cDNA library. Of the 173 processed clones, 67 CENP‐F clones were identified at a very high confidence (Protein Binding Score of A) (Formstecher *et al*, 2005), thereby confirming that CENP‐F and 53BP1 are direct *bona fide* interactors (Fig 1B). Next, we investigated whether CENP‐F is necessary for 53BP1 recruitment to the KT. siRNA‐mediated depletion of CENP‐F in HeLa S3 cells disrupted 53BP1 localization at mitotic KTs (Fig 1C and D), without affecting 53BP1 protein levels (Fig EV1D).

**Figure EV2:**
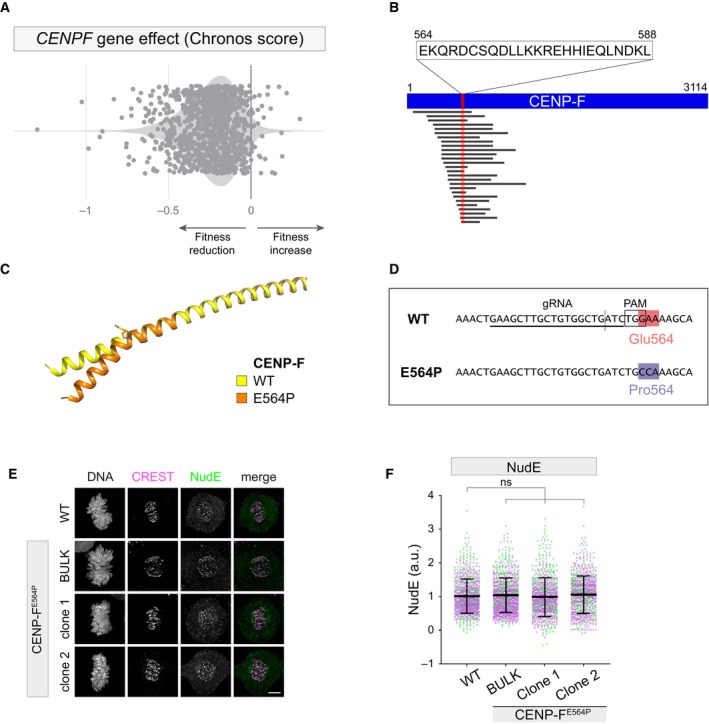
Targeted knock‐in strategy to interfere with 53BP1 localization at kinetochores Chronos gene dependency score of CENPF across 1,078 cell lines from DepMap database (CRISPR DepMap 22Q4). *x*‐axis: Chronos score.Schematic of the CENP‐F prey fragments retrieved by the yeast two‐hybrid screen and their relative position to the CENP‐F sequence. The overlap between all the different clones identifies a region of 25 amino acids as putative binding domain between the KT‐binding domain of 53BP1 (bait) and CENP‐F.AlphaFold modeling of the putative 53BP1 binding domain of CENP‐F. Introduction of a proline in position 564 is predicted to introduce a kink in this coiled coil region.Schematic depicting the knock‐in strategy used to introduce E564P mutation in CENPF. The gRNA recognition site, PAM sequence and cut site (dashed vertical line) are presented.Representative fluorescence micrographs of RPE1 cells of the indicated genotype, co‐stained with the indicated antibodies. Scale bar: 5 μm.Dot plots showing the intensity of NudE protein at individual KTs from images as in (E). Each dot represents a particular KT. Mean values (black lines) ± SD (calculated on the entire dataset) are reported, normalized on the WT sample; a.u. = arbitrary units. *N* = 2 biological replicates are shown, one replicate in green, one in magenta. Significance was tested using a Kruskal–Wallis test: n.s. = non‐significant. The total number of KTs displayed is reported in the second tab of Dataset EV3. Source data are available online for this figure.

CENP‐F is critical for assisting KT‐microtubule attachment and protecting corona cargoes from dynein‐dependent “stripping” (Bomont *et al*, 2005; Auckland *et al*, 2020). Consistent with this notion, the median Chronos gene score (Dempster *et al*, 2021) for *CENPF* knock‐out in 1,078 different cell lines analyzed in Depmap was −0.23, and the gene appeared essential in 113 of them (preprint: Dempster *et al*, 2019), demonstrating that complete knock‐out of *CENPF* confers a loss of fitness (Fig EV2A). Therefore, we sought a targeted strategy to inhibit CENP‐F binding to 53BP1. Thanks to our yeast two‐hybrid screen, we were able to restrict the CENP‐F minimal domain interacting with 53BP1 to a region of 25 residues, aa 564–588 (Fig EV2B), a region of the protein predicted to be part of a coiled‐coil motif (Ciossani *et al*, 2018). Thus, substituting the glutamic acid (E) at position 564 with proline (P, which destabilizes the α‐helical fold, Fig EV2C), could provide a strategy to selectively hinder protein–protein interactions relying on the proper folding of this coiled‐coil region. To this end, we used CRISPR/Cas9 to introduce the E564P substitution by modifying the endogenous *CENPF* locus of RPE1 cells (Fig EV2D) (Ghetti *et al*, 2021). Sequencing of the electroporated polyclonal population (hereafter referred to as bulk) confirmed the introduction of the desired mutation with a high editing rate at 5 days post‐electroporation (that is, > 80%, Appendix Fig S2). Bulk analysis on day 15 revealed that penetrance of the desired edit did not decrease over time (Appendix Fig S2). Moreover, homozygous monoclonal CENP‐F^E564P^ RPE1 derivatives were obtained from the polyclonal population (Appendix Fig S2).

CENP‐F^E564P^ mutant cells proved to be devoid of 53BP1 recruitment to mitotic KTs, phenocopying CENP‐F depletion (Fig 1E and F). Importantly, however, this point mutation did not abolish CENP‐F recruitment to the corona (Fig 1F). Although the CENP‐F^E564P^ mutation led to a statistically significant decrease of the CENP‐F signal at KTs in some of the edited cells (6.3 ± 3.5% for the bulk and 6.9 ± 3.9% in clone 1), this appears unlikely to be functionally relevant as neither CENP‐F ability to recruit its downstream effector protein NudE (Fig EV2E and F) nor mitotic proficiency were affected by this edit (Appendix Fig S3; Movie EV1). Thus, we precisely mapped the CENP‐F minimal domain required for 53BP1 recruitment to the KT and the introduction of a single amino acid substitution in the CENP‐F sequence allowed the generation of a separation‐of‐function mutant, which interferes with 53BP1 binding without impairing its other functions such as NudE KT recruitment.

### 53BP1 KT localization is dispensable for MSP functionality

**Figure 2:**
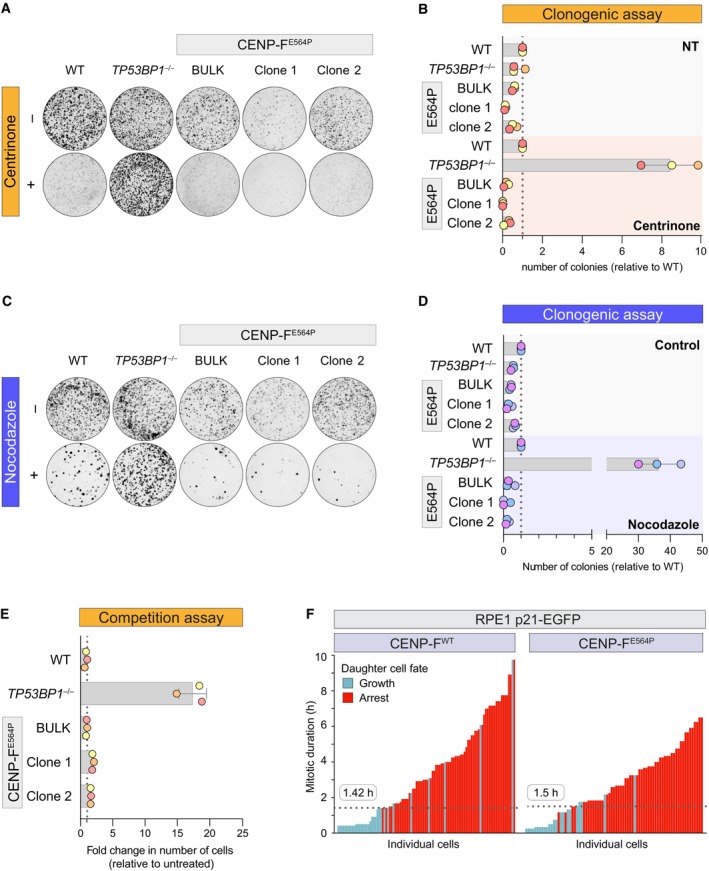
53BP1 kinetochore localization is dispensable for MSP functionality Cells of the indicated genotype were treated with centrinone for 12 days or left untreated (for 8 days) and stained with crystal violet. Images are representative of *N* = 3 biological replicates.Quantification of experiments as in (A). Data are presented as the number of colonies relative to WT cells (dotted line). The bars indicate the mean ± SD (*N* = 3 biological replicates).Pre‐synchronized cells of the indicated genotype were exposed to prometaphase arrest (+ nocodazole) or left untreated (− nocodazole), harvested, seeded for the clonogenic assay and stained with crystal violet after 12 and 8 days, respectively. Images are representative of *N* = 3 biologically independent experiments.Quantification of 3 independent experiments as in (C). Data are presented as the number of colonies relative to WT cells (dotted line). The bars indicate the mean ± SD (*N* = 3 biological replicates).Cells of the indicated genotype were mixed 1:1 with EGFP‐expressing WT cells and cultured either in the presence or absence of centrinone. After 8 days, the relative growth of each genotype was assessed by flow cytometry. The bars indicate the mean ± SD (*N* = 3 biological replicates) and values are expressed as relative to the WT sample (dotted line).RPE1 CENP‐F^WT^ and RPE1 CENP‐F^E564P^ cells were treated with dimethylenastron under the microscope, released from the drug and imaged for additional 2 days. The graph shows the daughter cell fate in relation to the time spent in mitosis by the mother cell. Each daughter cell is represented by a vertical bar, whose height represents the mitotic duration of its mother and the color indicating the fate of the daughter (growth or arrest). The dotted lines show the MSP threshold. *N* = 100 CENP‐F^WT^ and 77 CENP‐F^E564P^ cells. Source data are available online for this figure.

The generation of CENP‐F^E564P^ mutant cell lines provided a unique tool to precisely dissect the functional relevance of 53BP1 KT localization. We reasoned that 53BP1 recruitment at the fibrous corona could reflect a priming state for the MSP, similar to what happens for SAC proteins recruited to the same structure. However, we obtained strong evidence against this hypothesis. Activating the MSP with centrinone, a specific PLK4 kinase inhibitor causing centriole depletion over time, led to a measurable drop in the clonogenic potential of wild‐type (WT) RPE1 cells (Fig 2A and B). Expectedly, MSP deficiency (achieved by *TP53BP1* KO) clearly boosted the clonogenic potential in the presence of centrinone. Strikingly, however, this behavior was not matched in CENP‐F^E564P^, neither when utilizing the bulk population nor the homozygote clonal derivatives (Fig 2A and B). The same picture emerged from a competition‐based growth assay, where *TP53BP1* KO was the only genotype capable of weakening the cell cycle arrest promoted by centrinone, while CENP‐F^E564P^ mutant cells displayed an arrest that was at least as proficient as the one observed in WT cells (Fig 2E). Moreover, the functionality of the MSP in CENP‐F^E564P^ knock‐in cell lines could also be verified using a different trigger of the pathway, namely SAC activation, transiently exposing mitotic cells to nocodazole, promoting an extension of mitotic duration. Also in such experimental conditions, *TP53BP1* KO was the only genotype retaining high clonogenic potential, whereas extension of mitotic duration in CENP‐F^E564P^ knock‐in cell lines triggered a reduction of the clonogenic potential that was at least as pronounced as in WT cells (Fig 2C and D). Finally, we attempted to infer the MSP time threshold of CENP‐F^WT^ and CENP‐F^E564P^ cells. Using live cell imaging, we monitored the occurrence of p21 expression in relation to the time spent in mitosis, revealing no clear contribution of 53BP1 KT localization to the mitotic timer. Similar results were obtained upon CENP‐F knock‐down (Appendix Fig S4). Taken together, our data demonstrate that 53BP1 recruitment at the KT is not necessary for MSP function.

### PLK1 promotes 53BP1 loss of KT affinity

While our CENP‐F mutant cell lines afforded a way to displace 53BP1 from the KT, it was unclear whether it is possible to perturb the system in the opposite manner, namely forcing 53BP1 to remain at the KT. To gain better insight into this aspect, we characterized 53BP1 behavior during mitosis in greater detail.

**Figure EV3:**
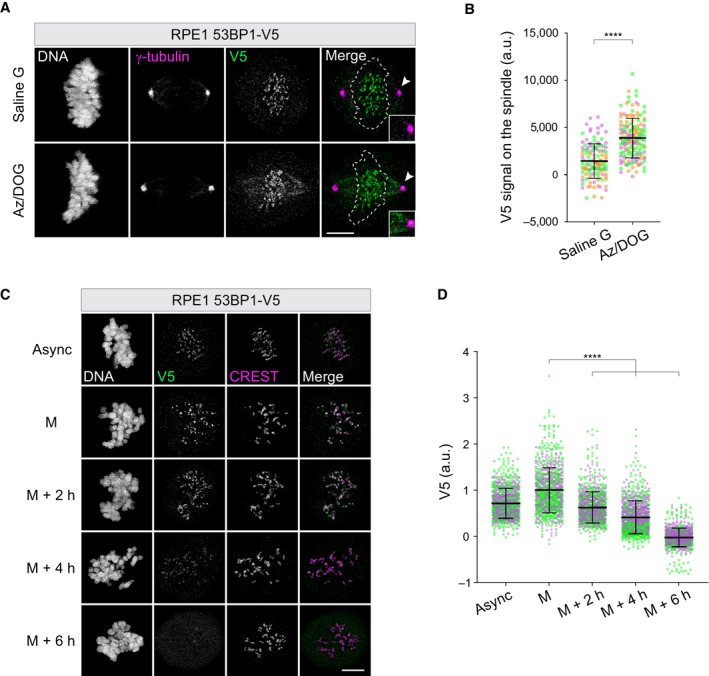
53BP1 is removed from kinetochores by stripping and a time‐dependent loss‐of‐affinity Representative fluorescence micrographs of RPE1 53BP1‐V5 cells incubated for 15 min in isotonic salt solution in the presence of either glucose (Saline G) or sodium azide/2‐deoxy‐D‐glucose (AZ/DOG) and co‐stained with the indicated antibodies. The blow‐up shows a portion of the mitotic spindle in proximity to one of the spindle poles (arrowhead). Scale bar: 5 μm.Dot plots showing V5 fluorescence intensity on the spindle obtained from images as in (A). Each dot represents a particular spindle. Mean values (black lines) ± SD (calculated on the entire dataset) are reported; a.u. = arbitrary units. *N* = 3 biological replicates are shown, one replicate in green, one in magenta, one in orange. Significance was tested using an unpaired *t*‐test (****P < 0.0001). The number of spindles measured is reported in the second tab of Dataset EV3.RPE1 53BP1‐V5 cells were either left untreated (async = asynchronous) or synchronized in prometaphase in medium containing nocodazole, fixed immediately (M = mitosis) or after 2, 4, or 6 h and co‐stained with the indicated antibodies. Scale bar: 5 μm.Dot plots showing V5 fluorescence intensity at individual KTs from images as in (C). Each dot represents a particular KT. Mean values (black lines) ± SD (calculated on the entire dataset) are reported, normalized on the mitotic (M) sample; a.u. = arbitrary units. *N* = 2 biological replicates are shown, one replicate in green, one in magenta. Significance was tested using a Kruskal–Wallis test (****P < 0.0001). The total number of KTs displayed is reported in the second tab of Dataset EV3. Source data are available online for this figure.

In agreement with previous studies (Jullien *et al*, 2002), 53BP1 displayed maximal association with mitotic KTs during prophase and then gradually dissociated in the subsequent phases (Fig EV1C). This localization pattern was similar to that reported for other corona‐localizing proteins. In fact, in the context of normal mitosis, the outermost fibrous corona layers are disassembled via dynein‐motor protein activity upon microtubule‐KT attachment, a process called stripping (Auckland *et al*, 2020). In a stripping hyperactivation assay (Howell *et al*, 2001), 53BP1 was removed from the KT and accumulated on the mitotic spindle (Fig EV3A and B; Appendix Fig S5), suggesting that, like CENP‐F (Auckland *et al*, 2020), 53BP1 is a dynein cargo during mitosis. Strikingly, however, in the context of a delayed mitosis in the presence of the microtubule‐poison nocodazole, the 53BP1 signal was still gradually lost over time, completely disappearing from KTs at around 6 h after mitotic entry (Fig EV3C and D). This suggests that in addition to stripping, 53BP1 is subjected to a time‐dependent loss of affinity for the KT.

**Figure 3:**
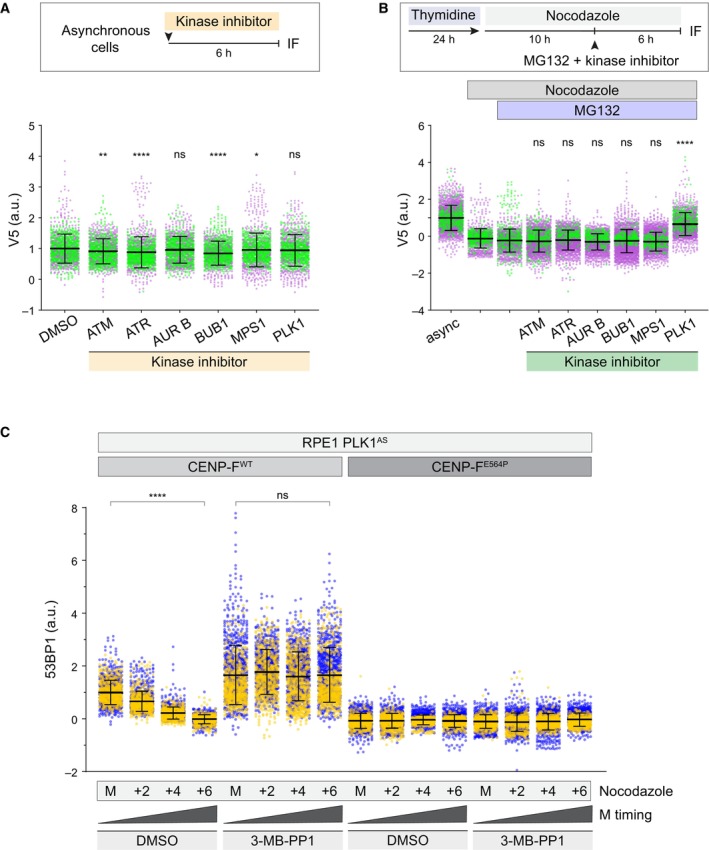
PLK1 activity induces 53BP1 loss of kinetochore affinity Top: Asynchronous RPE1 53BP1‐V5 cells were treated for 6 h with inhibitors for the indicated kinase (AUR B = Aurora B). Bottom: dot plots showing V5 fluorescence intensity at individual KTs. Each dot represents a particular KT. Mean values (black lines) ± SD (calculated on the entire dataset) are reported, normalized on the DMSO sample. IF = immunofluorescence; a.u. = arbitrary units. *N* = 2 biological replicates are shown, one replicate in green, one in magenta. Significance was tested using a Kruskal–Wallis test: ****P < 0.0001; **P = 0.0074; *P = 0.0333; n.s. = non‐significant. The total number of KTs displayed is reported in the second tab of Dataset EV3.Top: RPE1 53BP1‐V5 cells were either left untreated or treated with thymidine for 24 h and released in medium containing nocodazole. After 10 h, MG‐132 and the inhibitor for the indicated kinase were added. Bottom: dot plots showing V5 fluorescence intensity at individual KTs. Each dot represents a particular KT. Async = asynchronous cells; AUR B = Aurora B. Mean values (black lines) ± SD (calculated on the entire dataset) are reported, normalized on the asynchronous sample; a.u. = arbitrary units. *N* = 2 biological replicates are shown, one replicate in green, one in magenta. Significance was tested using a Kruskal–Wallis test: ****P < 0.0001, n.s. = non‐significant. The total number of KTs displayed is reported in the second tab of Dataset EV3.Cells of the indicated genotypes were synchronized in prometaphase in medium containing nocodazole, in the presence or absence of PLK1 inhibition (3‐MB‐PP1) and fixed immediately (M = mitosis) or after 2, 4, or 6 h. Dot plots show 53BP1 fluorescence intensity at individual KTs. Each dot represents a particular KT. Mean values (black lines) ± SD (calculated on the entire dataset) are reported, normalized on the WT early mitotic sample; a.u. = arbitrary units. *N* = 2 biological replicates are shown, one replicate in blue, one in yellow. Significance was tested using a Kruskal‐Wallis test: ****P < 0.0001; n.s. = non‐significant. The total number of KTs displayed is reported in the second tab of Dataset EV3. Source data are available online for this figure.

**Figure EV4:**
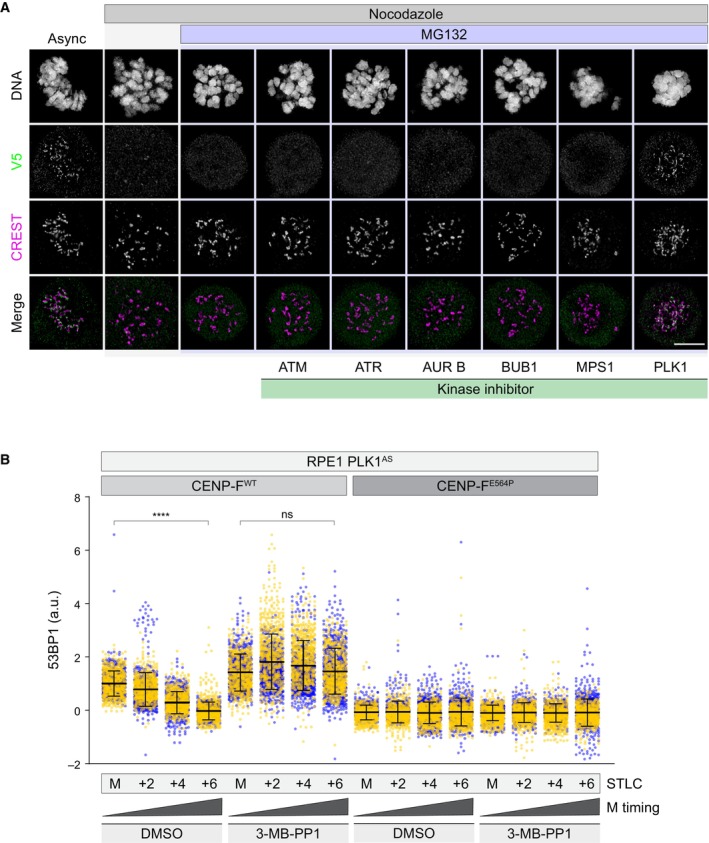
PLK1 control of 53BP1 kinetochore dynamics is independent of microtubule status Representative fluorescence micrographs of RPE1 53BP1‐V5 cells treated as in Fig 3B and co‐stained with the indicated antibodies. Async = asynchronous cells; AUR B = Aurora B. Scale bar: 5 μm.Cells of the indicated genotypes were synchronized in prometaphase in medium containing STLC, in the presence or absence of PLK1 inhibition (3‐MB‐PP1) and fixed immediately (M = mitosis) or after 2, 4, or 6 h. Dot plots show 53BP1 fluorescence intensity at individual KTs. Each dot represents a particular KT. Mean values (black lines) ± SD (calculated on the entire dataset) are reported, normalized on the WT early mitotic sample; a.u. = arbitrary units. *N* = 2 biological replicates are shown, one replicate in blue, one in yellow. Significance was tested using a Kruskal‐Wallis test: ****P < 0.0001; n.s. = non‐significant. The total number of KTs displayed is reported in the second tab of Dataset EV3. Source data are available online for this figure.

**Figure EV5:**
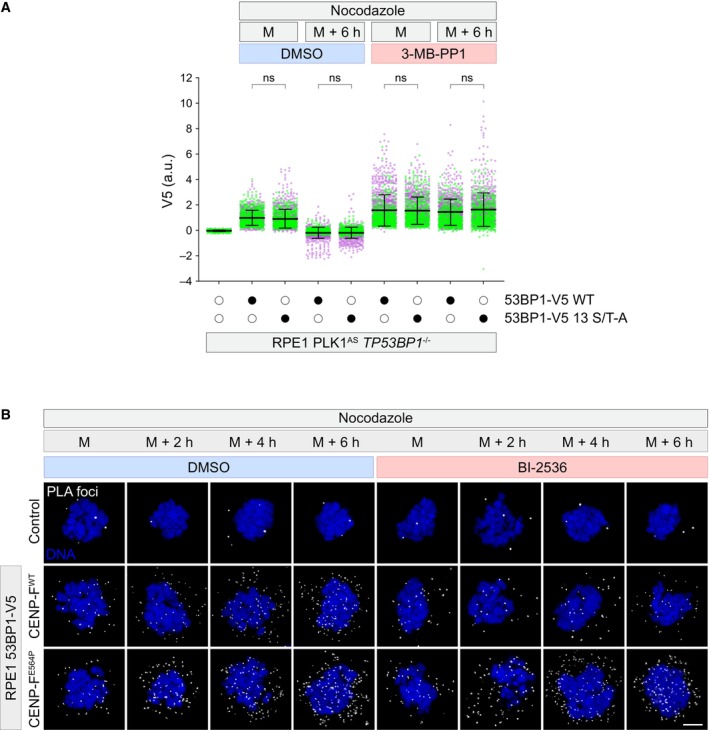
A 53BP1 phosphomutant displays intact PLK1‐dependent kinetochore dynamics RPE1 PLK1^AS^ *TP53BP1* KO cells were transduced with the indicated lentiviral vectors, synchronized in prometaphase in the presence or absence of PLK1 inhibition (3‐MB‐PP1) and fixed either immediately (M = mitosis) or after 6 h (M + 6 h). Dot plots show V5 fluorescence intensity at individual KTs. Each dot represents a particular KT. Mean values (black lines) ± SD (calculated on the entire dataset) are reported, normalized on the 53BP1 WT‐expressing sample at the earliest timepoint; a.u. = arbitrary units. *N* = 2 biological replicates are shown, one replicate in green, one in magenta. Significance was tested using a Kruskal‐Wallis test: n.s. = non‐significant. The total number of KTs displayed is reported in the second tab of Dataset EV3.Representative fluorescence micrographs of RPE1 cells of the indicated genotype, treated as in Fig 4A. M = mitosis. Scale bar: 5 μm. Source data are available online for this figure.

Phosphorylation events play crucial roles in mitotic KT assembly, dynamics and remodeling (Musacchio & Desai, 2017; Saurin, 2018). Moreover, 53BP1 is known to be hyperphosphorylated during mitosis (Jullien *et al*, 2002; Giunta *et al*, 2010; van Vugt *et al*, 2010; Lee *et al*, 2014). Therefore, we assessed whether the major known kinases targeting 53BP1 (i.e. ATM, ATR, PLK1, and Aurora B), along with master regulator kinases acting at the KT (MPS1 and BUB1), contribute to modulating the affinity of 53BP1 for the KT. Our mini‐screen showed that inhibition of none of the tested kinases abolished 53BP1 loading to KTs, although ATM, ATR, BUB1, and MPS1 inhibition caused a measurable loading reduction (Fig 3A; Appendix Fig S6). In a complementary setting, we also evaluated the contribution of the aforementioned kinases to 53BP1 loss of affinity for the KT. This analysis revealed that PLK1 has a striking contribution, as its inhibition abolishes the loss of affinity for the KT (Figs 3B and EV4A). To exclude any potential off‐target effect of the PLK1 inhibitor used in our mini‐screen, we took advantage of RPE1 cells in which endogenous PLK1 has been deleted and rely on the constitutive expression of a genetically modified allele (PLK1^AS^) that can be chemically inactivated by a bulky purine analogue (Burkard *et al*, 2007). To this end, we engineered the CENP‐F^E564P^ in this particular cell line. Also in this setting, inhibition of PLK1 in the context of MSP activating conditions was able to retain 53BP1 KT localization in WT cells but it did not restore the impaired localization of the protein in our CENP‐F^E564P^ mutant (Fig 3C; Appendix Fig S7). Importantly, delaying mitosis by inhibiting Eg5, a condition in which dynein‐mediated stripping is not prevented (Auckland *et al*, 2020), yielded results that are overlapping to those obtained with nocodazole (Fig EV4B; Appendix Fig S8). Surprisingly, 53BP1 did not appear to be the relevant PLK1 substrate in this process, as 53BP1 carrying the simultaneous mutation of 13 Ser/Thr to Ala in all theoretical PLK1 consensus sites retained the ability to undergo PLK1‐dependent KT loss of affinity (Fig EV5A; Appendix Fig S9). Taken together, while dynein‐mediated stripping might be an important component in an unperturbed cell division, in the presence of delayed anaphase 53BP1 requires PLK1 activity to display time‐dependent loss of KT affinity.

### PLK1 promotes 53BP1‐p53 association and MSP activation

**Figure 4:**
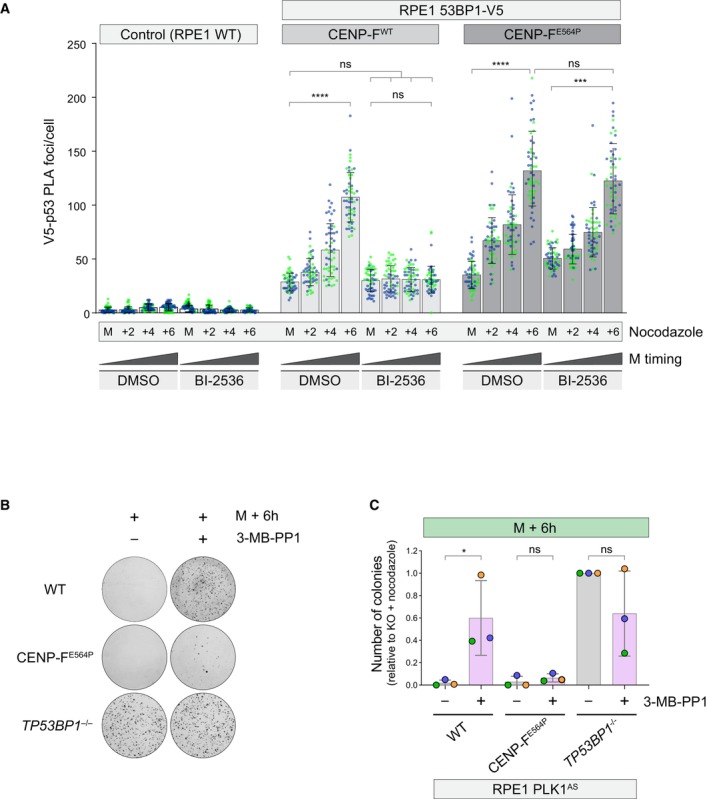
PLK1 activity is required for the MSP Cells of the indicated genotype were synchronized in prometaphase in medium containing nocodazole, in the presence or absence of PLK1 inhibition (BI‐2536) and fixed immediately (M = mitosis) or after 2, 4, or 6 h. Dot plots show the number of V5‐p53 PLA foci per cell. Mean values (black lines) ± SD (calculated on the entire dataset) are reported. *N* = 2 biological replicates are shown, one replicate in blue, one in green. Significance was tested using a Kruskal‐Wallis test: ****P < 0.0001; ***P = 0.0009; n.s. = non‐significant. The number of cells measured is reported in the second tab of Dataset EV3.RPE1 PLK1^AS^ cells of the indicated genotype were treated with thymidine for 24 h and released into medium containing nocodazole for 16 h (M + 6 h, M = mitosis), in the presence or absence of PLK1 inhibition (3‐MB‐PP1). Mitotic cells were selectively retrieved by shake‐off, washed‐out from the drugs, seeded for the clonogenic assay and stained with crystal violet after 16 days. Images are representative of *N* = 3 biologically independent experiments.Quantification of 3 independent experiments as in (B). Data are presented as the number of colonies relative to the *TP53BP1* ^−/−^ ‐ 3‐MB‐PP1 condition. The bars indicate the mean ± SD (*N* = 3 biological replicates). One‐way ANOVA test: *P = 0.0455; n.s. = non‐significant. Source data are available online for this figure.

PLK1 contribution in 53BP1 KT loss of affinity prompted us to evaluate whether forcing 53BP1 at the KT via PLK1 inhibition has an impact on MSP functionality. Firstly, as 53BP1 promotes p53 activity as a result of the formation of a ternary complex with USP28 and p53 itself (Cuella‐Martin *et al*, 2016), we set out to monitor the time‐dependent assembly of 53BP1‐p53 complexes in mitotically arrested cells. To this end, we leveraged our V5‐tagged 53BP1 cell line to perform proximity ligation assay (PLA). While cells exposed to mitotic delay showed a specific and time‐dependent accumulation of PLA signals, PLK1 inhibition prevented this phenomenon (Figs 4A and EV5B). Strikingly, CENP‐F^E564P^ cells also displayed time‐dependent accumulation of PLA signals, but, in contrast to CENP‐F^WT^ cells, they appeared insensitive to PLK1 inhibition, suggesting that PLK1 contributes to the formation of 53BP1‐p53 protein complexes by promoting 53BP1 loss of KT affinity.

Secondly, we exposed PLK1^AS^ CENP‐F^WT^ and CENP‐F^E564P^ cells to prometaphase delay in the presence or absence of the ATP analogue. We then released the cells and evaluated their long‐term proliferative capabilities by assessing their clonogenic potential in the absence of any drug. PLK1 inhibition during prometaphase arrest boosted the clonogenic potential of CENP‐F^WT^ cells upon drug wash‐out (Fig 4B and C). In stark contrast, when PLK1 inhibition was performed on CENP‐F^E564P^ cells, the clonogenic potential was no longer increased (Fig 4B and C). Taken together, we demonstrate that PLK1 controls both the capability of 53BP1 to associate with p53 in the mitotic cytosol as well as the long‐term proliferative potential of cells exposed to prometaphase delay. As both phenotypes are no longer present in CENP‐F^E564P^ cells, we suggest that PLK1 contribution lies in the promotion of time‐dependent 53BP1 loss of KT affinity.

**Figure 5:**
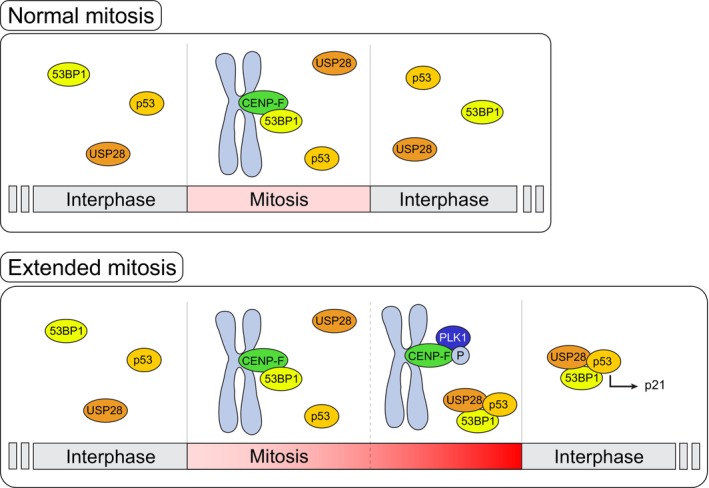
The mitotic surveillance pathway relies on a cytosolic clock In a normal mitosis (upper panel), 53BP1 is recruited to the KT via a direct interaction with CENP‐F. In the context of a prolonged mitosis (lower panel), PLK1 activity promotes the loss of affinity of 53BP1 for the KT (first priming step). Cytosolic 53BP1 needs an additional yet unidentified trigger to induce the formation of MSP complexes and USP28‐dependent deubiquitination of p53, which leads to cell cycle arrest (second priming step).

In the present study, we demonstrate that the loss of KT affinity displayed by 53BP1 during a prolonged prometaphase is PLK1‐dependent. Surprisingly, this does not seem to depend on direct 53BP1 phosphorylation by PLK1, but it is likely the result of phosphorylation of (an)other KT protein(s). Among them, CENP‐F, a known PLK1 substrate (Santamaria *et al*, 2010) and the 53BP1 KT receptor identified in our study, stands out as the best candidate to account for the observed dynamic behavior of 53BP1 (Fig 5).

PLK1 inhibition not only confines 53BP1 to KTs but also prevents its association with p53 in the mitotic cytosol. Furthermore, PLK1 activity in cells experiencing a single prolonged prometaphase appears to be important for constraining their clonogenic potential. While both phenotypes could have multiple explanations, they are reverted by the CENP‐F^E564P^ mutation. This suggests that PLK1 promotes the formation of MSP protein complexes and reduces the clonogenic potential of cells undergoing prolonged prometaphase by promoting 53BP1 loss of KT affinity.

One important conclusion of our study is that time measurement by the MSP does not directly involve the KT but it is a cytosolic process instead: an unknown mechanism favors the assembly of MSP protein complexes (53BP1 and p53 measured here by PLA, 53BP1:p53:USP28 measured by co‐immunoprecipitation in preprint: Meitinger *et al*, 2022). The physiological relevance of 53BP1 KT localization during normal mitosis instead remains to be established. Our attempt to highlight subtle differences in the MSP time threshold in the complete absence of 53BP1 KT localization failed to reveal a clear contribution in RPE1 cells. Despite this observation, we speculate that the transient 53BP1 sequestration at KTs might concur to provide an additional layer of regulation, acting as a fail‐safe mechanism to ensure a stringent and reproducible time threshold. Further studies will have to experimentally tackle this issue.

## Materials and Methods

### Cell culture

hTERT‐RPE1 cells (a gift of Stephan Geley, Medical University of Innsbruck), hTERT‐RPE1 PLK1^AS^ cells (a gift of Prasad Jallepalli, Memorial Sloan Kettering Cancer Center) and hTERT‐RPE1 p21‐EGFP cells (a gift of Chris Bakal, Institute of Cancer Research) were cultured in DMEM/F12 1:1 (Gibco). HEK 293T (a gift from Ulrich Maurer, University of Freiburg) and HeLa S3 (a gift from Erich Nigg) cells were maintained in DMEM (Gibco). All media were supplemented with 10% fetal bovine serum (Gibco), 2 mM L‐glutamine (Corning) and penicillin–streptomycin solution (Corning). Cells were grown at 37°C with 5% CO_2_ and routinely tested for mycoplasma contamination.

### Drug treatments, irradiation, and synchronization procedure

The following compounds were used: 1 μM NU‐7441 (Selleck Chemicals), 125 nM centrinone (MedChem Express), 2 mM thymidine (Abcam), 3.3 μM nocodazole (MedChem Express), 2 μM dimethylenastron (Selleck Chemicals), 100 nM BI‐2536 (MedChem Express), 500 nM reversine (Enzo Life Sciences), 10 μM KU‐60019 (MedChem Express), 10 μM VE‐822 (MedChem Express), 2 μM ZM‐447439 (Selleck Chemicals), 10 μM BAY‐524 (MedChem Express), 10 μM MG‐132 (Selleck Chemicals), 10 μM 3‐MB‐PP1 (Cayman Chemical), 5 μM S‐trityl‐L‐cysteine (STLC, Tocris Bioscience). Thymidine was dissolved in water whereas all other drugs were dissolved in DMSO; to all untreated controls solvent only was administered. Cells were irradiated with 2 Gy (delivering at a dose rate of 1.6 Gy/min) of ionizing radiation using the Xstrahl RS225 X‐ray research Irradiator (West Midlands, UK) and allowed to recover for 30 min prior to fixation for immunofluorescence. Synchronization of RPE1 was performed by arresting cells with thymidine for 24 h, followed by release in fresh medium containing nocodazole for either 10 or 16 h. Mitotic cells were then harvested by selective shake‐off and either directly lysed or washed four times and released into fresh medium. For the ATP depletion assay (Howell *et al*, 2001), cells were rinsed in saline (140 mM NaCl, 5 mM KCl, 0.6 mM MgSO_4_, 0.1 mM CaCl_2_, 1 mM Na_2_HPO_4_, 1 mM KH_2_PO_4_, pH 7.3) and placed in either saline G (saline + 4.5 g/l D‐glucose) or Az/DOG (saline + 5 mM sodium azide + 1 mM 2‐deoxy‐D‐glucose) for 15 min at 37°C.

### siRNA‐mediated gene knock‐down

HeLa cells were transfected with 40 nM CENP‐F siRNA (5′‐CAAAGACCGGUGUUACCAAG‐3′) or luciferase (GL2) siRNA (5′‐CGUACGCGGAAUACUUCGA‐3′) whereas RPE1 cells where transfected with siRNA SMART pool targeting CENP‐F (Dharmacon, L‐003253‐00‐0005) or a non‐targeting control (Dharmacon, D‐001810‐01‐05) using Lipofectamine RNAiMAX Transfection Reagent (Life Technologies), according to the manufacturer's procedures. Forty‐eight hours post‐transfection, cells were either harvested for Western blotting or fixed for immunofluorescence analysis.

### Molecular cloning of phosphomutant 53BP1

Thirteen PLK1 phosphorylation sites on 53BP1 were mapped according to the following PLK1 consensus sequence: L(Φ) (E/N/D(Q)) X (S/T) L(Φ) (Santamaria *et al*, 2010). To generate a 53BP1 phosphomutant (13 S/T‐A), pcDNA5‐FRT/TO‐eGFP‐53BP1 (Addgene plasmid #60813) was used as a template to perform site‐directed mutagenesis (Edelheit *et al*, 2009) with the primers listed in Dataset EV2. Both wild‐type and mutant 53BP1 cDNAs were subcloned into the lentiviral plasmid FUW‐tetO‐MCS+ (a modified version of Addgene plasmid #84008, gift by Dr. Alessio Zippo), in frame with a V5‐tag. Generation of lentiviral particles, titration, and transduction were performed as previously described (Burigotto *et al*, 2021).

### AlphaFold molecular modeling

CENP‐F models for WT and the E564P mutant were obtained through ColabFold v1.5.2 (Mirdita *et al*, 2022). Sequence boundaries (aa 546–780) were chosen to include all residues predicted in the AlphaFoldDB (Jumper *et al*, 2021) in the single α‐helix comprising Glu564.

### Engineering of *TP53BP1* and *CENPF* endogenous loci

To generate RPE1 53BP1‐V5 knock‐in cells and to introduce the CENP‐F^E564P^ mutation in the RPE1 WT, RPE1 PLK1^AS^ and RPE1 p21‐EGFP cell lines, 2 × 10^5^ cells were electroporated using Lonza Nucleofector 4‐D according to manufacturer's instruction and as previously described (Ghetti *et al*, 2021). Briefly, 100 μM of crRNA (IDT) and 100 μM of tracRNA (IDT) were annealed to form gRNAs. Ribonucleoparticles (RNPs) were complexed by mixing 120 pmol of recombinant Cas9 and 150 pmol of gRNAs. Electroporation mix was prepared resuspending RPE1 cells in P3 Primary Cell Full Electroporation Buffer (Lonza), and adding previously prepared RNPs, 4 μM Alt‐R Cas9 Electroporation enhancer (IDT) and 4 μM single‐strand DNA homology template (Ultramer DNA Oligonucleotide, IDT). After electroporation, cells were treated with 1 μM NU‐7441 (Selleck Chemicals) for 48 h and single‐cell clones were obtained by dilution cloning. Genomic DNA was extracted using NucleoSpin Tissue columns (Macherey‐Nagel). Sanger sequencing of PCR products spanning the insertion sites confirmed the correct in‐frame insertion of the V5 tag (*TP53BP1* locus) or E564P mutation (*CENPF* locus) in homozygosis. RPE1 53BP1‐V5 CENP‐F^E564P^ double knock‐in cells were generated by two consecutive rounds of electroporation and single‐cell cloning, starting from the V5‐tagged cell line. For crRNA sequences, DNA homology donors and PCR primers see Dataset EV2.

### Generation of *TP53BP1* KO cell lines

RPE1 *TP53BP1* KO cells were generated using a lentiviral‐based CRISPR/Cas9 strategy, as previously described (Burigotto *et al*, 2021). Briefly, a gene‐specific crRNA was cloned into the Lenti‐CRISPR‐V2 backbone (gift from Feng Zhang; Addgene plasmid #52961). This plasmid, along with pCMV‐VSV‐G (a gift from Bob Weinberg, Addgene plasmid #8454) and psPAX2 (a gift from Didier Trono, Addgene plasmid #12260), was used to co‐transfect HEK 293T cells using calcium phosphate. After 48 h, supernatants were harvested, filtered, mixed with 4 μg/ml hexadimethrine bromide (Sigma‐Aldrich) and administered to cells for 24 h. Transduced cells were enriched by 10 μg/ml puromycin selection (InvivoGen) for 72 h. RPE1 PLK1^AS^ *TP53BP1* KO cells were generated using an RNP‐based CRISPR/Cas9 strategy (Ghetti *et al*, 2021), as described above (without the addition of a single‐strand DNA homology template nor NU‐7741 treatment). For both cell lines, isogenic clones were isolated by limiting dilution. The presence of gene‐disrupting INDELs in edited cells was confirmed by Sanger sequencing of PCR products spanning the crRNA recognition site, followed by Inference of CRISPR Edits (ICE) analysis (https://ice.synthego.com) (preprint: Hsiau *et al*, 2018).

### Yeast two‐hybrid screen

The yeast two‐hybrid screen was performed by Hybrigenics Services, S.A.S., Evry, France. The KT binding domain sequence of 53BP1 (aa 1,235–1,616) was PCR‐amplified from pcDNA5‐FRT/TO‐eGFP‐53BP1 (Addgene plasmid #60813) and cloned into pB66 downstream to the Gal4 DNA‐binding domain. This construct was used as bait to screen a human lung cancer cells cDNA library constructed into pP6. Seventy million clones were screened using a mating approach with YHGX13 (Y187 ade2‐ 101::loxP‐kanMX‐loxP, matα) and CG1945 (mata) yeast strains as previously described (Fromont‐Racine *et al*, 1997). One hundred seventy‐three His^+^ colonies were selected on a medium lacking tryptophan, leucine, and histidine. Prey fragments of positive clones were amplified by PCR and sequenced at their 5′ and 3′ junctions. The resulting sequences were used to identify the corresponding interacting proteins in the GenBank database (NCBI) using a fully automated procedure. The sequences of 67 CENP‐F fragments were overlapped and their positions calculated relative to the full‐length protein sequence. The minimal region that overlaps represents the selected interacting domain.

### Clonogenic assay

4 × 10^3^ RPE1 cells were seeded in 10‐cm dishes and medium was refreshed every 5 days. When applied, centrinone was refreshed every 3 days. For Fig 2C and D, cells were treated with thymidine for 24 h and released in medium containing nocodazole. Mitotic cells (M + 6 h) were selectively retrieved by shake‐off, released from nocodazole and seeded for the clonogenic assay. In parallel, control cells were synchronized by a thymidine block, released in fresh medium for 16 h, detached by trypsinization and seeded for the assay. After 8–16 days, cells were washed in PBS and then stained for 30 min at 37°C with crystal violet (0.2% w/v, AcrosOrganics) dissolved in 20% v/v methanol. Cell plates were rinsed thoroughly with water and left to dry overnight. Images were acquired using a ChemiDoc Imaging System (Bio‐Rad).

### Cell lysis and Western blot

Cells were harvested by trypsinization and lysed in 50 mM Tris pH 7.4, 150 mM NaCl, 0.5% v/v NP‐40, 50 mM NaF, 1 mM Na_3_VO_4_, 1 mM PMSF, one tablet/50 ml cOmplete, EDTA‐free Protease Inhibitor Cocktail (Roche), 2 mM MgCl2 and 0.2 mg/ml DNase I (Thermo Fisher Scientific). Protein concentration was assessed by bicinchoninic acid assay (Pierce BCA Protein Assay Kit, Thermo Fisher Scientific) using a plate reader. Equal amounts of total protein samples (40–50 μg) were resolved by polyacrylamide gels using self‐made or pre‐cast gels (Bio‐Rad). Proteins were electroblotted on nitrocellulose membranes (Cytiva) using a wet transfer system (Bio‐Rad). Membranes were blocked in blocking solution (5% w/v non‐fat milk in PBS‐Tween 0.1% v/v), incubated overnight at 4°C with the relevant antibody diluted in blocking solution, washed several times with PBS‐Tween 0.1% v/v and then incubated at room temperature for 50 min with HRP‐conjugated secondary antibodies diluted in blocking solution. Protein detection was carried out using an Alliance LD2 Imaging System (UviTec Cambridge), after incubating membranes with Amersham ECL Select Western Blotting Detection Reagent (Cytiva). The following antibodies were used: rabbit anti‐CENPF (Cell Signaling Technology, 58982, 1:500), rabbit anti‐53BP1 (Cell Signaling Technology, 4937, 1:500), mouse anti‐cyclin A2 (Abcam, ab38, 1:500), mouse anti‐vinculin (Sigma‐Aldrich, V9264, 1: 2,000), goat anti‐rabbit IgG/HRP (Dako, P0448, 1:5,000), rabbit anti‐mouse IgG/HRP (Dako, P0161, 1:5,000).

### Immunoprecipitation and MS analysis

WT and 53BP1‐V5 tagged RPE1 cells were grown in 15‐cm dishes, arrested in thymidine and released in nocodazole for 10 h. Mitotic shake‐off was performed, cells were washed with PBS and lysed as described above. Seven hundred microgram of each sample were incubated with 30 μl of slurry Affi‐Prep protein A resin (Bio‐Rad) and 1 μg of mouse anti‐V5 tag antibody (Invitrogen, R96025) for 4 h at 4°C. The beads were collected by centrifugation and washed twice with lysis buffer. Dried bead complexes were eluted by boiling samples in Bolt™ LDS sample buffer with 10% v/v Bolt™ Sample Reducing Agent (Thermo Fisher Scientific) at 95°C for 10 min. Samples were separated in precast 10% Bolt Bis‐Tris Plus gel (Thermo Fisher Scientific), run for ∼1 cm and stained with a Coomassie solution. For each sample, the entire stained area was excised and washed with 100 mM NH_4_HCO_3_ in 50% v/v acetonitrile (ACN) for 15 min. The colorless gel plugs were then dehydrated by adding 100% ACN. The dried gel pieces were reduced with 10 mM DTT for 1 h and alkylated with 55 mM iodoacetamide for 30 min. Gel fragments were then washed in water, dehydrated with ACN, and incubated in a digestion solution containing 12.5 ng/μl trypsin (Thermo Fisher Scientific) in 100 mM NH_4_HCO_3_ at 37°C overnight. Peptides were extracted by sequentially treating gel pieces with 3% trifluoroacetic acid (TFA) in 30% v/v ACN, and 100% v/v ACN. Tryptic peptides were then dried in a speed‐vac and acidified with TFA to a pH < 2.5. After desalting on C18 stage tips, peptides were resuspended in 0.1% v/v formic acid (FA) for LC–MS/MS analysis.

Peptides were separated on an Easy‐nLC 1200 HPLC system (Thermo Scientific) using a 25 cm reversed‐phase column (inner diameter 75 μm packed in‐house with ReproSil‐Pur C18‐AQ material: 3 μm particle size, Dr. Maisch, GmbH) with a two‐component mobile phase (0.1% v/v FA in water and 0.1% v/v FA in ACN). Peptides were then eluted using a gradient of 5–25% over 50 min, followed by 25–40% over 15 min and 40–98% over 10 min at a flow rate of 400 nl/min. Peptides were analyzed in an Orbitrap Fusion Tribrid mass spectrometer (Thermo Fisher Scientific) in data‐dependent mode, with a full‐scan performed at 120,000 FWHM resolving power (mass range: 350–1,100 *m/z*, AGC target value: 10e6 ions, maximum injection time: 50 ms), followed by a set of (HCD) MS/MS scans over 3 s cycle time at a collision energy of 30% (AGC target: 5 × 10e3 ions, maximum injection time: 150 ms). Dynamic exclusion was enabled and set at 30 s, with a mass tolerance of 5 ppm. Data were acquired using Xcalibur 4.3 software and Tune 3.3 (Thermo Scientific). For all acquisitions, QCloud (Chiva *et al*, 2018) was used to control instrument longitudinal performance during the project using in‐house quality control standards. Raw files were searched using Proteome Discoverer v.2.2.0 (Thermo Scientific). Peptide searches were performed against the in‐silico digested UniProt Human database (downloaded April 2021), added with major known contaminants and the reversed versions of each sequence. Trypsin/P was chosen as the enzyme with 5 missed cleavages, and static modification of carbamidomethyl (C) with variable modification of oxidation (M) and acetylation of protein N‐terminus were incorporated in the search. The MASCOT search engine (v.2.6.2, MatrixScience) was used to identify proteins (precursor mass tolerance: 10 ppm, product mass tolerance: 0.6 Da). The FDR was set to < 0.01 at both the peptide and protein levels. Peak intensities of the peptides were log_2_ transformed and data were normalized on the average of the specific protein abundance within each sample (Aguilan *et al*, 2020). The fold change (FC) of each peptide was calculated. Then, the FC at protein level was calculated by averaging the FC of all peptides assigned to each protein. 53BP1‐binding partners were identified by subtracting the log_2_‐normalized intensities of the control sample (RPE1 WT mitotic cells) to the test sample (RPE1 53BP1‐V5 mitotic cells). Statistical significance was assessed using Student's *t*‐test (two‐tailed, two‐sample unequal variance).

### Competition assay and FACS analysis

To generate cells constitutively expressing EGFP, the EGFP sequence was PCR amplified from pEGFP‐N1 (Clontech), and cloned in the pAIB‐CAG lentiviral vector (a gift from Claudio Ballabio) between the PmeI and NotI sites, upstream of an IRES element and a blasticidin resistance gene under the CAG promoter. Lentiviral particles were produced as described above. After transduction, cells were treated with 5 μg/ml blasticidin for 7 days. For competition growth assays, RPE1 WT cells stably expressing EGFP and non‐fluorescent RPE1 cells of the desired genotype were mixed at a 1:1 ratio and seeded into duplicate wells. One well from each pair was treated with centrinone for 8 days. Cells were analyzed on a Symphony A1 cytometer (BD Biosciences) to assess the percentage of EGFP^+^ cells. Live cells were gated through forward‐scatter (FSC) and side scatter (SSC) parameters and cell doublets were excluded. For each sample, the fraction of EGFP‐ cells was divided by the fraction of EGFP^+^ cells. The value obtained from the centrinone treated well was then divided by that obtained in the untreated well to determine the fold change in EGFP‐ cells. Analysis of flow cytometry data was performed using FlowJo software (FlowJo, LLC).

### Immunofluorescence

Cells were grown on glass coverslips (Marienfeld‐Superior), washed in PBS and directly fixed and permeabilized with absolute ice‐cold methanol for at least 20 min at −20°C. For NudE staining cells were pre‐extracted for 2 min in PTEM buffer (0.2% v/v Triton X‐100, 20 mM PIPES at pH 6.8, 1 mM MgCl_2_, 10 mM EGTA in ddH_2_O) and then fixed with 4% v/v formaldehyde (Sigma‐Aldrich) in PTEM for 10 min at room temperature. Cells were rinsed with PBS, blocked with 3% w/v BSA in PBS for 20 min and stained for 1 h at room temperature with primary antibodies diluted in blocking solution. Cells were washed with PBS and incubated with fluorescent secondary antibodies for 45 min at room temperature. DNA was stained with 1 μg/ml Hoechst 33342 (Invitrogen). After incubation, cells were rinsed with PBS and ddH2O, and mounted using ProLong Gold Antifade Reagent (Invitrogen). The following antibodies were used: mouse anti‐53BP1 (Millipore, MAB3802, 1:500), mouse anti‐V5 (Invitrogen, R96025, 1:1,000), rabbit anti‐V5 tag (Cell Signaling Technology, #13202, 1:1,000), rabbit anti‐CENP‐F (Cell Signaling Technology, 58982, 1:500), human anti‐centromere/KT (Antibodies Inc, 15‐234, 1:500), rabbit anti‐NudE (ProteinTech, 10233‐1‐AP, 1: 250), mouse anti‐phospho‐Histone H2A.X Ser139 (Millipore, 05‐636, 1:500), mouse anti γ‐tubulin (Thermo Fisher Scientific, MA1‐19421), goat anti‐mouse AlexaFluor488 (Invitrogen, A11029, 1:1,000), goat anti‐mouse AlexaFluor 555 (Invitrogen, A21424, 1:1,000), goat anti‐rabbit AlexaFluor 488 (Invitrogen, A11034, 1:1,000), goat anti‐rabbit AlexaFluor 555 (Invitrogen, A21429, 1:1,000), goat anti‐human AlexaFluor 647 (Invitrogen, A21445, 1:1,000). For Appendix Fig S4B, a sheep anti‐CENP‐F antibody (kind gift of S. Taylor, University of Manchester, SCF.1, 1:1,000) was used. Images in Figs 1C, EV1B and EV2E were acquired on a spinning disc Eclipse Ti2 inverted microscope (Nikon Instruments Inc), equipped with Lumencor Spectra X Illuminator as LED light source, an X‐Light V2 Confocal Imager and an Andor Zyla 4.2 PLUS sCMOS monochromatic camera using a plan apochromatic 100×/1.45 oil immersion objective. Images in Appendix Fig S4B were collected using a Deltavision Elite system (GE Healthcare) controlling a Scientific CMOS camera (pco.edge 5.5). Acquisition parameters were controlled by SoftWoRx suite (GE Healthcare). Images were collected using an Olympus 100×/1.4 oil immersion objective. Images in Appendix Fig S1A, C and E were acquired on a Nikon AX confocal microscope (Nikon Instruments Inc) equipped with a LUA‐S4 laser unit using a plan apochromatic 100×/1.49 oil immersion objective. Images were deconvolved with Huygens Professional software (Scientific Volume Imaging, Hilversum, The Netherlands). All remaining images were acquired on a Leica TCS SP8 microscope using a 63×/1.4 oil objective with Lightening mode (adaptive as “Strategy” and ProLong Gold as “Mounting medium”) to generate deconvolved images. Images were processed using Fiji and displayed as maximum intensity projections of deconvolved z‐stacks. All displayed images were selected to most closely represent the mean quantified data.

### *In situ* proximity ligation assay (PLA)

RPE1 53BP1‐V5 CENP‐F^WT^, RPE1 53BP1‐V5 CENPF^E564P^ and RPE1 WT were grown on glass coverslips, washed in PBS and fixed with ice‐cold methanol for 20 min at −20°C. Cells were rinsed with PBS, blocked with 3% w/v BSA in PBS (blocking buffer) for 20 min and then incubated for 1 h at room temperature with mouse anti‐V5 antibody (Invitrogen, R96025) and rabbit anti‐p53 antibody (Cell Signaling Technology, #9282), diluted 1:500 in blocking buffer. PLA (Söderberg *et al*, 2008) was performed using the Duolink kit, according to the manufacturer's protocol. Briefly, samples were incubated with anti‐mouse Minus probe (DUO92004, Sigma) and anti‐rabbit Plus probe (DUO92002, Sigma) diluted in blocking buffer for 1 h at 37°C. Cells were rinsed twice with Wash Buffer A (DUO82049, Sigma), incubated with a ligation‐ligase solution for 30 min at 37°C and then washed twice in Wash Buffer A. To each sample an amplification‐polymerase solution (DUO92007, Sigma) was added and they were incubated for 100 min at 37°C. Cells were rinsed with Wash Buffer B (DUO82049, Sigma) and coverslips were mounted using Duolink *in Situ* Mounting Medium with DAPI (DUO82040, Sigma). All incubation steps were performed in the dark inside a humidity chamber. To quantify PLA spots, cells were manually segmented using Fiji and the “find maxima” function was then applied to quantify the number of foci per cell.

### Time‐lapse video microscopy

Cells were seeded into Ibidi μ‐Slide 8 Well dishes (Ibidi) in DMEM/F12 medium without phenol red the day before imaging. One hour before imaging, 1 μM SiR‐DNA (Spirochrome, SC007) was added to visualize DNA. To assess SAC proficiency, 1 h before imaging, culture medium was replaced with medium containing 3.3 μM nocodazole or DMSO. Movies were recorded every 3 min for 20 h (to measure mitotic timing) or every 5 min for 48 h (to test SAC proficiency). Cells were imaged on a spinning disc Eclipse Ti2 inverted microscope (Nikon Instruments Inc), equipped with Lumencor Spectra X Illuminator as LED light source, an X‐Light V2 Confocal Imager and an Andor iXon Ultra 888 EMCCD camera using a plan apochromatic 20×/0.75 objective. The representative movies were deconvolved with Huygens Professional software (Scientific Volume Imaging, Hilversum, The Netherlands).

### Mitotic surveillance pathway threshold assay

RPE1 p21‐EGFP CENP‐F^WT^ and CENP‐F^E564P^ cells were seeded into 4‐well chamber slides (Ibidi) 24 h before imaging. For the experiment in Appendix Fig S4, RPE1 p21‐EGFP cells were treated with a siRNA SMART pool targeting CENP‐F or a non‐targeting control for 24 h before seeding them in imaging chambers. Before imaging, standard media was replaced with CO_2_‐independent base medium (Thermo Fischer) and cells were maintained at 37 °C in an environmental control station. Once under the microscope, cells were blocked in mitosis with 2 μM dimethylenastron for 7 h. Fresh media was then replaced to allow cells to exit mitosis and cells were monitored for 2 additional days. Daughter cell fate (growth/arrest) was inferred using p21‐EGFP expression (Appendix Fig S4D) and related to time spent in mitosis (NEBD‐anaphase onset). Long‐term time‐lapse imaging was performed using a Deltavision Elite system (GE Healthcare) controlling a Scientific CMOS camera (pco.edge 5.5). Images were acquired with an Olympus 40× 1.4 NA oil objective. To assess the MSP threshold, cells were sorted by ascending mitotic duration, then scanned from short to long mitoses with a 10‐cell window. Once identified a window containing ≥ 50% arrested cells, the threshold was measured as the average of the two middle points.

### Image analysis and statistics

All images of similarly stained experiments were acquired with identical illumination settings. To quantify fluorescence intensity at KTs, maximum intensity projections of z‐stacks were obtained using Fiji and circular regions of interest (ROIs) with a diameter of 5 pixels were drawn, centered on the KT (CREST signal). Measurements were performed on at least 10 independent images. For each cell, 3 background ROIs (placed in the cytoplasmic space, in proximity to the DNA staining) were drawn and their average value was subtracted from the intensity value of each KT. To quantify DNA damage foci, cell nuclei were segmented using Fiji, according to the Hoechst signal. The “find maxima” function was then applied to quantify the number of foci inside each nucleus. To quantify 53BP1 signal in the stripping assay, 2 binary masks for γ‐tubulin signal (signal at poles and total signal) and a DNA mask were generated (Appendix Fig S5). To the total γ‐tubulin mask, the pole mask and the DNA mask were subtracted. The resulting ROI was used to measure 53BP1 fluorescence intensity in the region of the mitotic spindle external to the chromosome mass and not comprising the two centrosomes. To quantify the number of colonies in the clonogenic assay, plates scans were imported in Fiji, manually cropped to individual plates and median filtered. Colonies were equally segmented among different genotypes and treatments, and the “analyze particles” function was then used to count colonies. Data are presented either as dot plots or as bar charts and mean ± standard deviation (SD) are shown. Dot plots are displayed as Superplots, using a color code for the different replicates (Lord *et al*, 2020). Normality of datasets was determined by Shapiro–Wilk test. Statistical differences were calculated by unpaired two‐tailed Student's *t*‐test or Mann–Whitney test (between two groups) and by one‐way ANOVA or Kruskal–Wallis test (between multiple groups), applying Tukey's or Dunn's multiple comparisons test, respectively. Statistical significance was annotated as: *P < 0.05; **P < 0.01; ***P < 0.001; ****P < 0.0001, or not significant (n.s.; *P* > 0.05). Dataset EV3 reports the exact *P*‐values for all statistical tests and sample numerosity. Statistical analyses and graphs were produced using GraphPad Prism 10 (GraphPad, San Diego, CA, USA) software.

## Author contributions

**Matteo Burigotto:** Conceptualization; data curation; formal analysis; supervision; validation; investigation; visualization; methodology; writing – original draft; writing – review and editing. **Vincenza Vigorito:** Data curation; formal analysis; validation; investigation; visualization; methodology; writing – original draft; writing – review and editing. **Colin Gliech:** Conceptualization; data curation; formal analysis; investigation; writing – original draft. **Alessia Mattivi:** Formal analysis; validation; investigation. **Sabrina Ghetti:** Methodology. **Alessandra Bisio:** Resources; methodology. **Graziano Lolli:** Conceptualization. **Andrew J Holland:** Conceptualization; resources; supervision; writing – original draft. **Luca L Fava:** Conceptualization; resources; data curation; formal analysis; supervision; funding acquisition; visualization; methodology; writing – original draft; project administration; writing – review and editing.

## Disclosure and competing interests statement

The authors declare that they have no conflict of interest.

## Supporting information



Appendix

Expanded View Figures PDF

PDF+

Movie EV1

Dataset EV1

Dataset EV2

Dataset EV3

Source Data for Expanded View

Source Data for Figure 1

Source Data for Figure 2

Source Data for Figure 3

Source Data for Figure 4

## Data Availability

No large primary datasets have been generated and deposited. Source data can be found either linked to the respective figures or at the BioStudies archive (accession number: S‐BSST1173 (https://www.ebi.ac.uk/biostudies/studies/S‐BSST1173)).
