# Supplementary material for: PLK1 promotes the mitotic surveillance pathway by controlling cytosolic 53BP1 availability
Source: EMBO Rep. 2023 Oct 27;24(12):e57234. doi: 10.15252/embr.202357234 (PMC10702821; doi:10.15252/embr.202357234)
Supplement: Supplementary file 1 — Appendix [file EMBR-24-e57234-s001.pdf]

APPENDIX

PLK1 promotes the mitotic surveillance pathway by controlling cytosolic 53BP1 availability

Matteo Burigotto, Vincenza Vigorito, Colin Gliech, Alessia Mattivi, Sabrina Ghetti, Alessandra Bisio, Graziano Lolli, Andrew J. Holland and Luca L. Fava

Table of Contents

Appendix Figure S1 – page 2
Appendix Figure S2 – page 4
Appendix Figure S3 – page 5
Appendix Figure S4 – page 6
Appendix Figure S5 – page 8
Appendix Figure S6 – page 9
Appendix Figure S7 – page 10
Appendix Figure S8 – page 11
Appendix Figure S9 – page 12

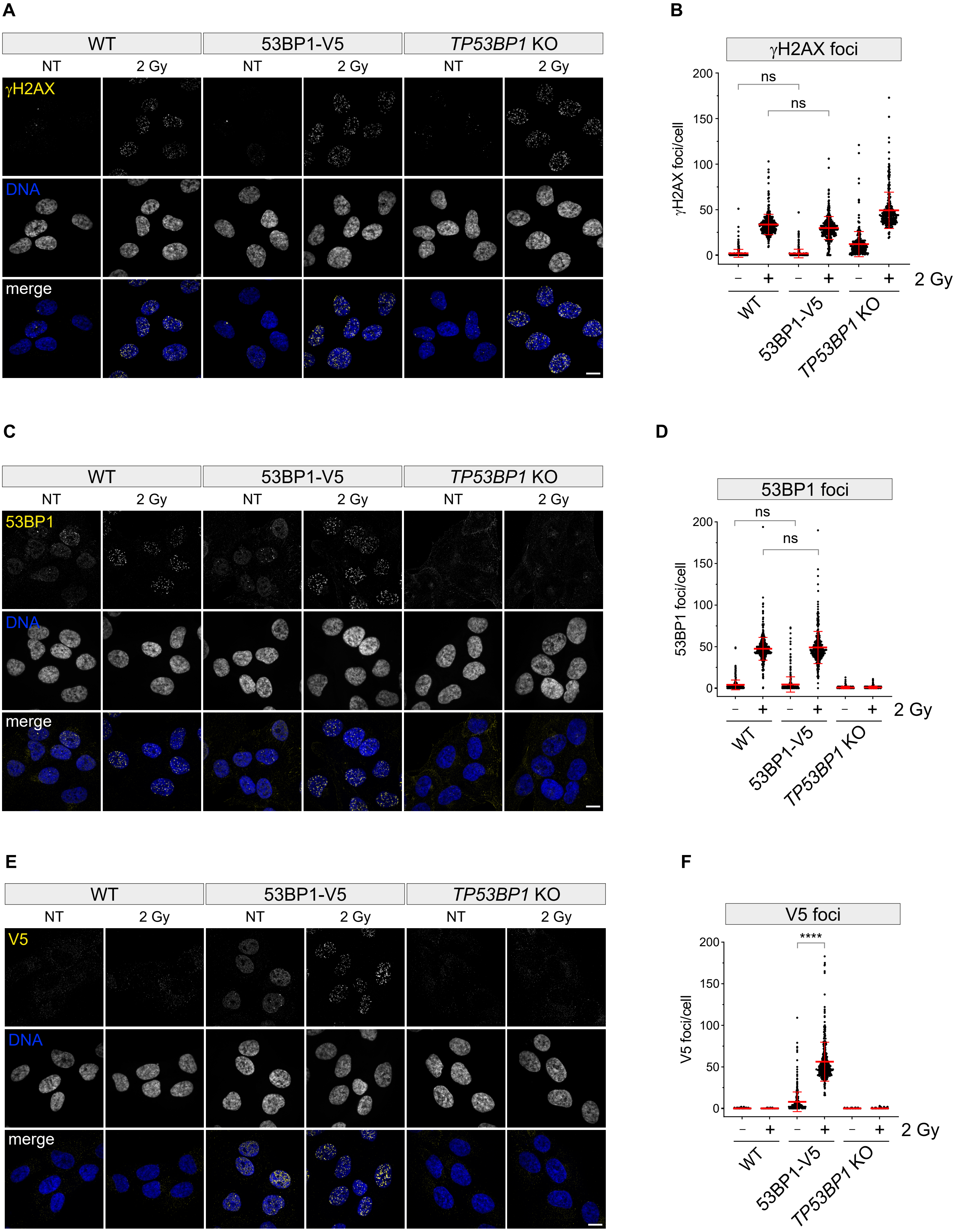
A
WT
53BP1-V5
TP53BP1 KO
NT
2 Gy
γH2AX
DNA
merge
B
γH2AX foci
γH2AX foci/cell
200
150
100
50
0
ns
ns
−
+
2 Gy
WT
53BP1-V5
TP53BP1 KO
C
WT
53BP1-V5
TP53BP1 KO
NT
2 Gy
53BP1
DNA
merge
D
53BP1 foci
53BP1 foci/cell
200
150
100
50
0
ns
ns
−
+
2 Gy
WT
53BP1-V5
TP53BP1 KO
E
WT
53BP1-V5
TP53BP1 KO
NT
2 Gy
V5
DNA
merge
F
V5 foci
V5 foci/cell
200
150
100
50
0
****
−
+
2 Gy
WT
53BP1-V5
TP53BP1 KO

Appendix Figure S1.

(A) Representative fluorescence micrographs of cells subjected to irradiation (2 Gy) or left untreated and probed against γH2AX protein. Scale bar: 10 μm. **(B)** Dot plots showing the number of γH2AX foci in cells treated as in A). Mean values (red lines) ± SD are reported. N ≥ 280 cells were quantified for each condition. Kruskal-Wallis test: n.s. = non-significant. **(C)** Representative fluorescence micrographs of cells subjected to irradiation (2 Gy) or left untreated and probed against 53BP1 protein. Scale bar: 10 μm. **(D)** Dot plots showing the number of 53BP1 foci in cells treated as in A). Mean values (red lines) ± SD are reported. N ≥ 352 cells were quantified for each condition. Kruskal-Wallis test: n.s. = non-significant. **(E)** Representative fluorescence micrographs of cells treated as in A) and probed against V5-tag. Scale bar: 10 μm **(F)** Dot plots showing the number of V5 foci in cells treated as in A). Mean values (red lines) ± SD are reported. N ≥ 349 cells were quantified for each condition. Kruskal-Wallis test: ****P < 0.0001.

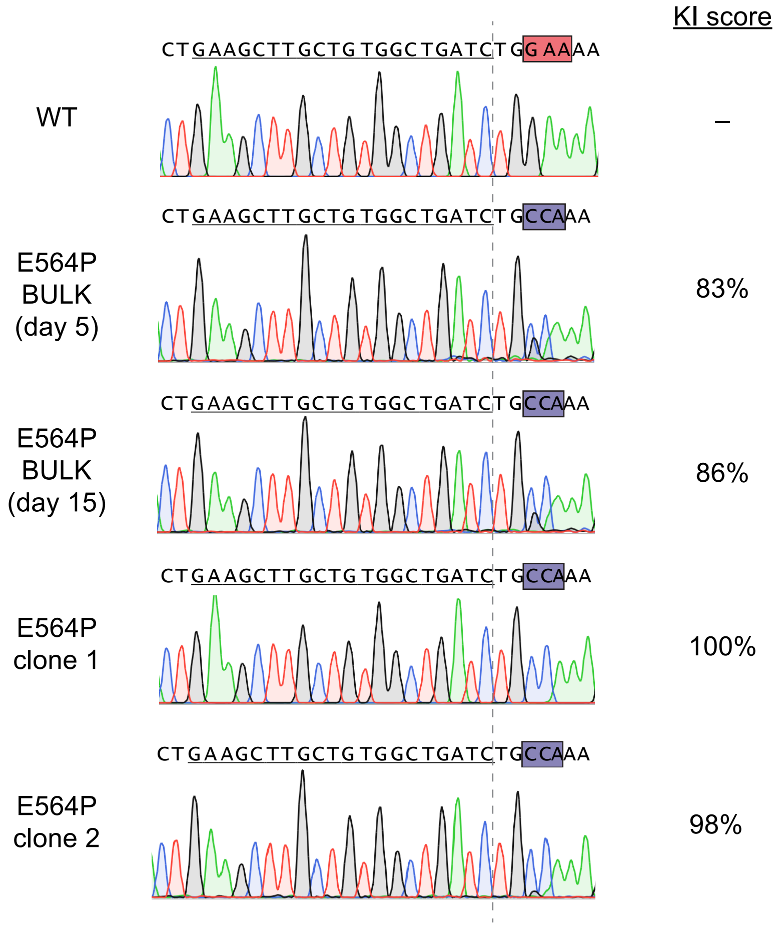

Appendix Figure S2.
The *CENPF* region targeted by the gRNA (black solid line) was PCR amplified and sequenced from genomic DNA of the indicated cells. Electropherograms are shown, along with the knock-in score calculated by ICE. Vertical dashed line: Cas9 cut site; red box: WT codon (E564); blue box: mutant codon (P564).

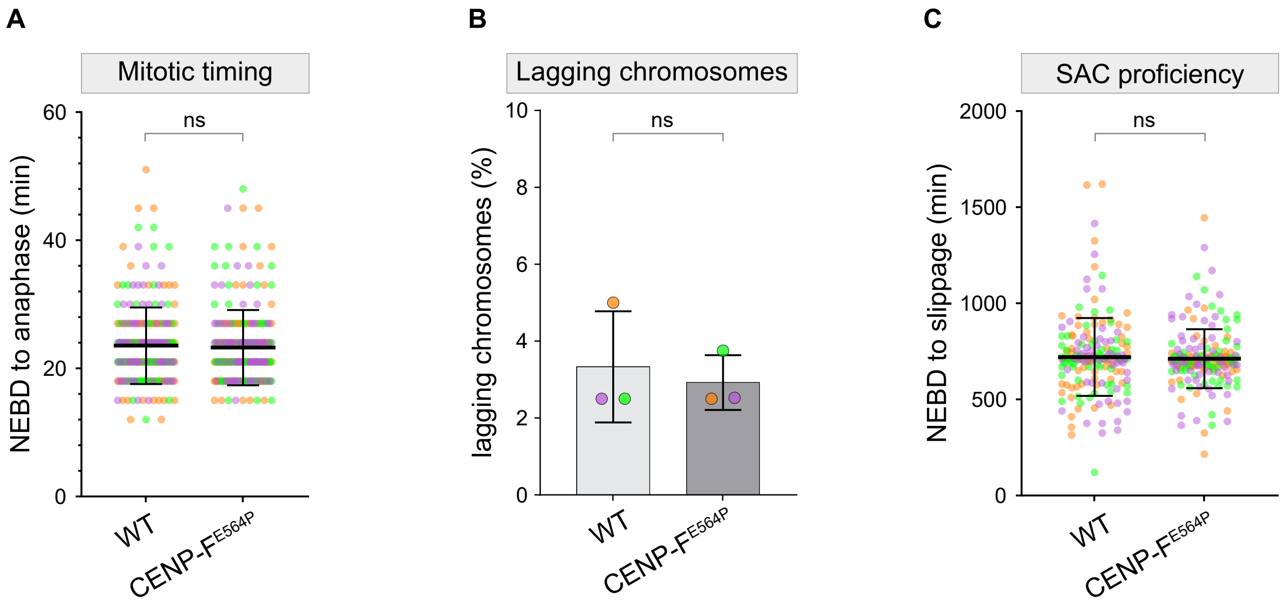

Appendix Figure S3.
(A) Asynchronously growing RPE1 cells of the indicated genotypes were filmed as they progressed through mitosis. Mitotic duration was measured as the time elapsed from nuclear envelope breakdown (NEBD) to anaphase onset. Each dot represents a single cell; mean values (black lines) ± SD (calculated on the entire dataset) are reported. N = 3 biological replicates are shown, one replicate in green, one in magenta, one in orange. Significance was tested using a Mann-Whitney test: n.s. = non-significant. The total number of cells displayed is reported in the second tab of Dataset EV3. **(B)** The percentage of lagging chromosomes was assessed from cells filmed as in A). Mean values (black lines) ± SD are reported. N = 3 biological replicates. Mann-Whitney test: n.s. = non-significant. **(C)** To assess spindle assembly checkpoint (SAC) proficiency, RPE1 cells of the indicated genotype were pre-treated with 1 h with nocodazole and then imaged (in the presence of nocodazole) for 48 h. The dot plots show the time between NEBD and mitotic slippage for each cell. Mean values (black lines) ± SD (calculated on the entire dataset) are reported. N = 3 biological replicates are shown, one replicate in green, one in magenta, one in orange. Significance was tested using a Mann-Whitney test: n.s. = non-significant. The total number of cells displayed is reported in the second tab of Dataset EV3.

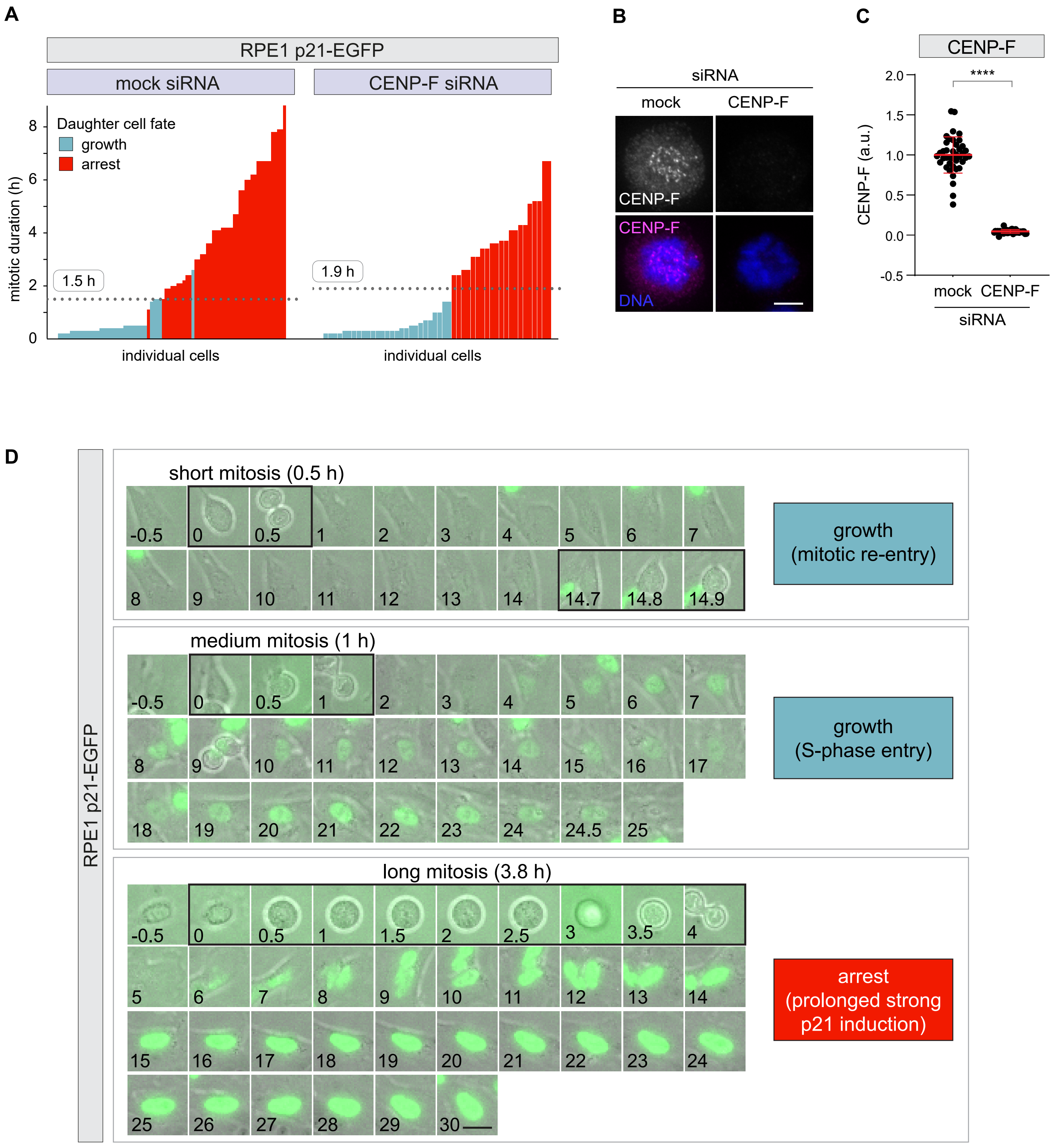
A
RPE1 p21-EGFP
mock siRNA
CENP-F siRNA
mitotic duration (h)
Daughter cell fate
growth
arrest
1.5 h
1.9 h
individual cells
individual cells
B
siRNA
mock
CENP-F
CENP-F
CENP-F
DNA
C
CENP-F
CENP-F (a.u.)
****
mock
CENP-F
siRNA
D
RPE1 p21-EGFP
short mitosis (0.5 h)
growth
(mitotic re-entry)
medium mitosis (1 h)
growth
(S-phase entry)
long mitosis (3.8 h)
arrest
(prolonged strong
p21 induction)

Appendix Figure S4.

(A) RPE1 p21-EGFP cells were transfected with the indicated siRNA, transiently treated with dimethylenastron and imaged for 2 days. The graph shows the daughter cell fate in relation to the time spent in mitosis by the mother cell. Each daughter cell is represented by a vertical bar, whose height represents the mitotic duration of its mother and the colour indicating the fate of the daughter (growth or arrest). The dotted lines show the MSP threshold. N = 77 control cells and 48 CENP-F depleted cells. **(B)** Representative images of RPE1 p21-EGFP cells transfected with the indicated siRNA and subjected to immunofluorescence using the indicated antibodies. Scale bar: 5 µm. **(C)** Dot plots showing the nuclear CENP-F intensity from images obtained as in B). Mean values (red lines) ± SD are reported, normalized on the mock sample; a.u. = arbitrary units. N = 40 cells per condition. Unpaired t-test (****P < 0.0001). **(D)** Movie stills of representative RPE1 p21-EGFP cells subjected to the timer assay as described in Fig. 2F. Mitotic timing was measured as the time elapsed between cell rounding and anaphase onset, based on the phase contrast signal (grey). Daughter cell fate was assessed as “growth” based on mitotic re-entry (upper panel) or abrupt decease in p21 signal (green), indicative of S-phase entry (middle panel), and as “arrest” in the presence of a prolonged strong p21 induction (lower panel). Time is expressed in hours; scale bar: 20 µm.

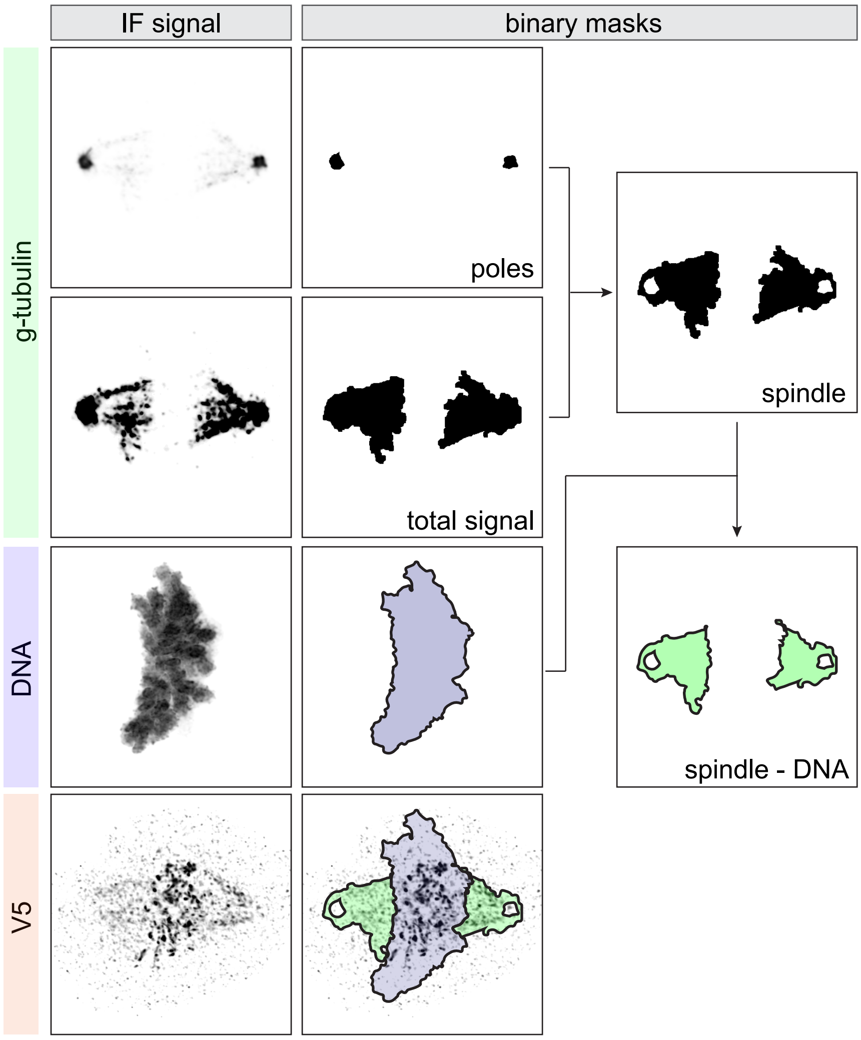

Appendix Figure S5.
Strategy to quantify 53BP1 immunofluorescence (IF) signal in the stripping assay from images as in EV3A. A mitotic spindle binary mask (shown in green) was obtained subtracting a mask for spindle poles (shown as black dots) and a DNA mask (shown in blue) to the total γ-tubulin signal. The resulting mask was used to measure 53BP1 fluorescence intensity in a region of the spindle devoid of DNA and not comprising the two centrosomes.

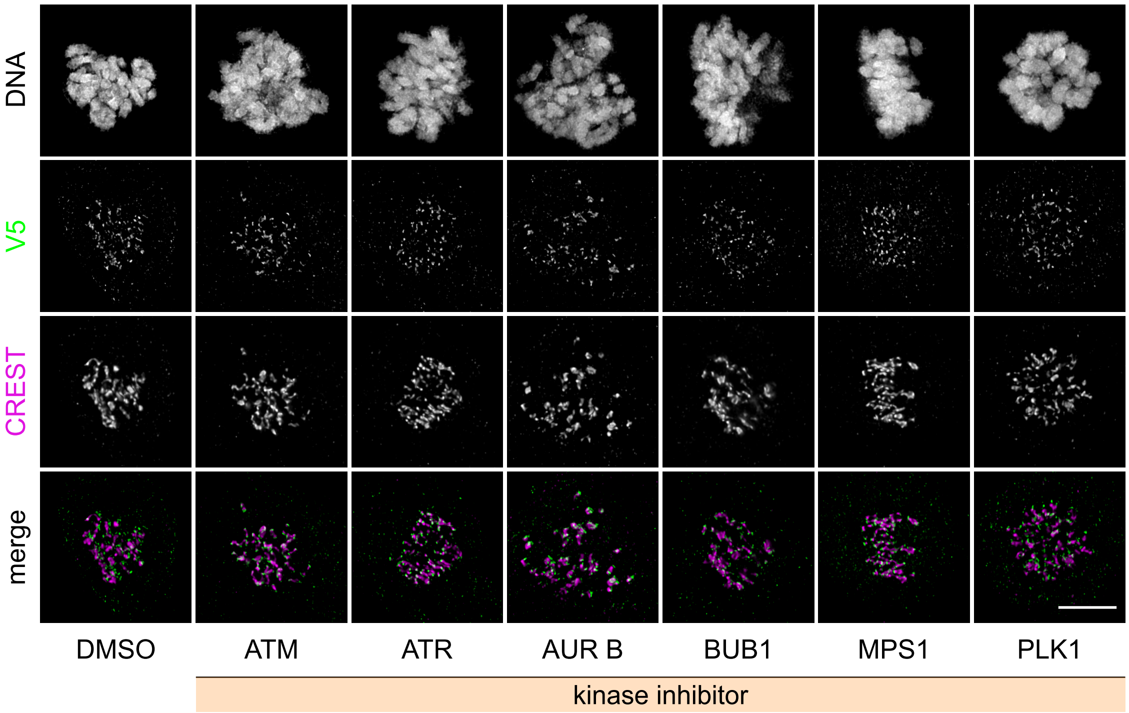

Appendix Figure S6.
Representative fluorescence micrographs of RPE1 53BP1-V5 cells treated as in Fig. 3A and co-stained with the indicated antibodies. Async = asynchronous cells; AUR B = Aurora B. Scale bar: 5 μm.

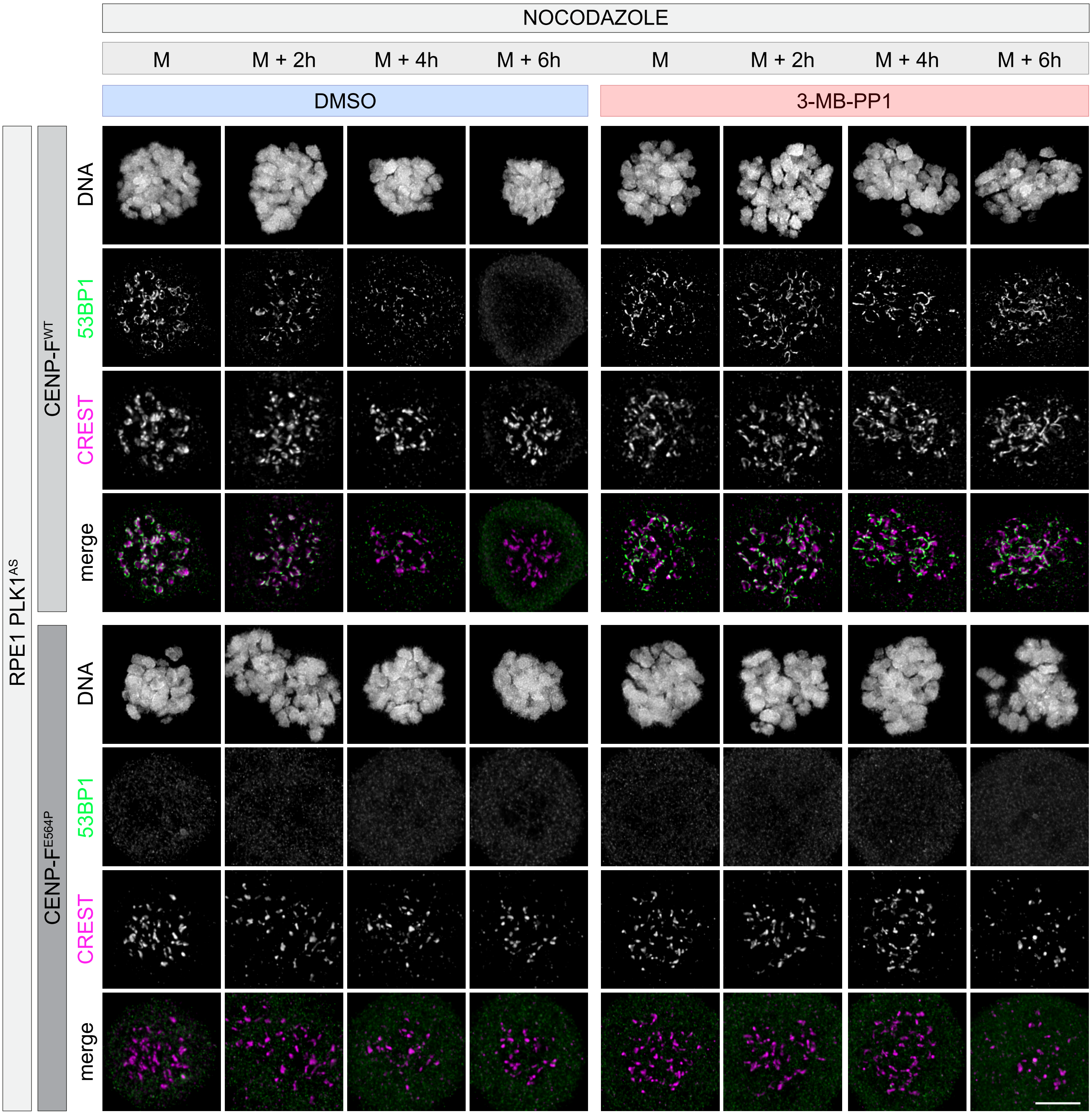

Appendix Figure S7
Representative fluorescence micrographs of RPE1 cells of the indicated genotype treated as in Fig. 3C. M = mitosis. Scale bar: 5 µm.

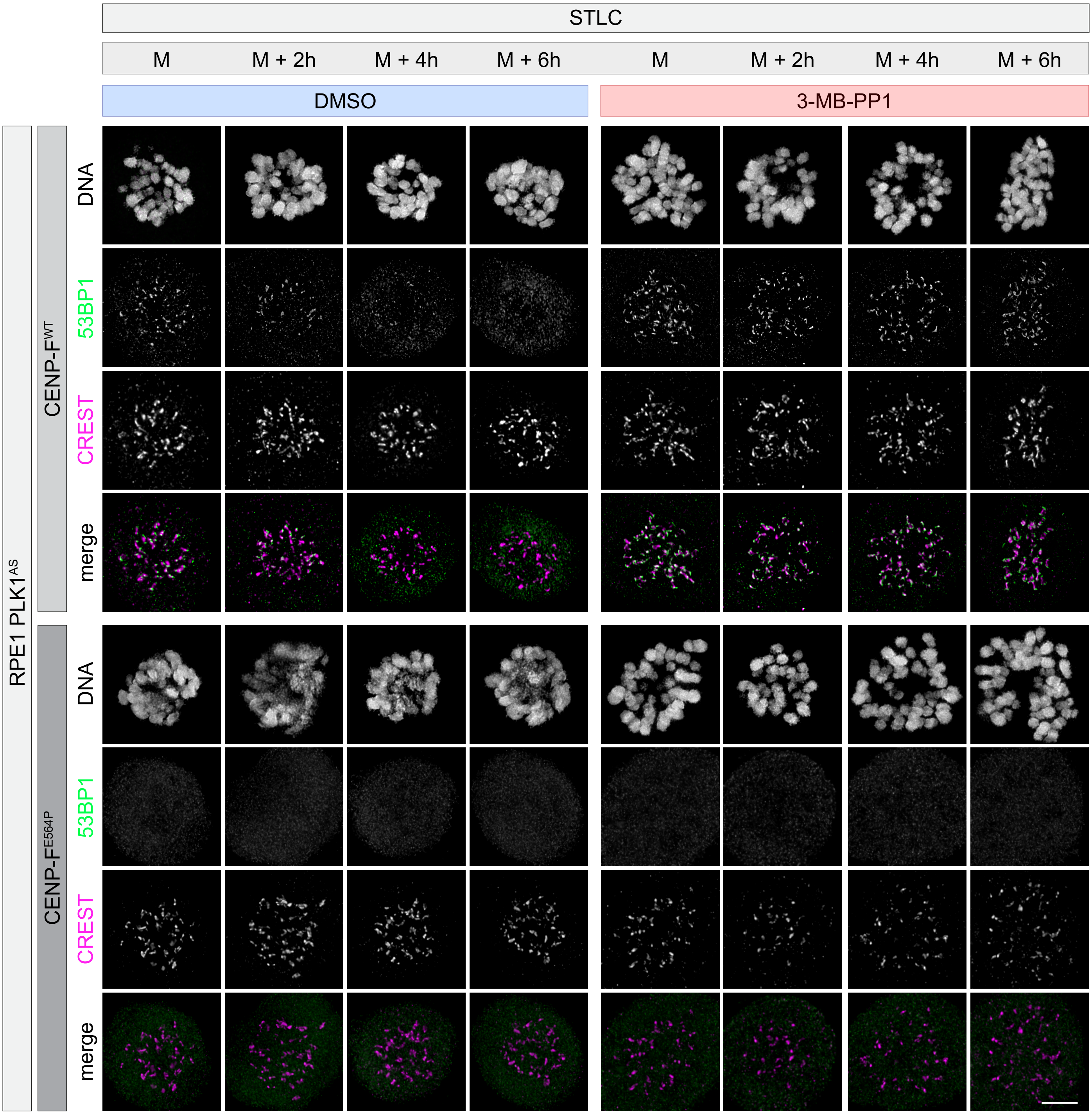

Appendix Figure S8
Representative fluorescence micrographs of RPE1 cells of the indicated genotype treated as in Fig. EV4B. M = mitosis. Scale bar: 5 µm.

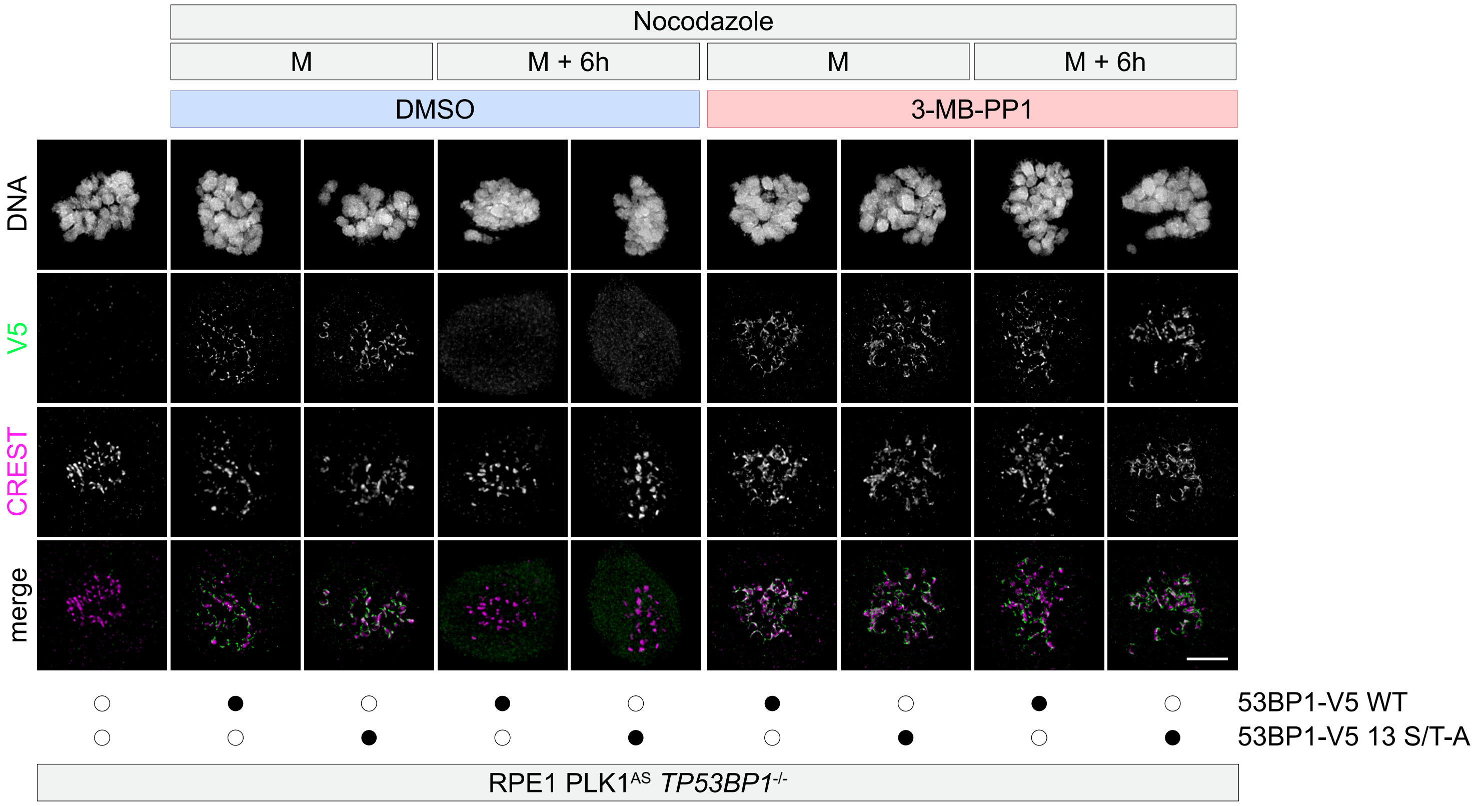

Appendix Figure S9
Representative fluorescence micrographs of RPE1 PLK1[AS] *TP53BP1* KO cells treated as in Fig. EV5A. M = mitosis. Scale bar: 5 µm.
