# Supplementary material for: PLK1 promotes the mitotic surveillance pathway by controlling cytosolic 53BP1 availability
Source: EMBO Rep. 2023 Oct 27;24(12):e57234. doi: 10.15252/embr.202357234 (PMC10702821; doi:10.15252/embr.202357234)
Supplement: Supplementary file 2 — Expanded View Figures PDF [file EMBR-24-e57234-s011.pdf]

Expanded View Figures

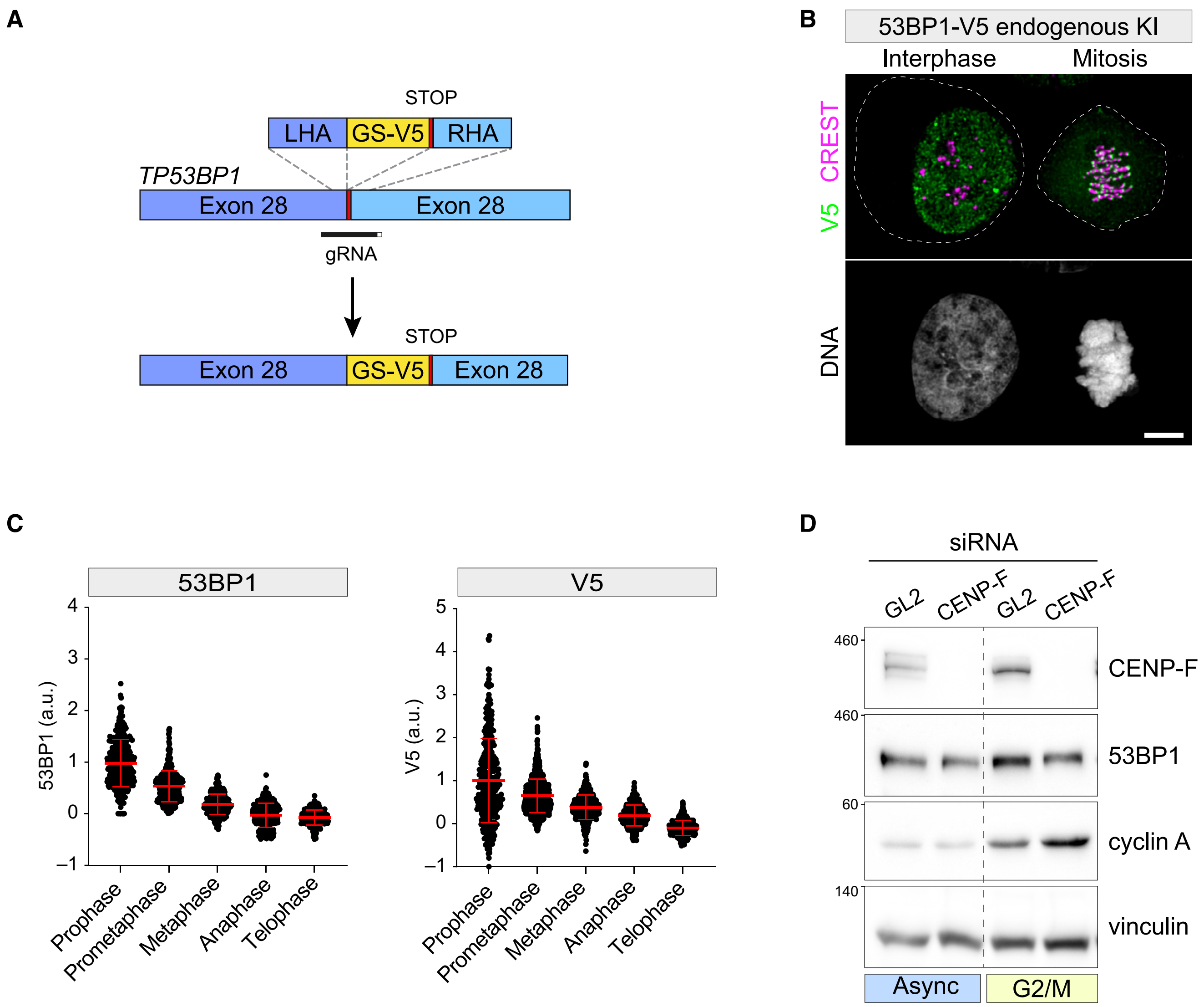

Figure EV1. 53BP1-V5 endogenous knock-in does not perturb 53BP1 localization and dynamics.

A Schematic of the knock-in strategy to introduce the V5 sequence into the endogenous *TP53BP1* locus. LHA: left homology arm; RHA: right homology arm; GS-V5: Gly-Ser linker followed by V5-epitope tag.

B Representative fluorescence micrograph of 53BP1-V5 cells co-stained with the indicated antibodies. An interphase (left) and a mitotic cell (right) are shown. The dashed lines indicate the plasma membrane of the two cells. Scale bar: 5 μm.

C Dot plots showing 53BP1 (in RPE1 WT cells, left panel) or V5 (in RPE1 53BP1-V5 cells, right panel) fluorescence intensity at individual KTs across the indicated cell cycle phases. Mean values (red lines) ± SD are reported, normalized on the prophase sample. $N \geq 349$ KTs were assessed from 10 cells for each mitotic phase; a.u. = arbitrary units.

D HeLa S3 cells were transfected with the indicated siRNA and either treated with thymidine for 24 h and released in fresh medium for 10 h (G_2/M), or left untreated (async = asynchronous). Cells were subjected to immunoblotting using the indicated antibodies.

Source data are available online for this figure.

 © 2023 The Authors

Figure EV2. Targeted knock-in strategy to interfere with 53BP1 localization at kinetochores.

A Chronos gene dependency score of CENPF across 1,078 cell lines from DepMap database (CRISPR DepMap 22Q4). *x*-axis: Chronos score.

B Schematic of the CENP-F prey fragments retrieved by the yeast two-hybrid screen and their relative position to the CENP-F sequence. The overlap between all the different clones identifies a region of 25 amino acids as putative binding domain between the KT-binding domain of 53BP1 (bait) and CENP-F.

C AlphaFold modeling of the putative 53BP1 binding domain of CENP-F. Introduction of a proline in position 564 is predicted to introduce a kink in this coiled coil region.

D Schematic depicting the knock-in strategy used to introduce E564P mutation in CENPF. The gRNA recognition site, PAM sequence and cut site (dashed vertical line) are presented.

E Representative fluorescence micrographs of RPE1 cells of the indicated genotype, co-stained with the indicated antibodies. Scale bar: 5 μm.

F Dot plots showing the intensity of NudE protein at individual KTs from images as in (E). Each dot represents a particular KT. Mean values (black lines) ± SD (calculated on the entire dataset) are reported, normalized on the WT sample; a.u. = arbitrary units. $N = 2$ biological replicates are shown, one replicate in green, one in magenta. Significance was tested using a Kruskal–Wallis test: n.s. = non-significant. The total number of KTs displayed is reported in the second tab of Dataset EV3.

Source data are available online for this figure.

© 2023 The Authors

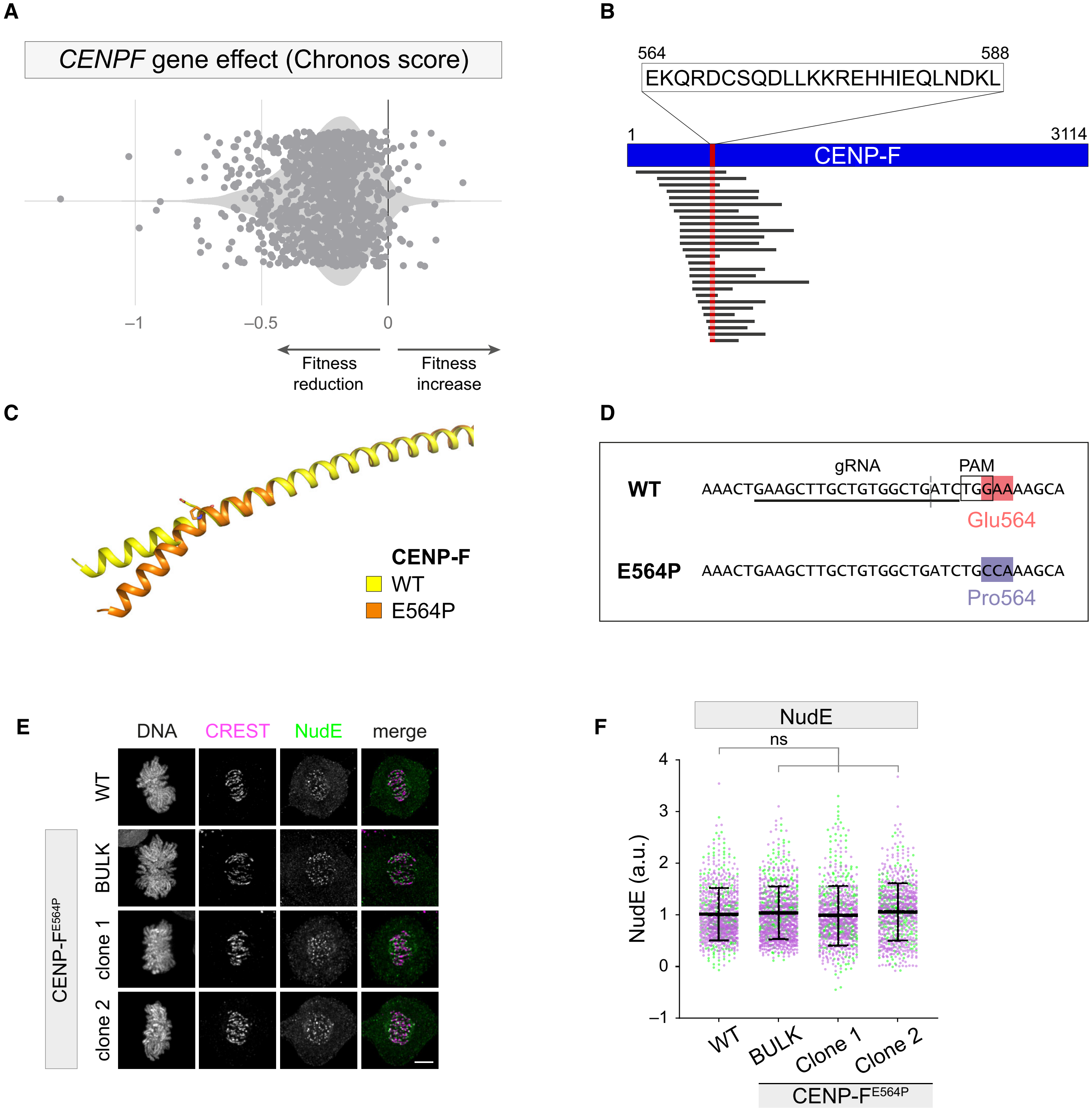

Figure EV2.

© 2023 The Authors

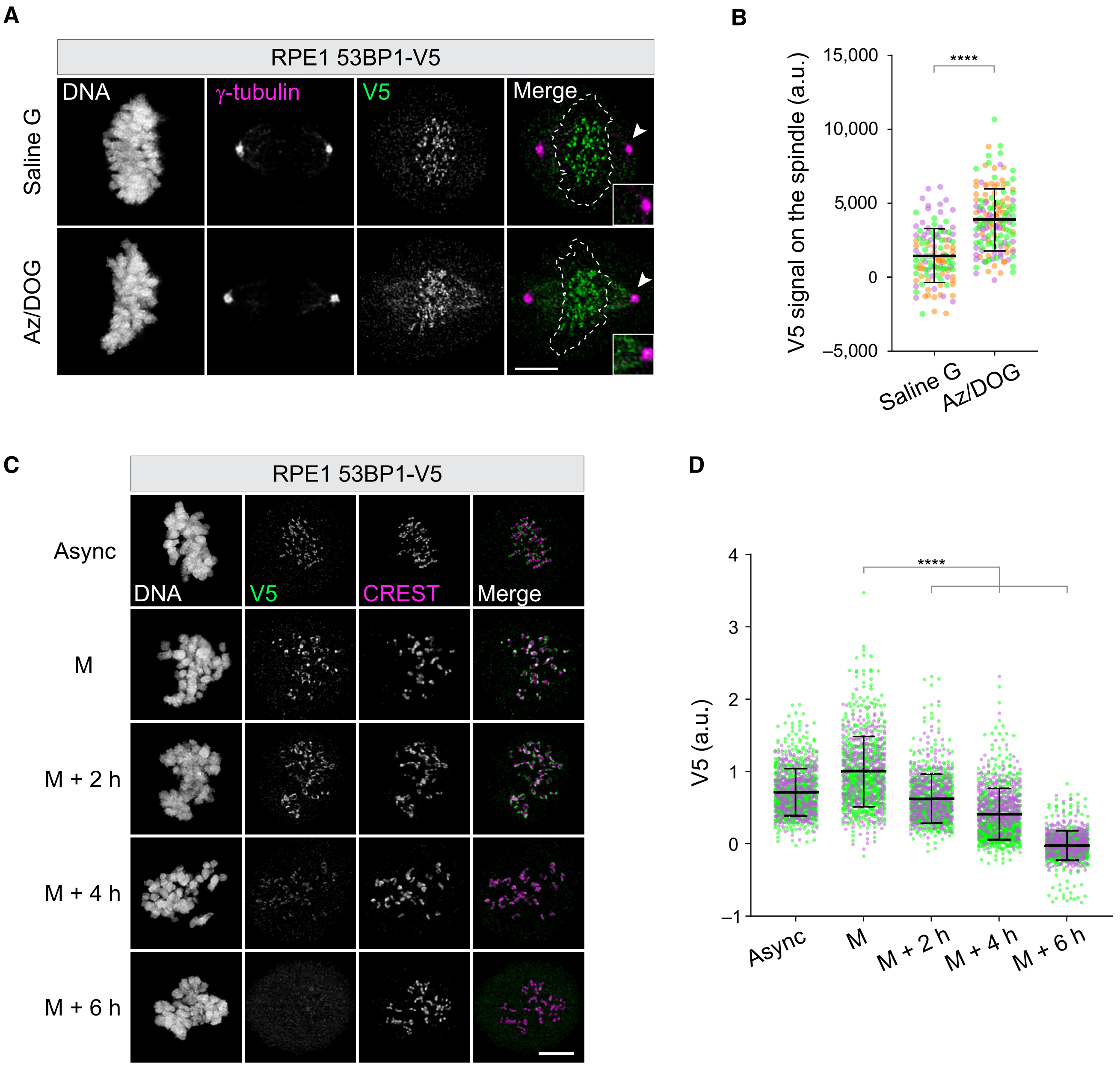

Figure EV3. 53BP1 is removed from kinetochores by stripping and a time-dependent loss-of-affinity.

A Representative fluorescence micrographs of RPE1 53BP1-V5 cells incubated for 15 min in isotonic salt solution in the presence of either glucose (Saline G) or sodium azide/2-deoxy-D-glucose (AZ/DOG) and co-stained with the indicated antibodies. The blow-up shows a portion of the mitotic spindle in proximity to one of the spindle poles (arrowhead). Scale bar: 5 μm.

B Dot plots showing V5 fluorescence intensity on the spindle obtained from images as in (A). Each dot represents a particular spindle. Mean values (black lines) ± SD (calculated on the entire dataset) are reported; a.u. = arbitrary units. $N = 3$ biological replicates are shown, one replicate in green, one in magenta, one in orange. Significance was tested using an unpaired *t*-test (****$P < 0.0001$). The number of spindles measured is reported in the second tab of Dataset EV3.

C RPE1 53BP1-V5 cells were either left untreated (async = asynchronous) or synchronized in prometaphase in medium containing nocodazole, fixed immediately (M = mitosis) or after 2, 4, or 6 h and co-stained with the indicated antibodies. Scale bar: 5 μm.

D Dot plots showing V5 fluorescence intensity at individual KTs from images as in (C). Each dot represents a particular KT. Mean values (black lines) ± SD (calculated on the entire dataset) are reported, normalized on the mitotic (M) sample; a.u. = arbitrary units. $N = 2$ biological replicates are shown, one replicate in green, one in magenta. Significance was tested using a Kruskal–Wallis test (****$P < 0.0001$). The total number of KTs displayed is reported in the second tab of Dataset EV3.

Source data are available online for this figure.

Figure EV4. PLK1 control of 53BP1 kinetochore dynamics is independent of microtubule status.

A Representative fluorescence micrographs of RPE1 53BP1-V5 cells treated as in Fig 3B and co-stained with the indicated antibodies. Async = asynchronous cells; AUR B = Aurora B. Scale bar: 5 μm.

B Cells of the indicated genotypes were synchronized in prometaphase in medium containing STLC, in the presence or absence of PLK1 inhibition (3-MB-PP1) and fixed immediately (M = mitosis) or after 2, 4, or 6 h. Dot plots show 53BP1 fluorescence intensity at individual KTs. Each dot represents a particular KT. Mean values (black lines) ± SD (calculated on the entire dataset) are reported, normalized on the WT early mitotic sample; a.u. = arbitrary units. *N* = 2 biological replicates are shown, one replicate in blue, one in yellow. Significance was tested using a Kruskal-Wallis test: *****P* < 0.0001; n.s. = non-significant. The total number of KTs displayed is reported in the second tab of Dataset EV3.

Source data are available online for this figure.

 © 2023 The Authors

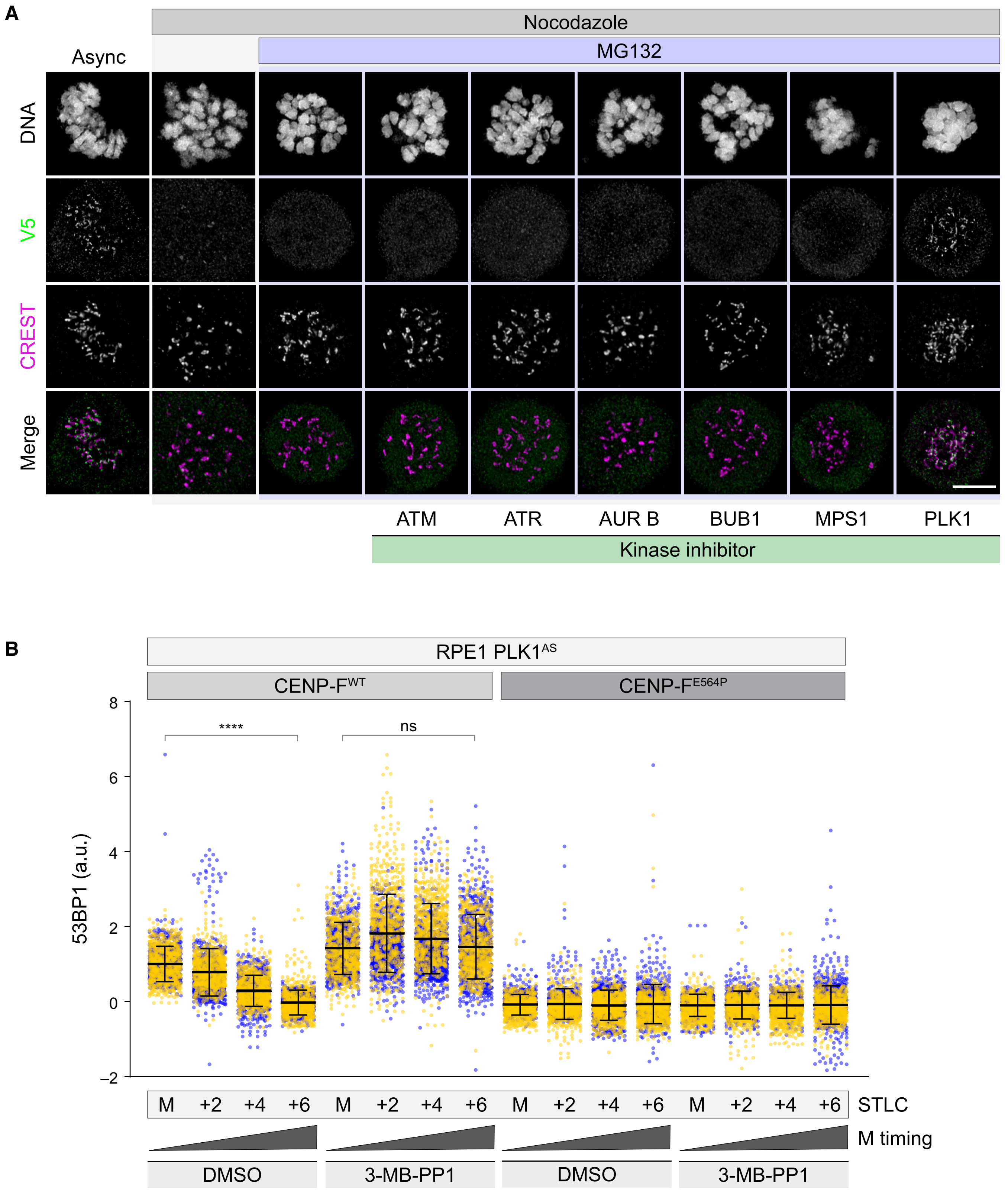
A
Nocodazole
MG132
Async
DNA
V5
CREST
Merge
ATM
ATR
AUR B
BUB1
MPS1
PLK1
Kinase inhibitor
B
RPE1 PLK1AS
CENP-FWT
CENP-FE564P
****
ns
53BP1 (a.u.)
8
6
4
2
0
−2
M +2 +4 +6 M +2 +4 +6 M +2 +4 +6 M +2 +4 +6
STLC
M timing
DMSO
3-MB-PP1
DMSO
3-MB-PP1

Figure EV4.

Figure EV5. A 53BP1 phosphomutant displays intact PLK1-dependent kinetochore dynamics.

A RPE1 PLK1^{AS} *TP53BP1* KO cells were transduced with the indicated lentiviral vectors, synchronized in prometaphase in the presence or absence of PLK1 inhibition (3-MB-PP1) and fixed either immediately (M = mitosis) or after 6 h (M + 6 h). Dot plots show V5 fluorescence intensity at individual KTs. Each dot represents a particular KT. Mean values (black lines) ± SD (calculated on the entire dataset) are reported, normalized on the 53BP1 WT-expressing sample at the earliest timepoint; a.u. = arbitrary units. *N* = 2 biological replicates are shown, one replicate in green, one in magenta. Significance was tested using a Kruskal-Wallis test: n.s. = non-significant. The total number of KTs displayed is reported in the second tab of Dataset EV3.

B Representative fluorescence micrographs of RPE1 cells of the indicated genotype, treated as in Fig 4A. M = mitosis. Scale bar: 5 µm.

Source data are available online for this figure.

 © 2023 The Authors

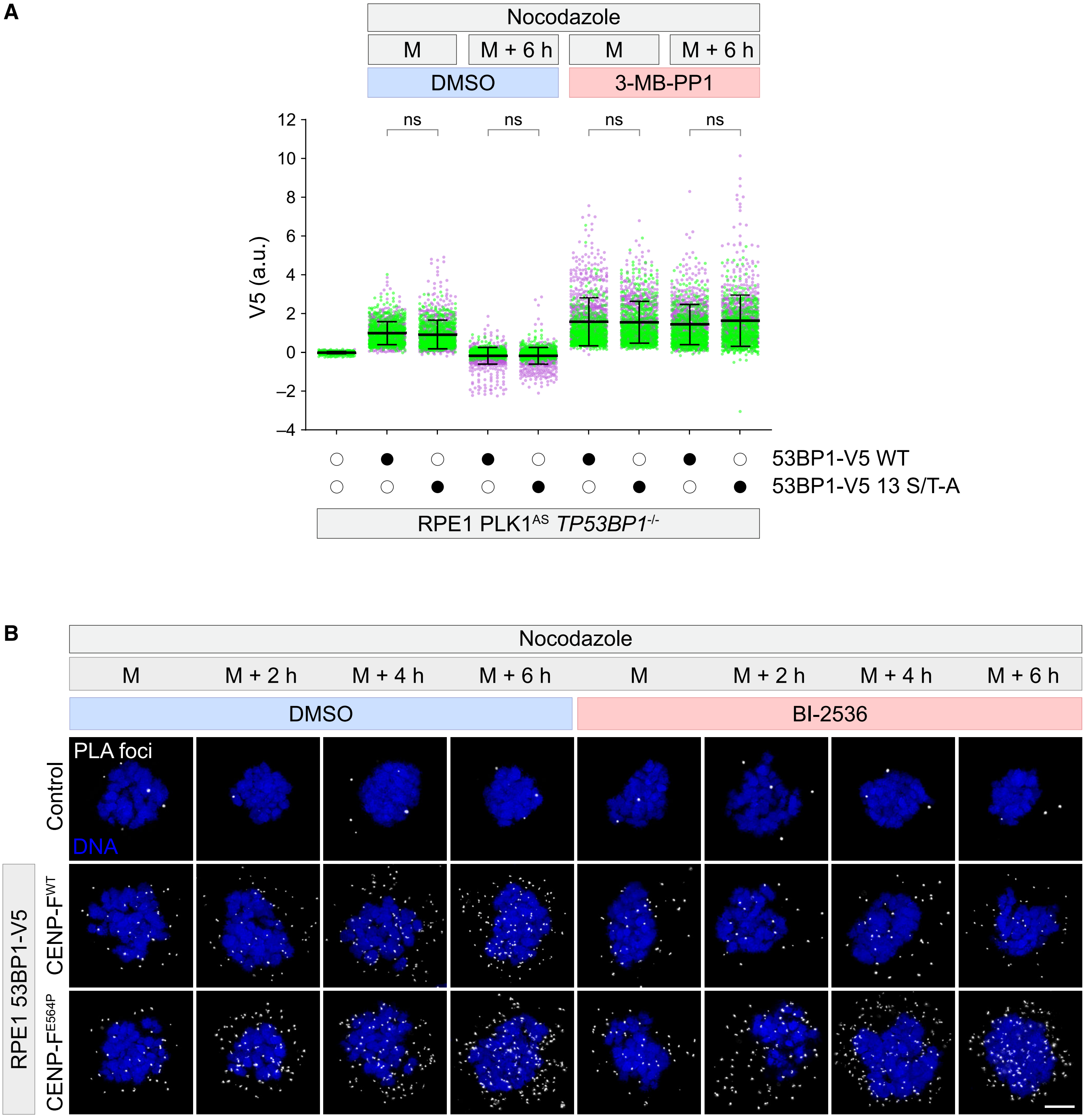
A
Nocodazole
M
M + 6 h
M
M + 6 h
DMSO
3-MB-PP1
V5 (a.u.)
12
10
8
6
4
2
0
−2
−4
ns
ns
ns
ns
53BP1-V5 WT
53BP1-V5 13 S/T-A
RPE1 PLK1AS TP53BP1-/-
B
Nocodazole
M
M + 2 h
M + 4 h
M + 6 h
M
M + 2 h
M + 4 h
M + 6 h
DMSO
BI-2536
PLA foci
DNA
Control
CENP-FWT
CENP-FE564P
RPE1 53BP1-V5

Figure EV5.
