# Supplementary figures and images for: PLK1 promotes the mitotic surveillance pathway by controlling cytosolic 53BP1 availability
Source: EMBO Rep. 2023 Oct 27;24(12):e57234. doi: 10.15252/embr.202357234 (PMC10702821; doi:10.15252/embr.202357234)

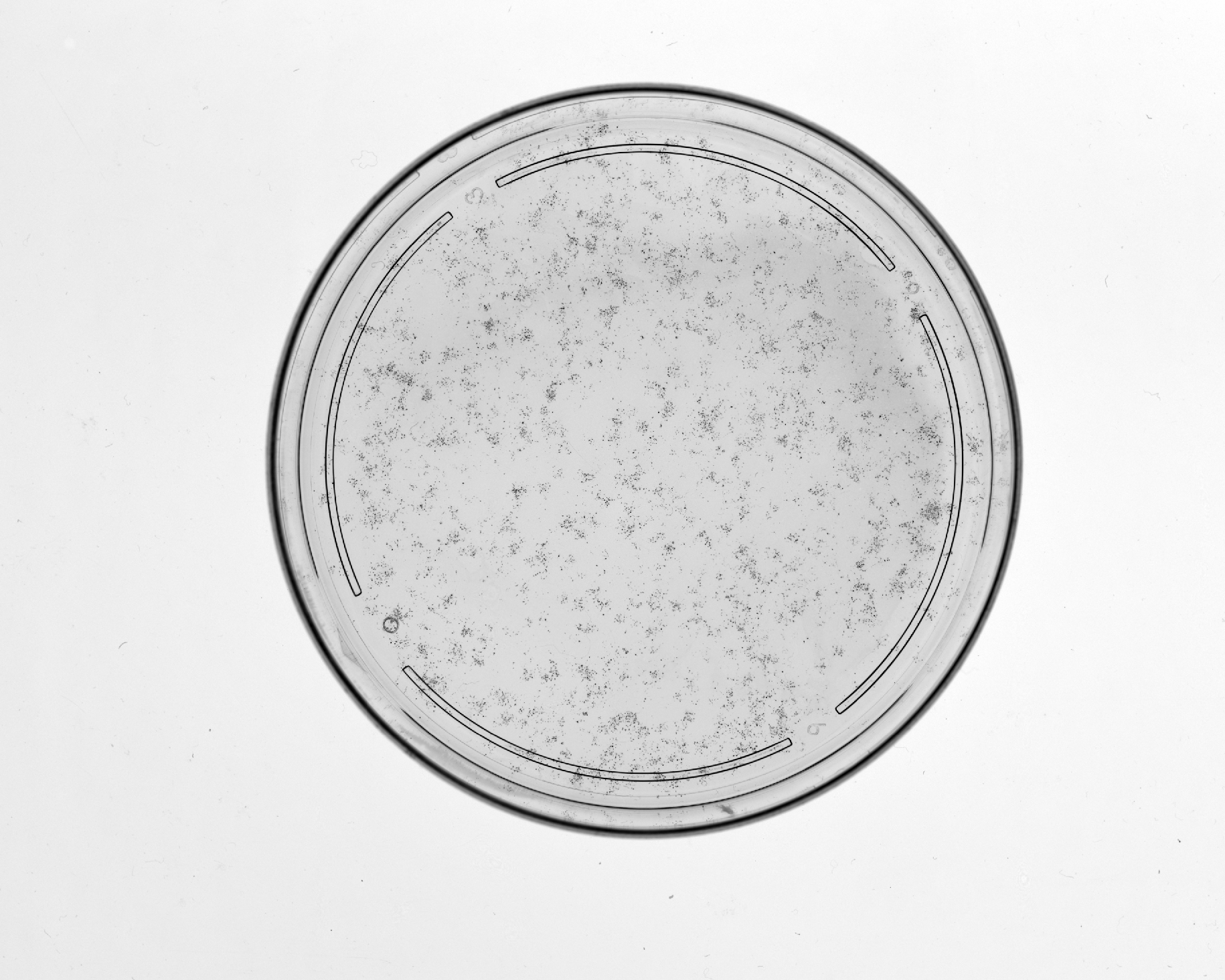

Supplement: Supplementary file 10 — Source Data for Figure 2 [file EMBR-24-e57234-s009.zip › Figure 2/2A/E564P BULK centrinone.tif]

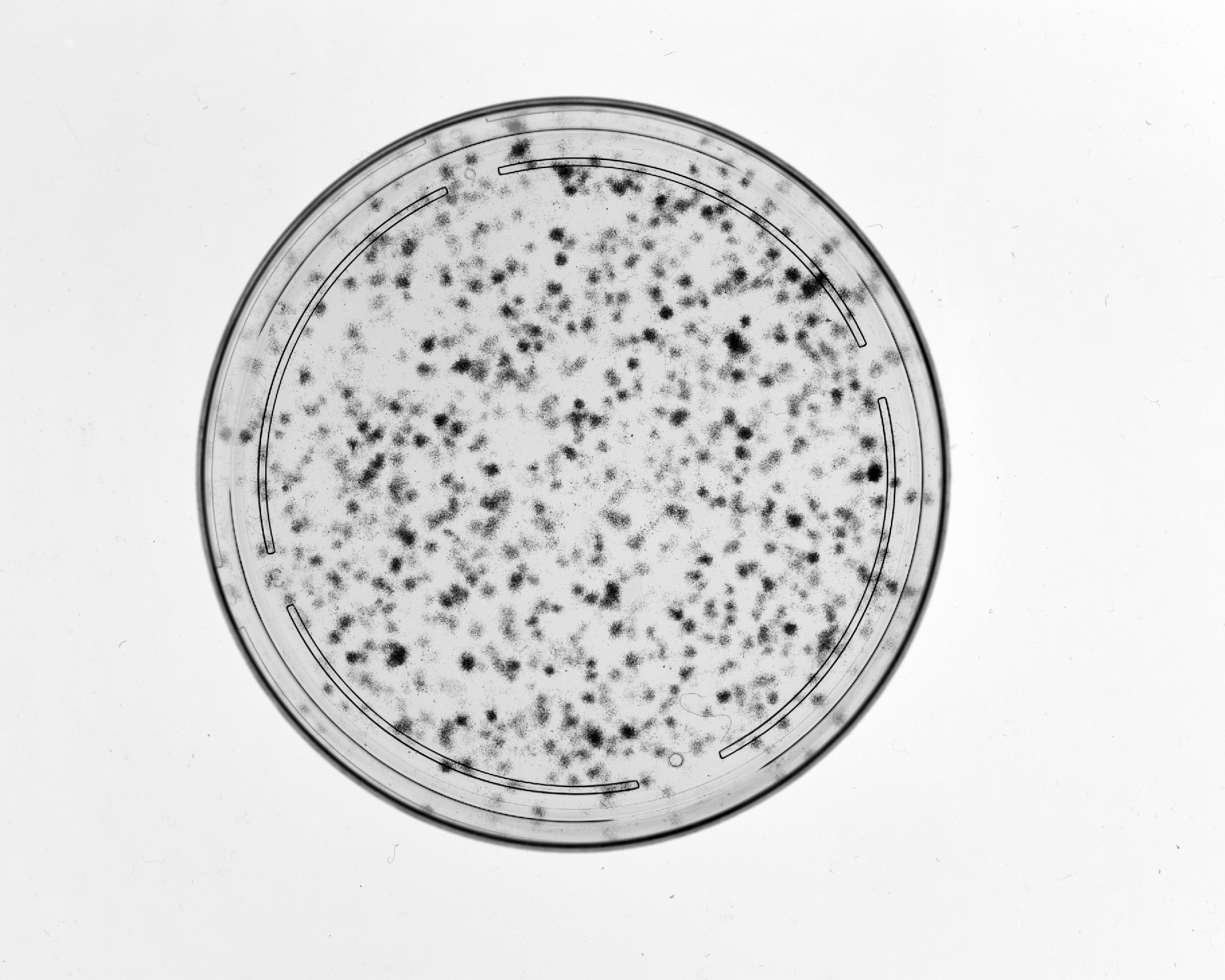

Supplement: Supplementary file 10 — Source Data for Figure 2 [file EMBR-24-e57234-s009.zip › Figure 2/2A/E564P BULK NT #1.tif]

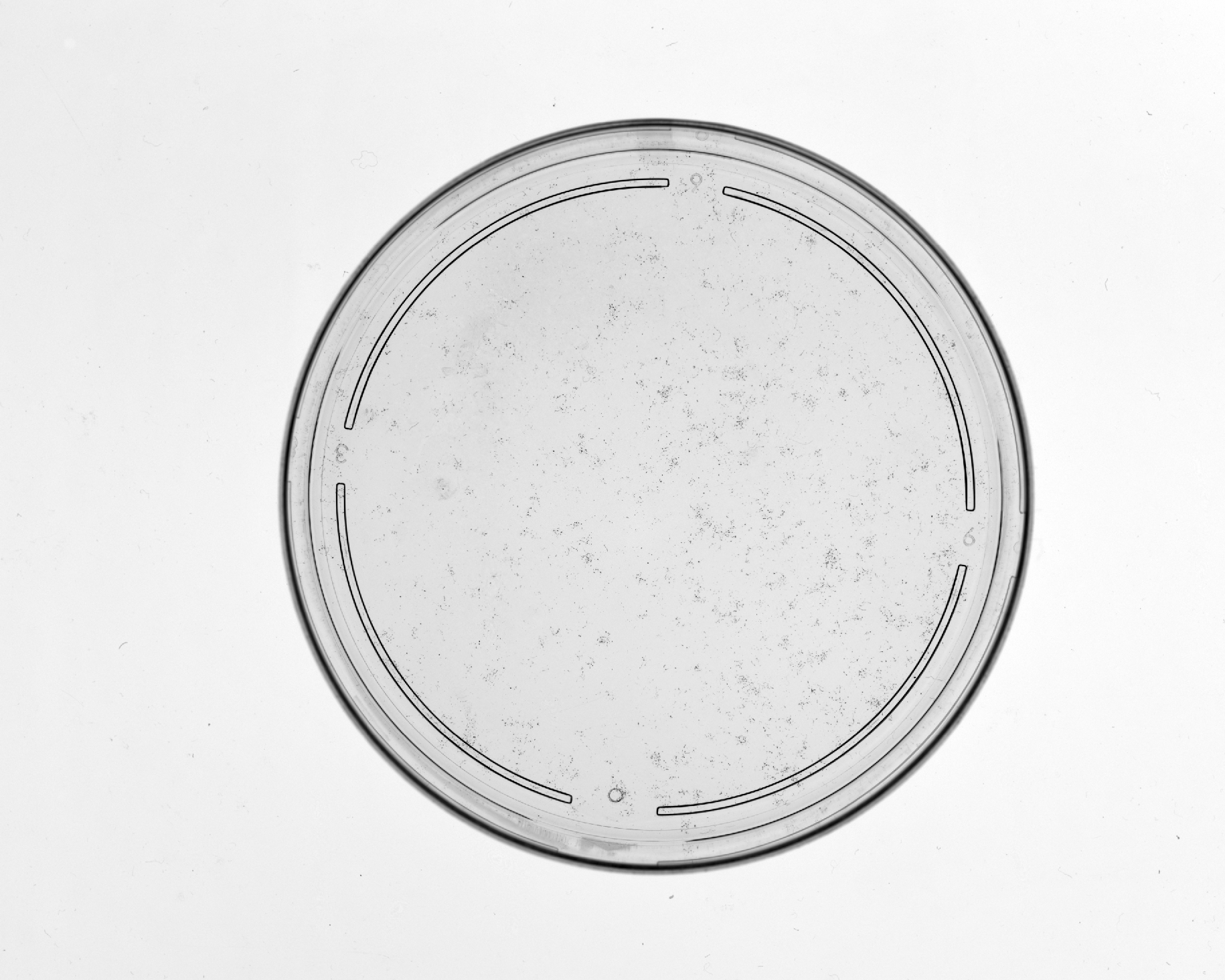

Supplement: Supplementary file 10 — Source Data for Figure 2 [file EMBR-24-e57234-s009.zip › Figure 2/2A/E564P clone 1 centrinone.tif]

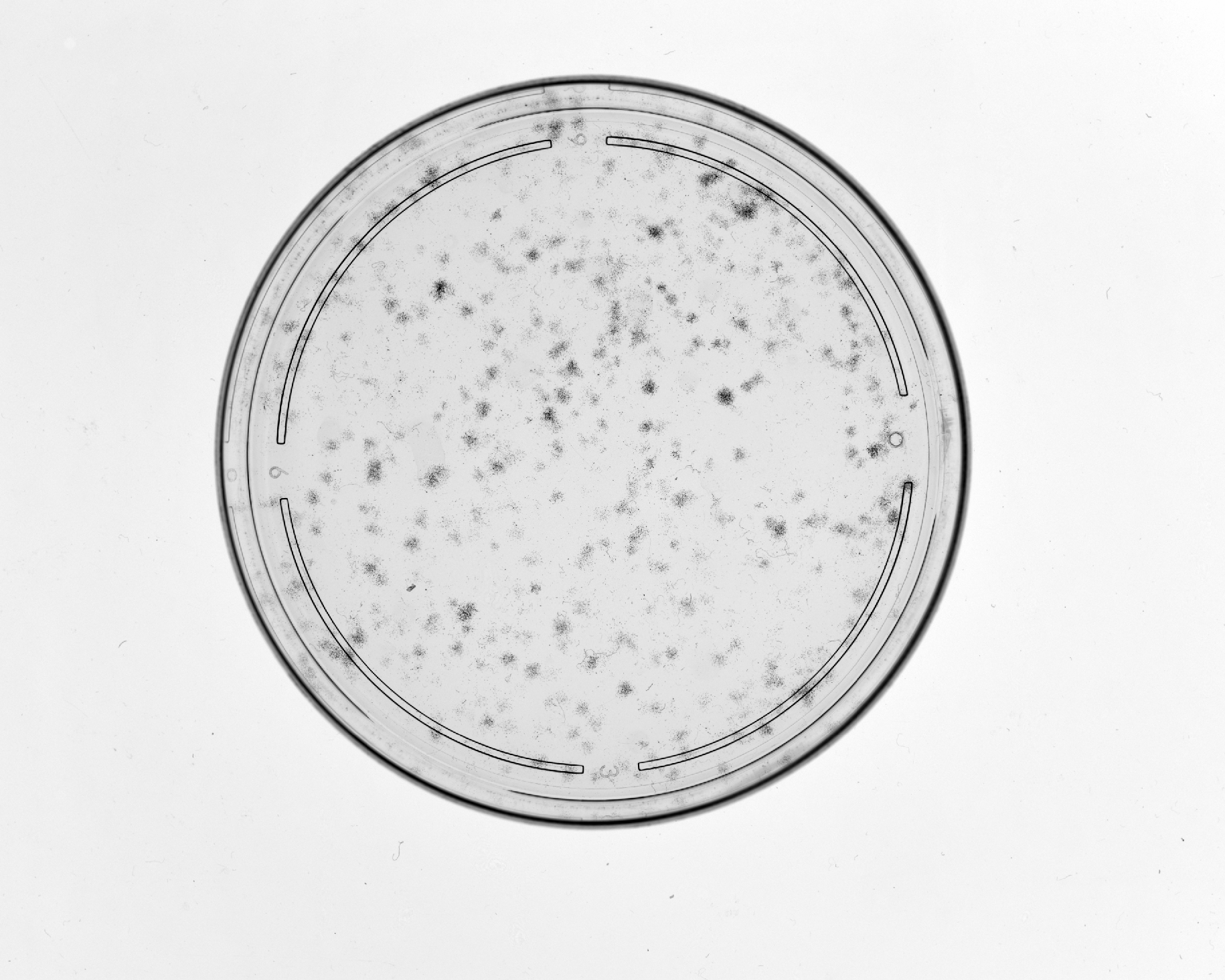

Supplement: Supplementary file 10 — Source Data for Figure 2 [file EMBR-24-e57234-s009.zip › Figure 2/2A/E564P clone 1 NT #1.tif]

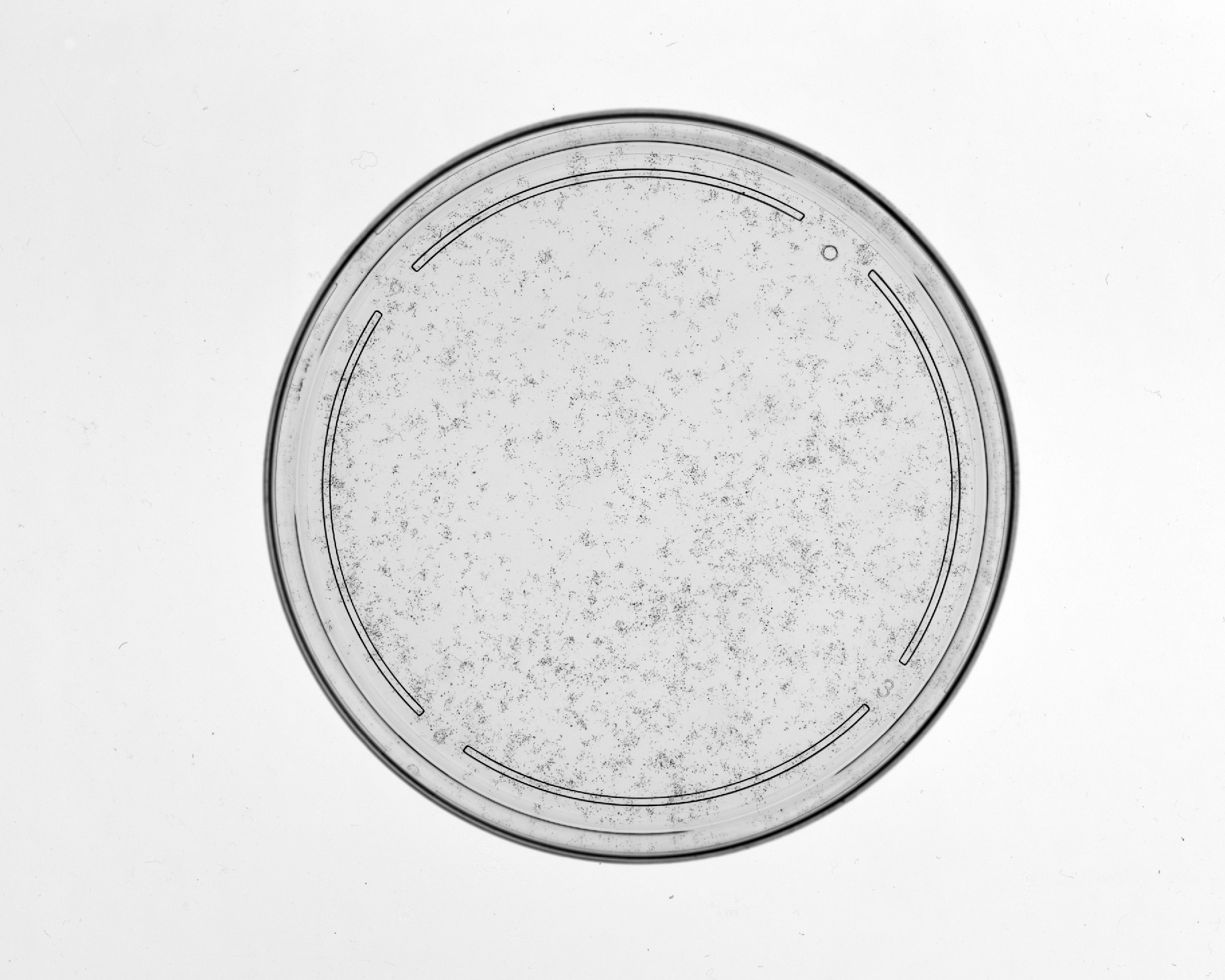

Supplement: Supplementary file 10 — Source Data for Figure 2 [file EMBR-24-e57234-s009.zip › Figure 2/2A/E564P clone 2 centrinone.tif]

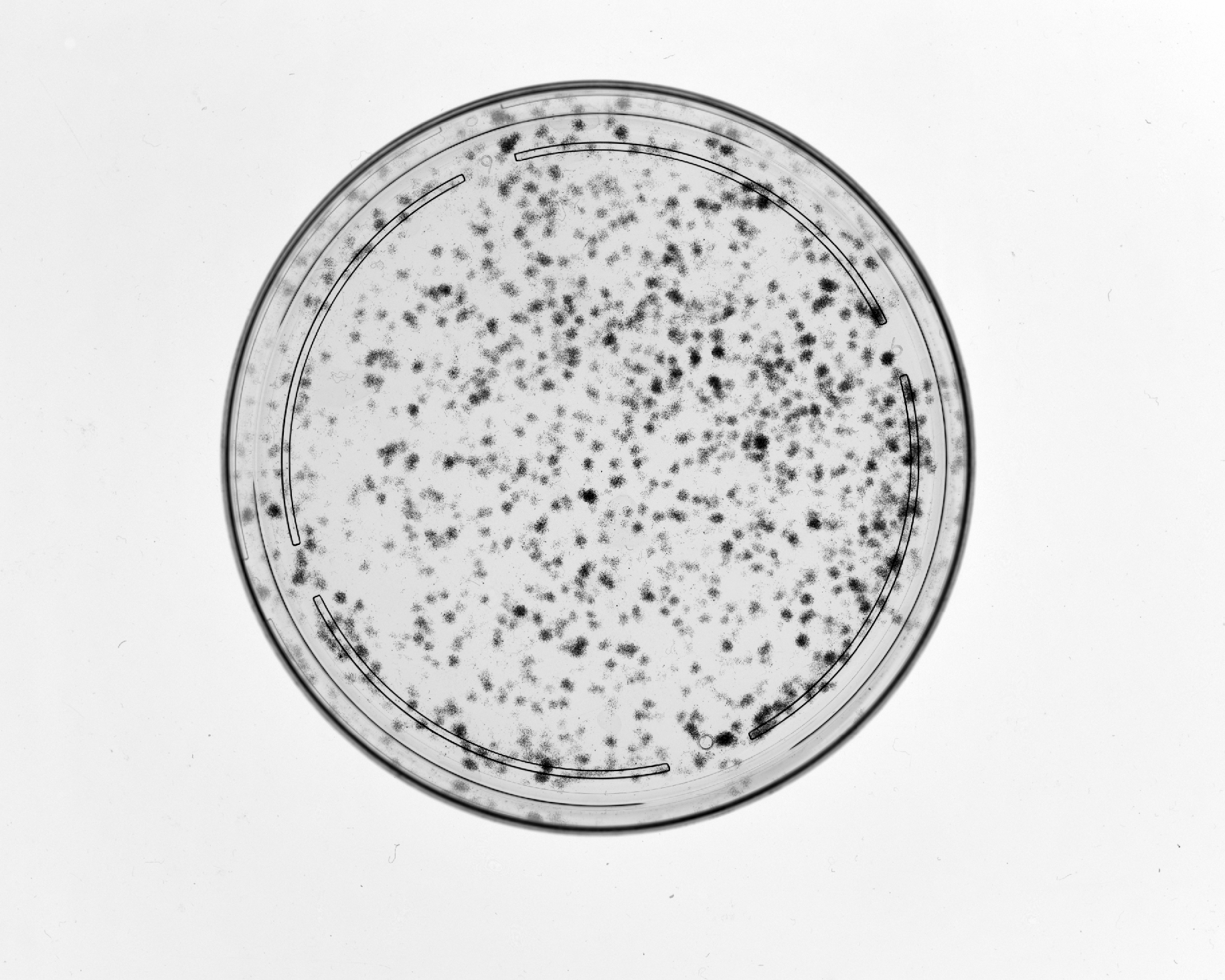

Supplement: Supplementary file 10 — Source Data for Figure 2 [file EMBR-24-e57234-s009.zip › Figure 2/2A/E564P clone 2 NT #1.tif]

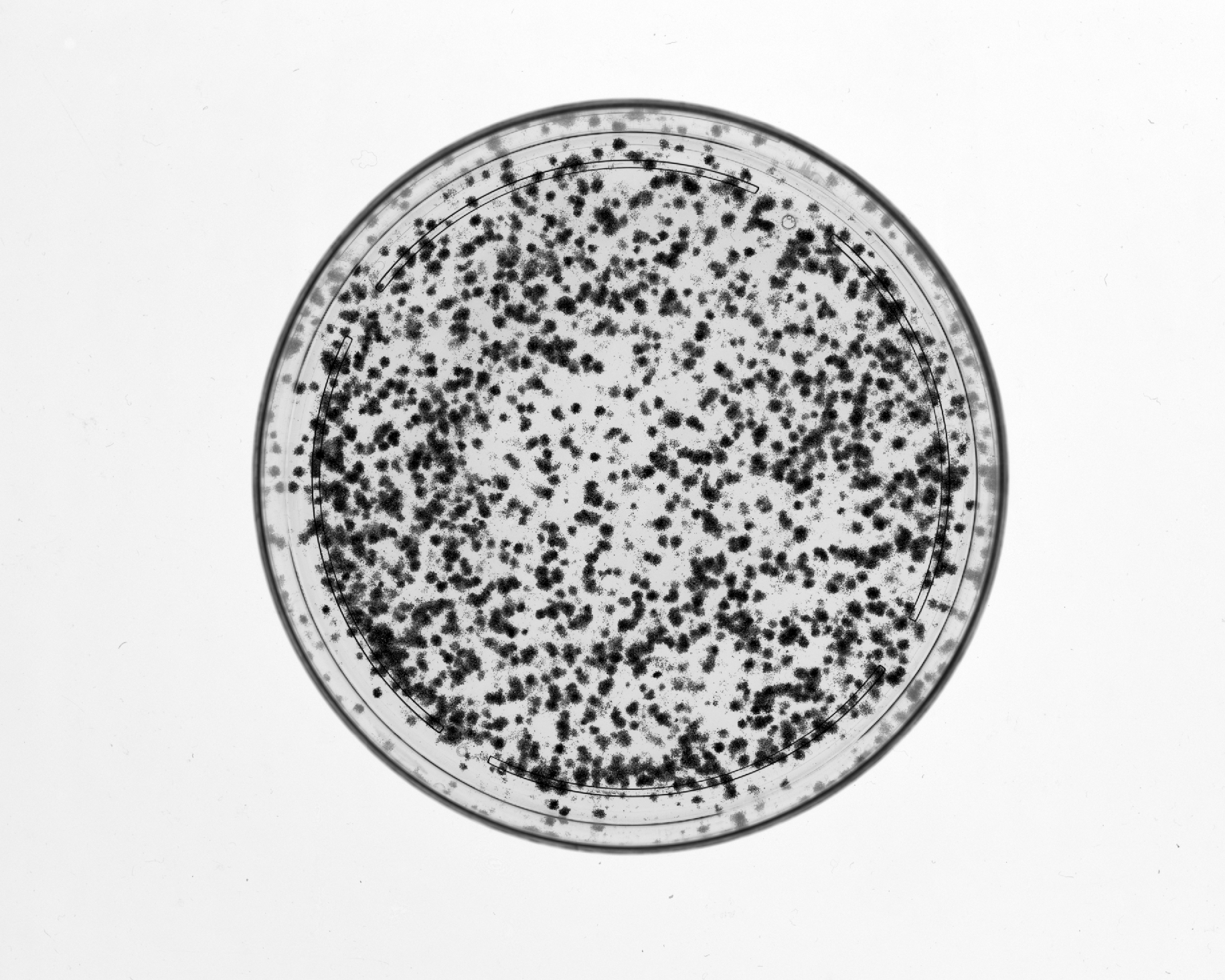

Supplement: Supplementary file 10 — Source Data for Figure 2 [file EMBR-24-e57234-s009.zip › Figure 2/2A/TP53BP1 KO centrinone.tif]

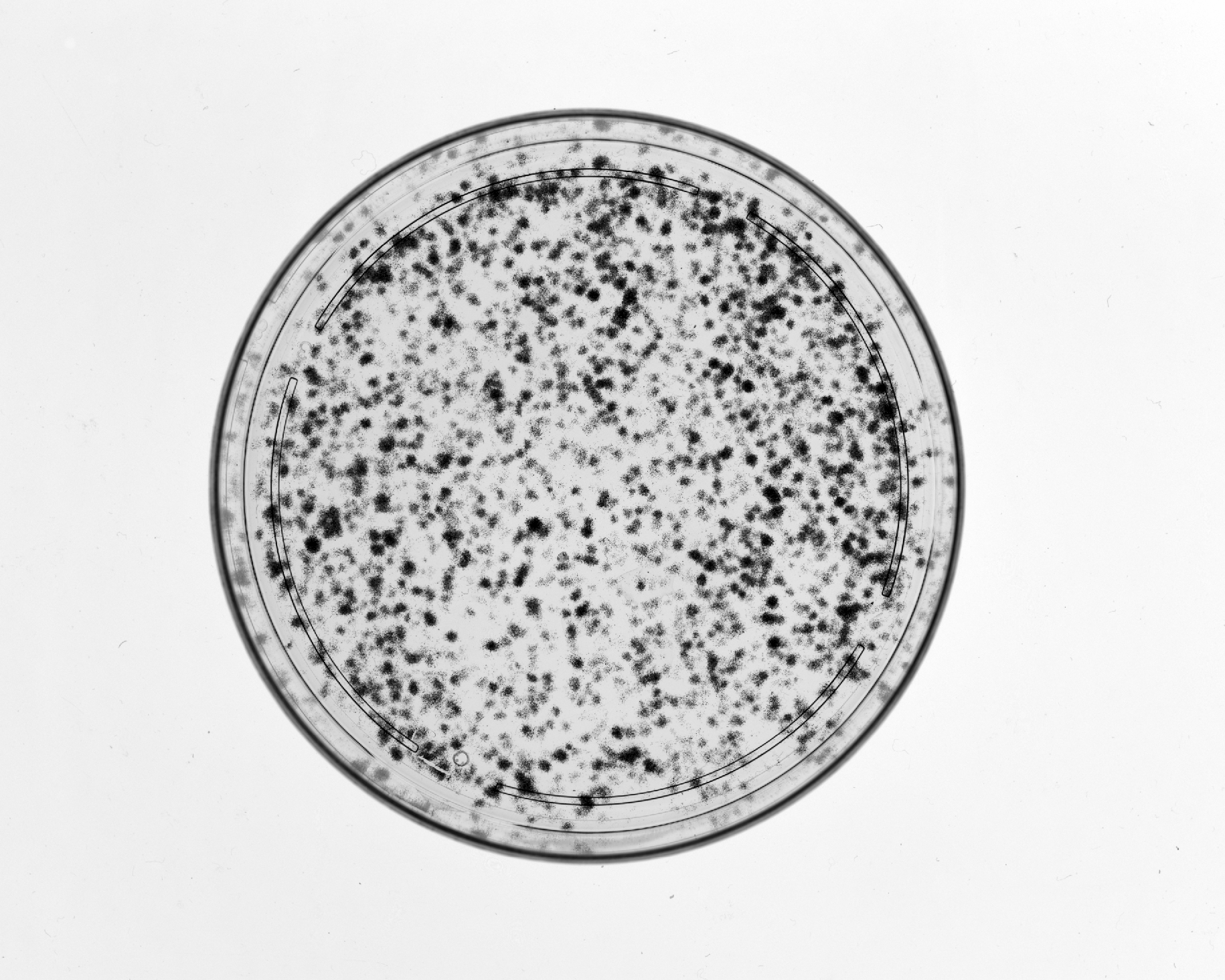

Supplement: Supplementary file 10 — Source Data for Figure 2 [file EMBR-24-e57234-s009.zip › Figure 2/2A/TP53BP1 KO NT #1.tif]

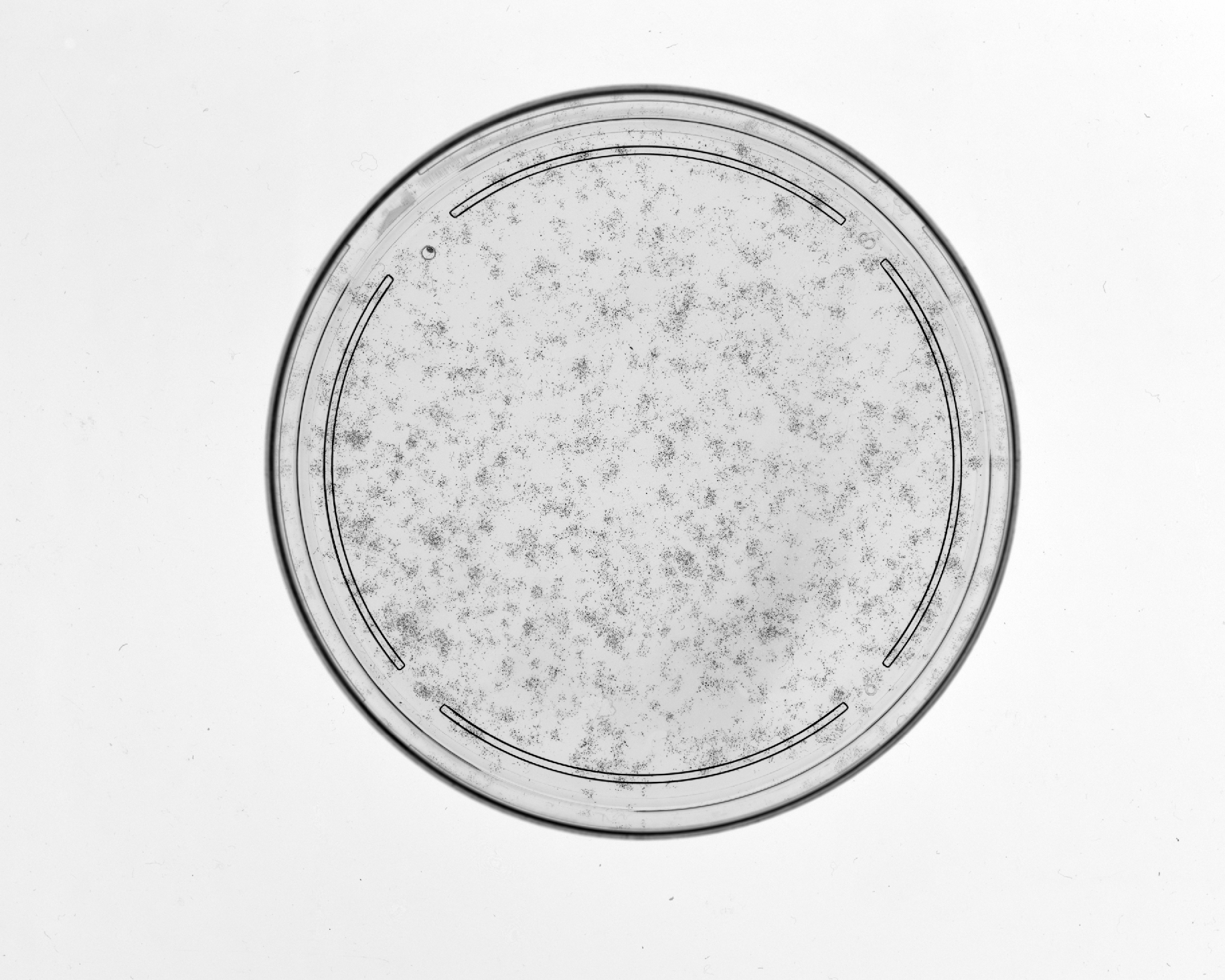

Supplement: Supplementary file 10 — Source Data for Figure 2 [file EMBR-24-e57234-s009.zip › Figure 2/2A/WT centrinone.tif]

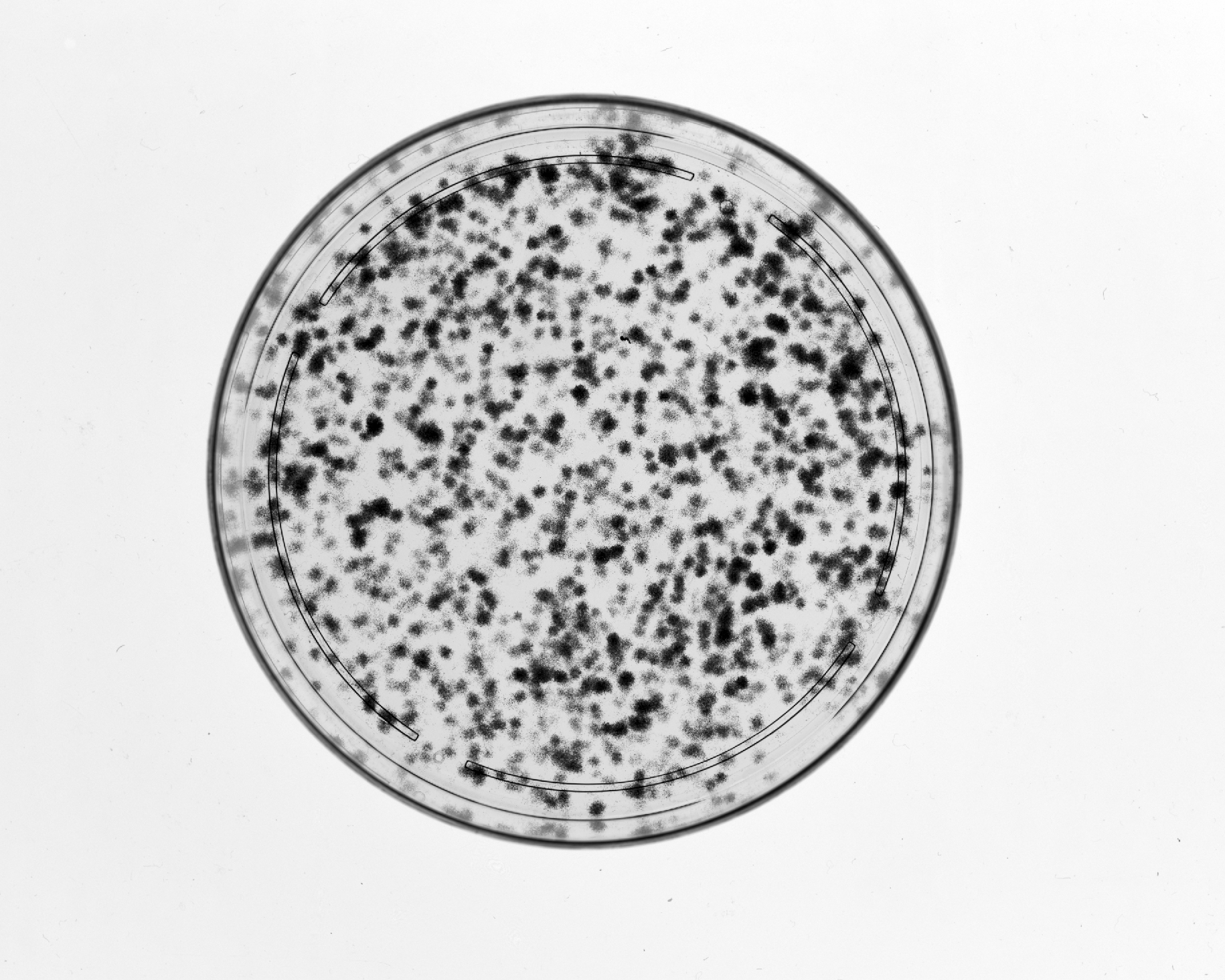

Supplement: Supplementary file 10 — Source Data for Figure 2 [file EMBR-24-e57234-s009.zip › Figure 2/2A/WT NT #1.tif]

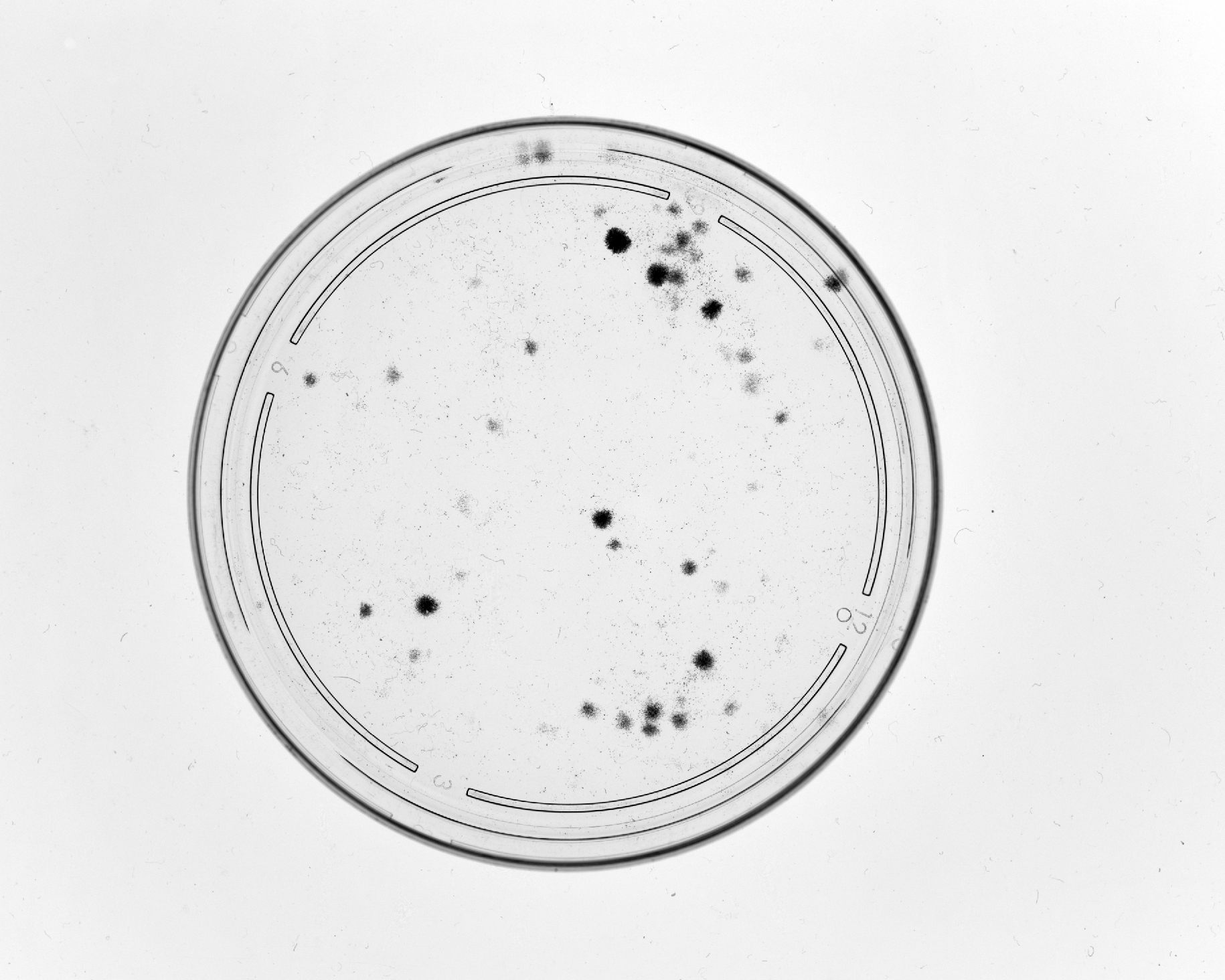

Supplement: Supplementary file 10 — Source Data for Figure 2 [file EMBR-24-e57234-s009.zip › Figure 2/2C/E564P BULK nocodazole.tif]

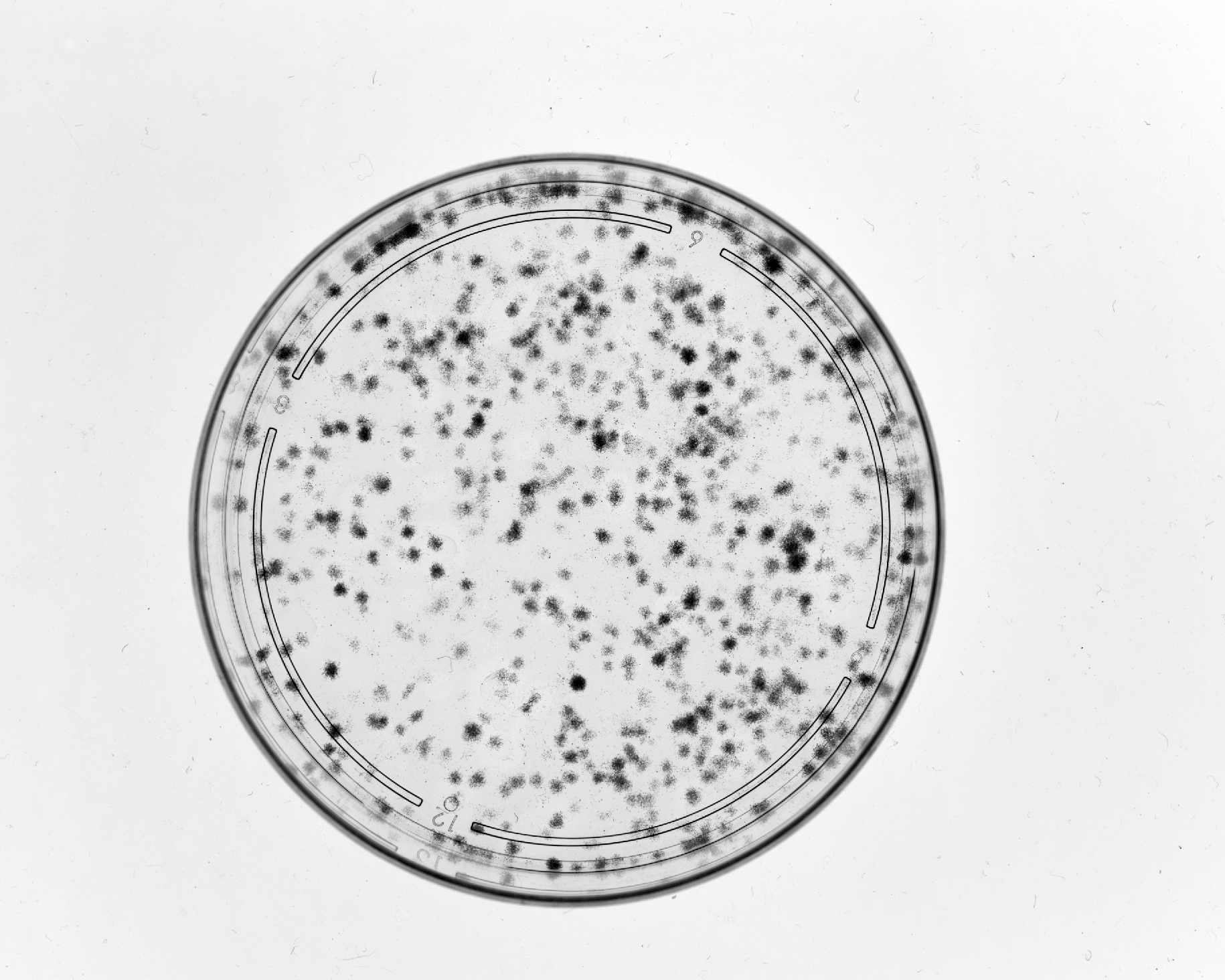

Supplement: Supplementary file 10 — Source Data for Figure 2 [file EMBR-24-e57234-s009.zip › Figure 2/2C/E564P BULK NT.tif]

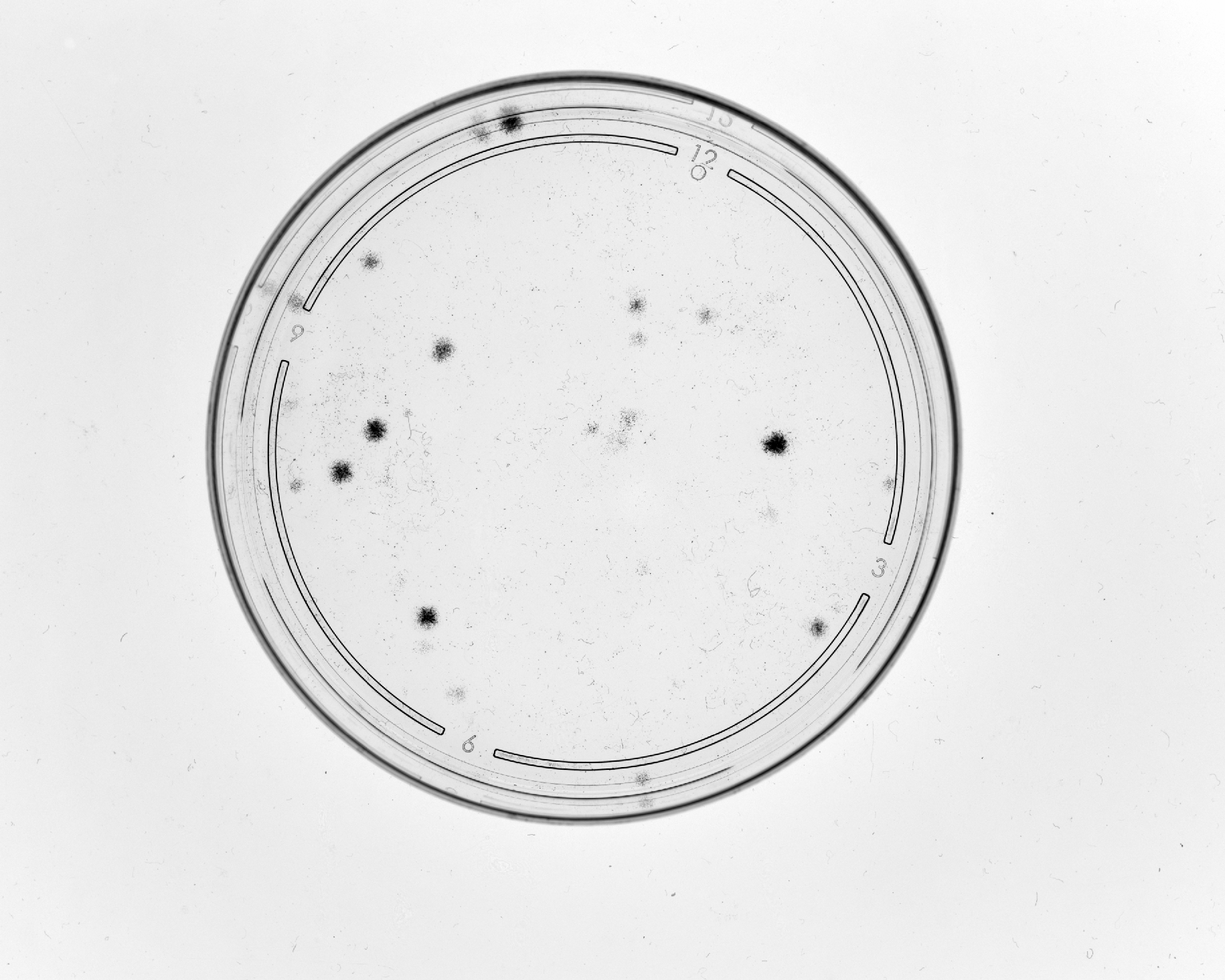

Supplement: Supplementary file 10 — Source Data for Figure 2 [file EMBR-24-e57234-s009.zip › Figure 2/2C/E564P clone 1 nocodazole.tif]

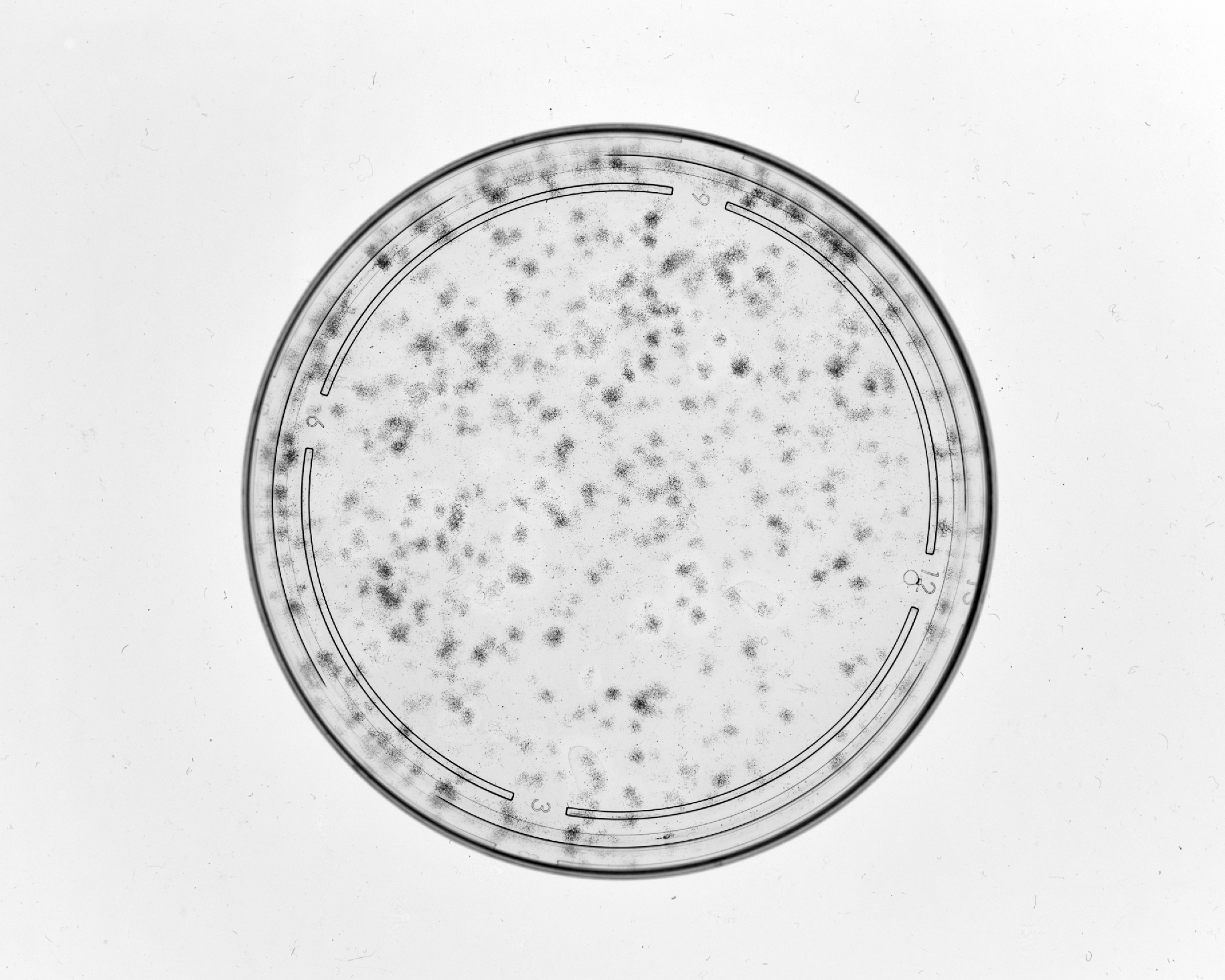

Supplement: Supplementary file 10 — Source Data for Figure 2 [file EMBR-24-e57234-s009.zip › Figure 2/2C/E564P clone 1 NT.tif]

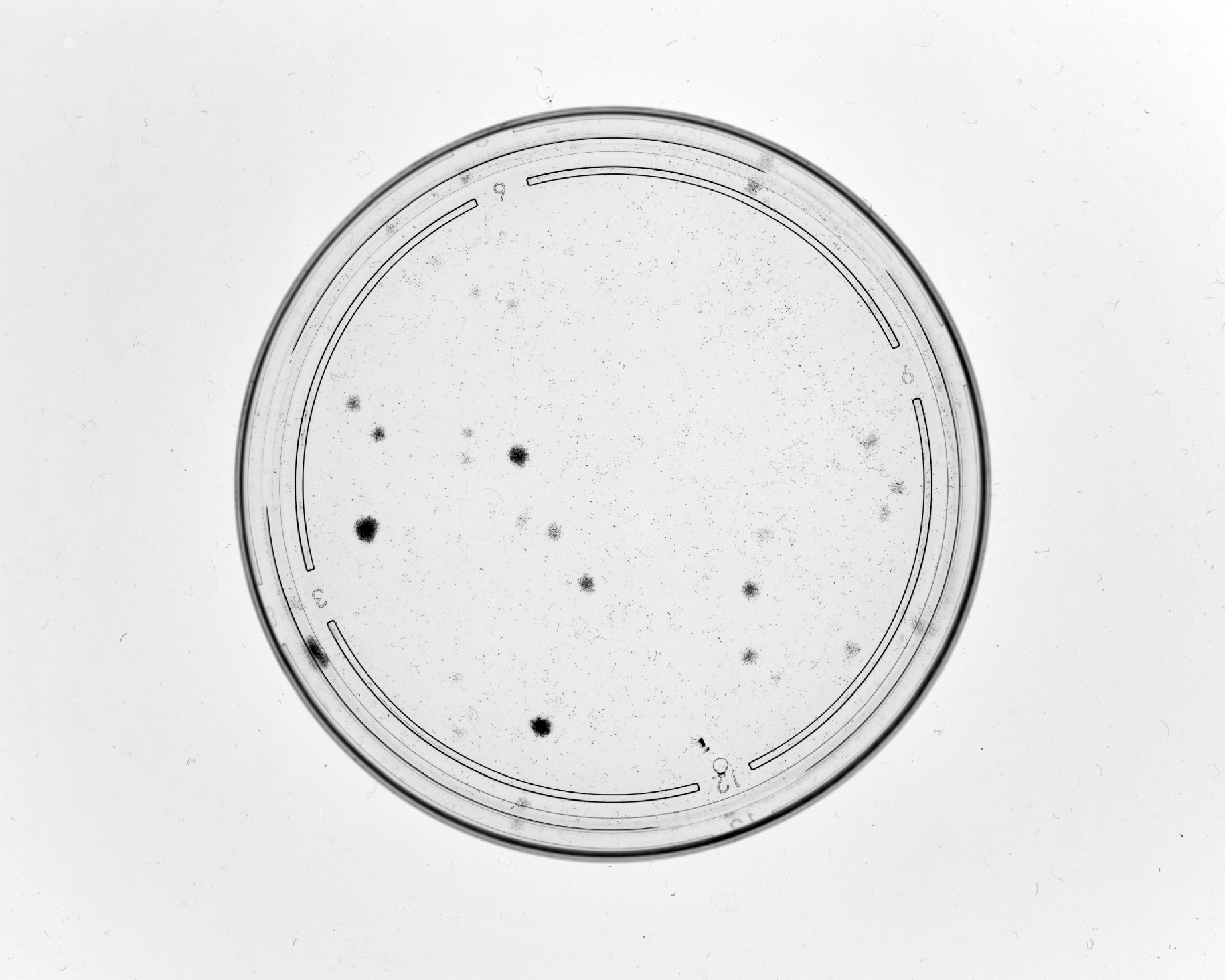

Supplement: Supplementary file 10 — Source Data for Figure 2 [file EMBR-24-e57234-s009.zip › Figure 2/2C/E564P clone 2 nocodazole.tif]

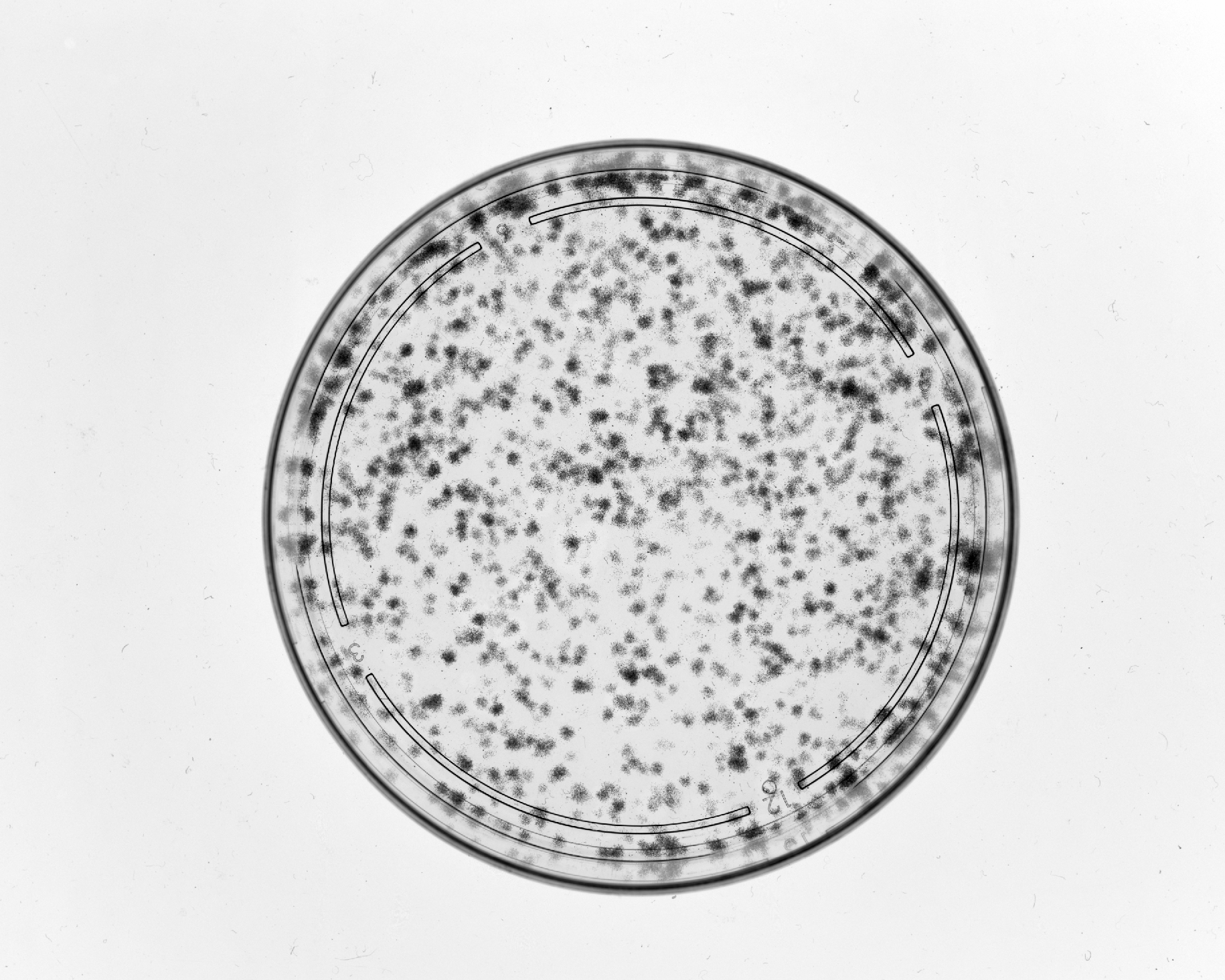

Supplement: Supplementary file 10 — Source Data for Figure 2 [file EMBR-24-e57234-s009.zip › Figure 2/2C/E564P clone 2 NT.tif]

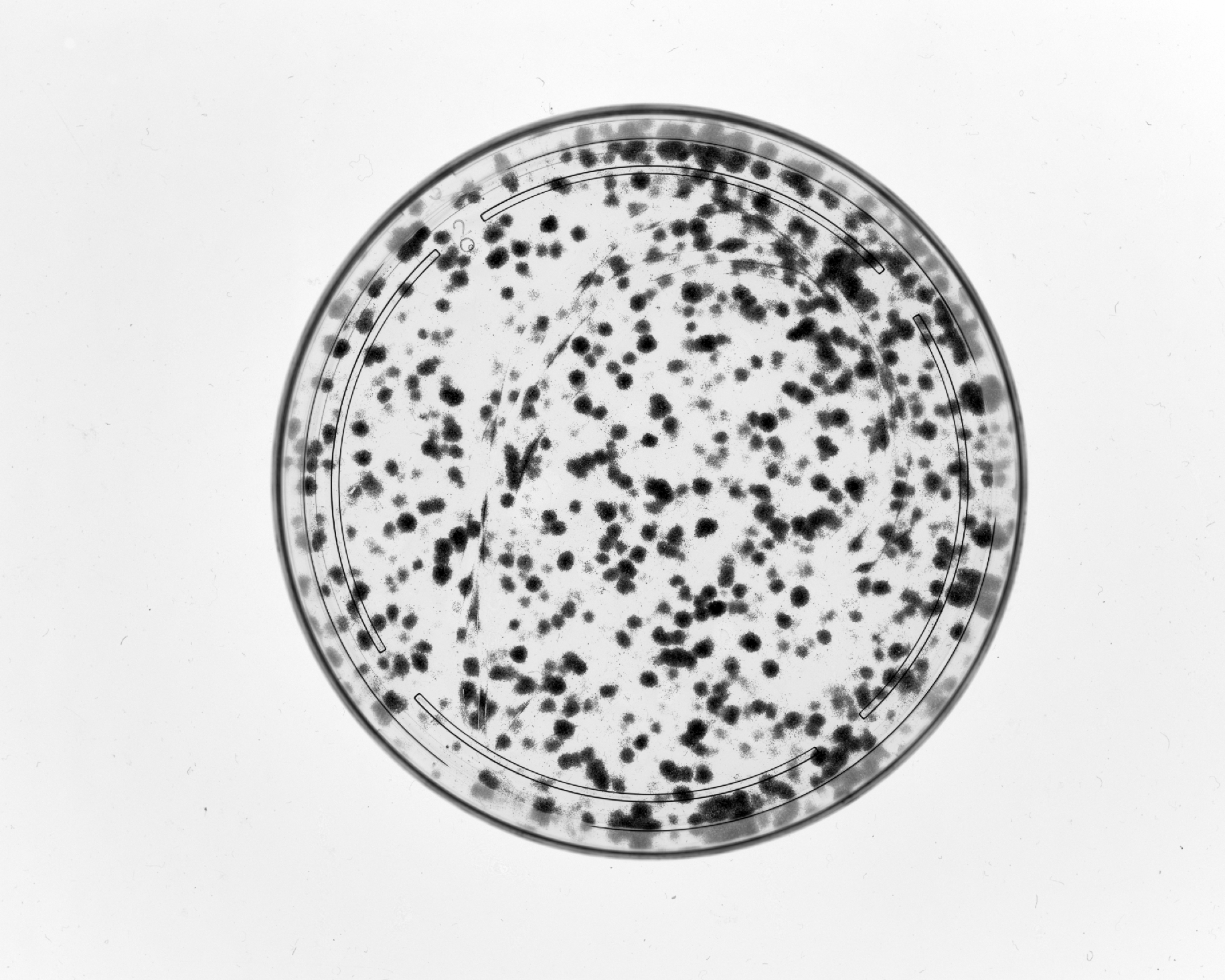

Supplement: Supplementary file 10 — Source Data for Figure 2 [file EMBR-24-e57234-s009.zip › Figure 2/2C/TP53BP1 KO nocodazole.tif]

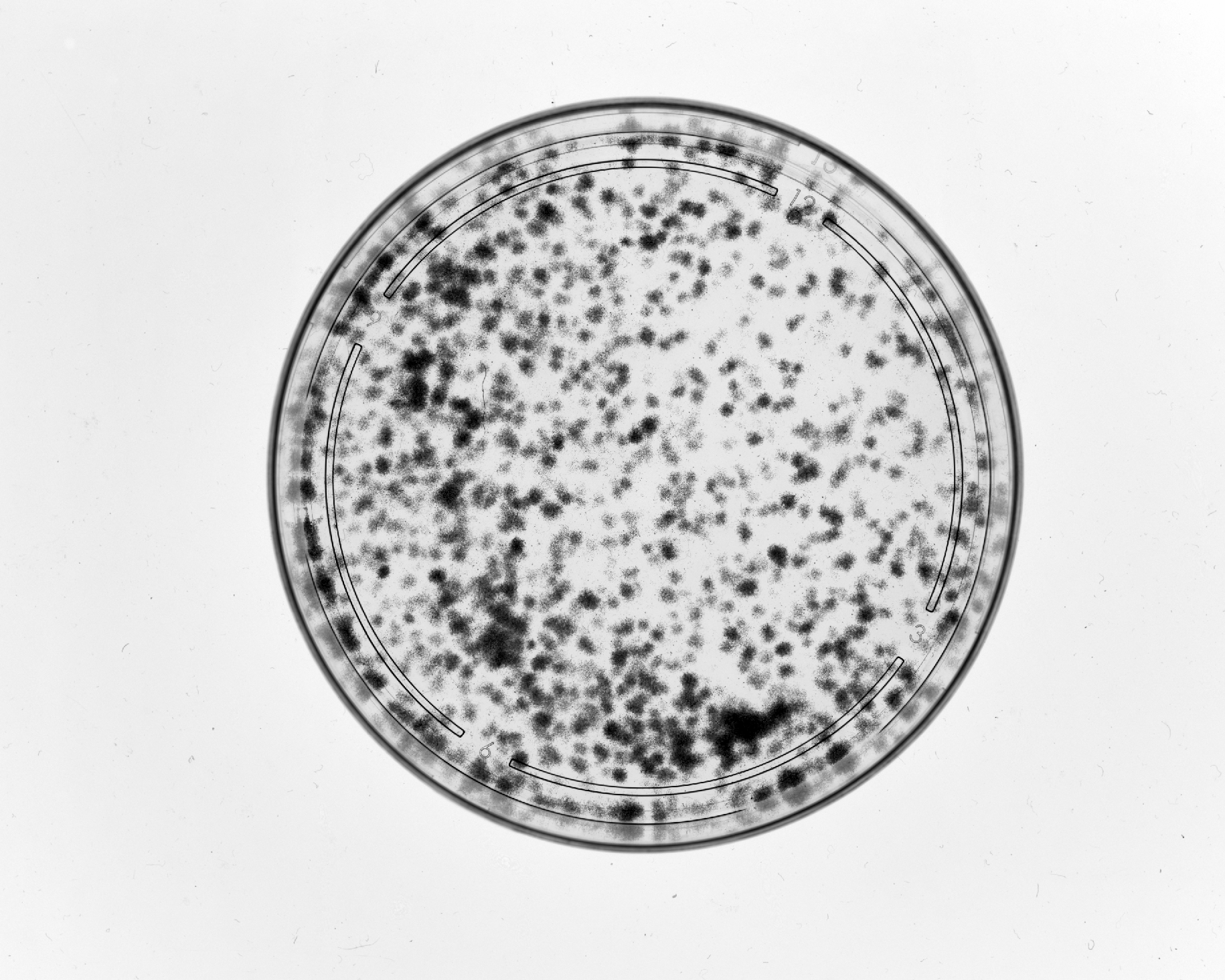

Supplement: Supplementary file 10 — Source Data for Figure 2 [file EMBR-24-e57234-s009.zip › Figure 2/2C/TP53BP1 KO NT.tif]

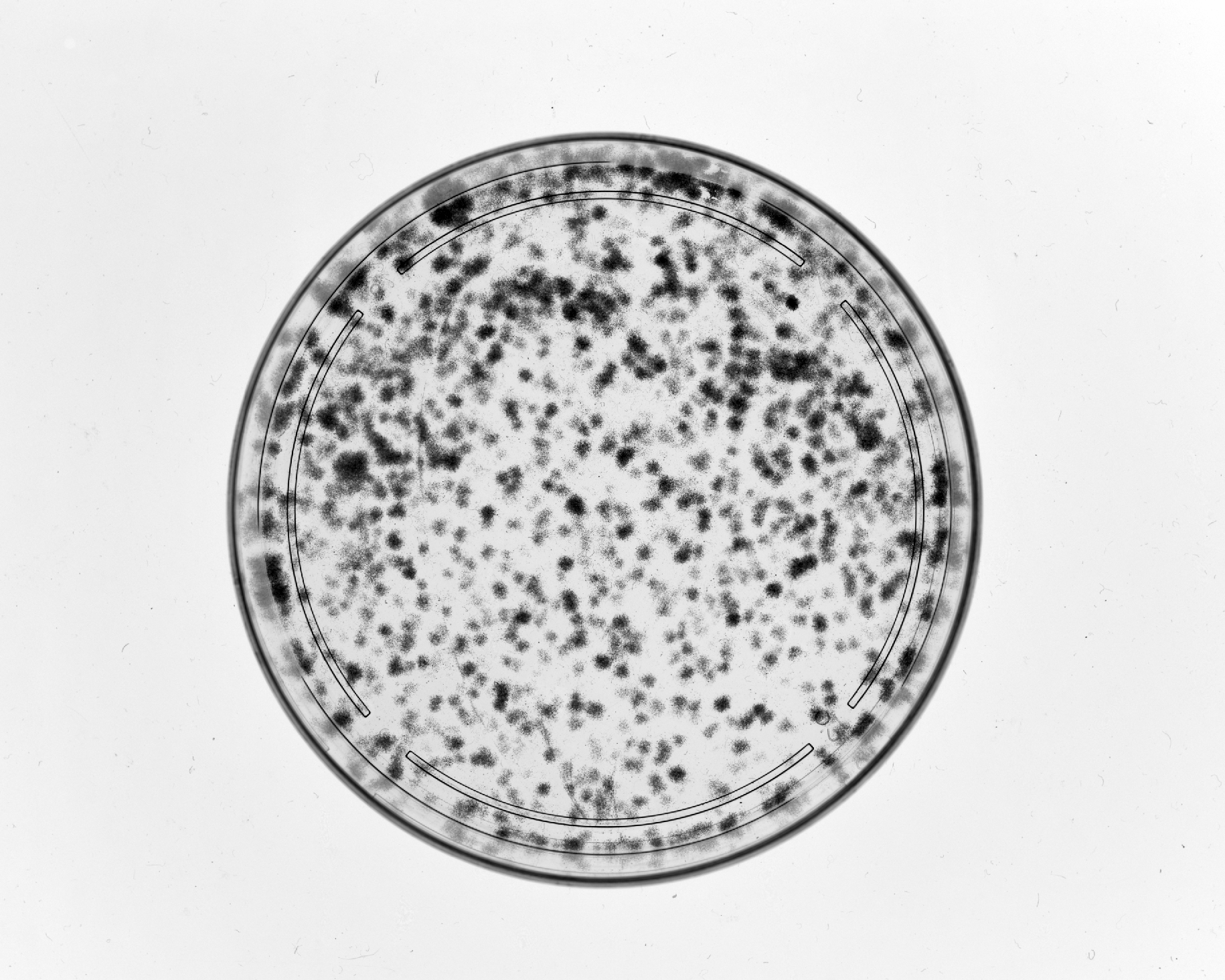

Supplement: Supplementary file 10 — Source Data for Figure 2 [file EMBR-24-e57234-s009.zip › Figure 2/2C/WT nocodazole.tif]

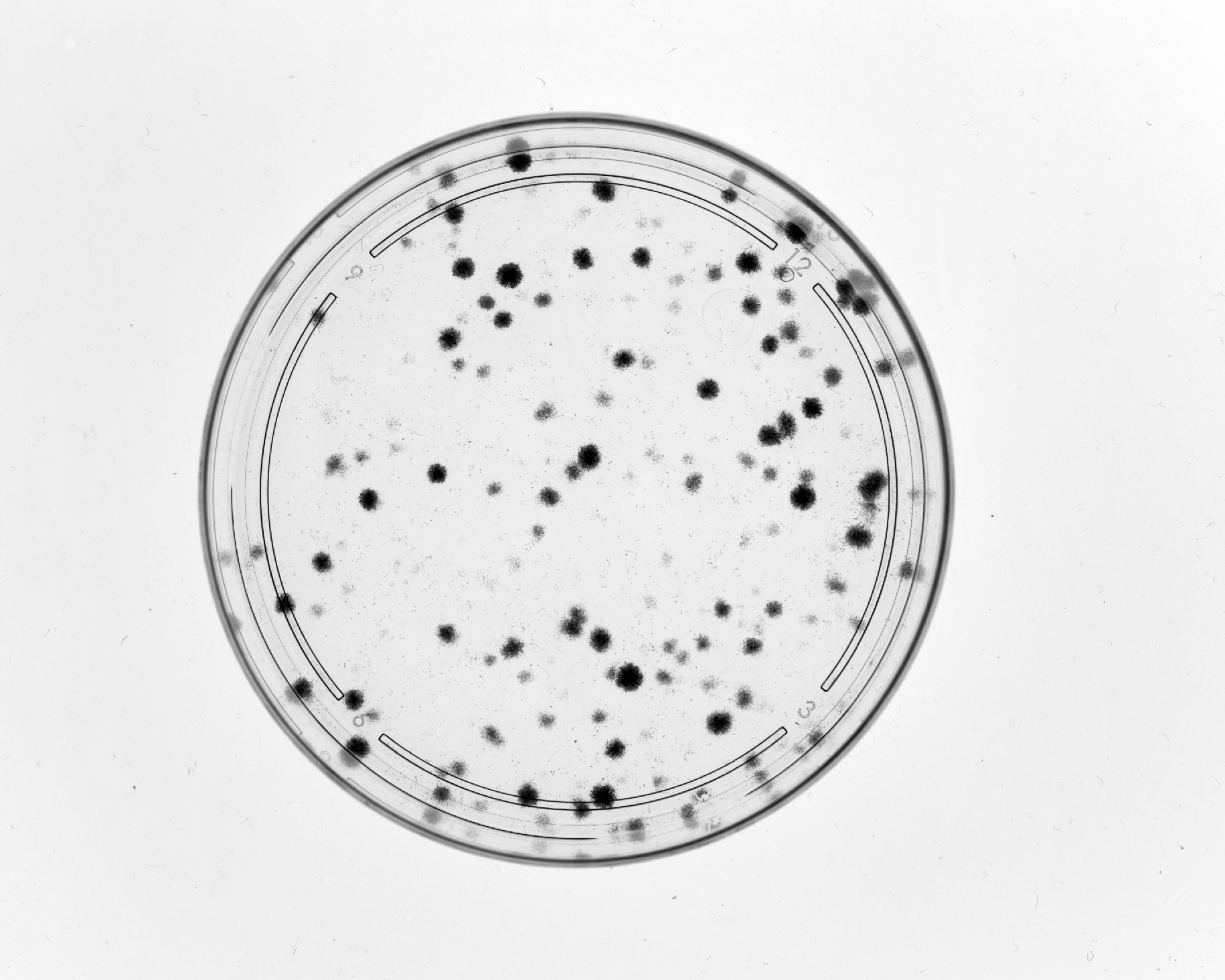

Supplement: Supplementary file 10 — Source Data for Figure 2 [file EMBR-24-e57234-s009.zip › Figure 2/2C/WT NT.tif]

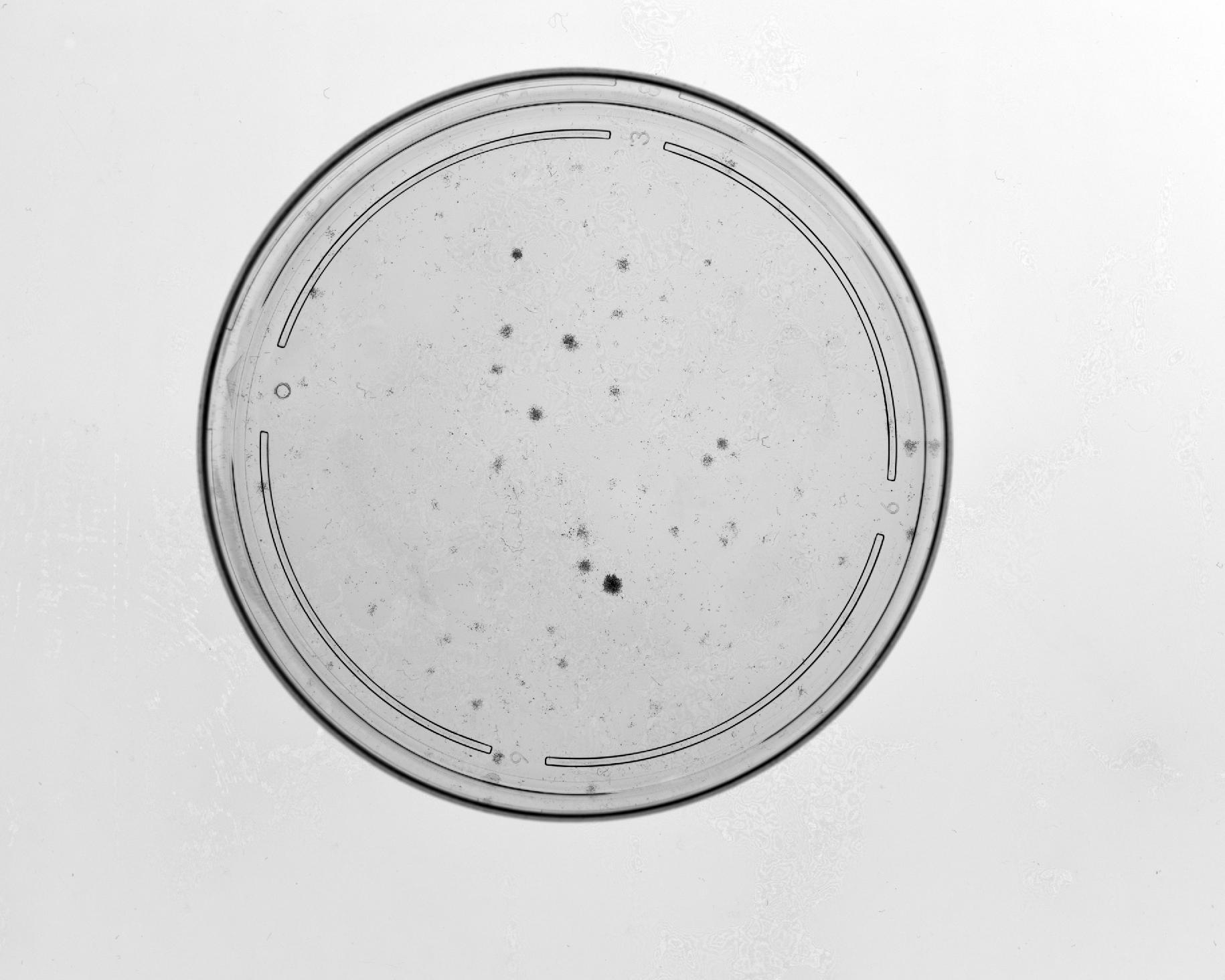

Supplement: Supplementary file 12 — Source Data for Figure 4 [file EMBR-24-e57234-s012.zip › Figure 4/4B/E564P M+6h (3MBPP1).tif]

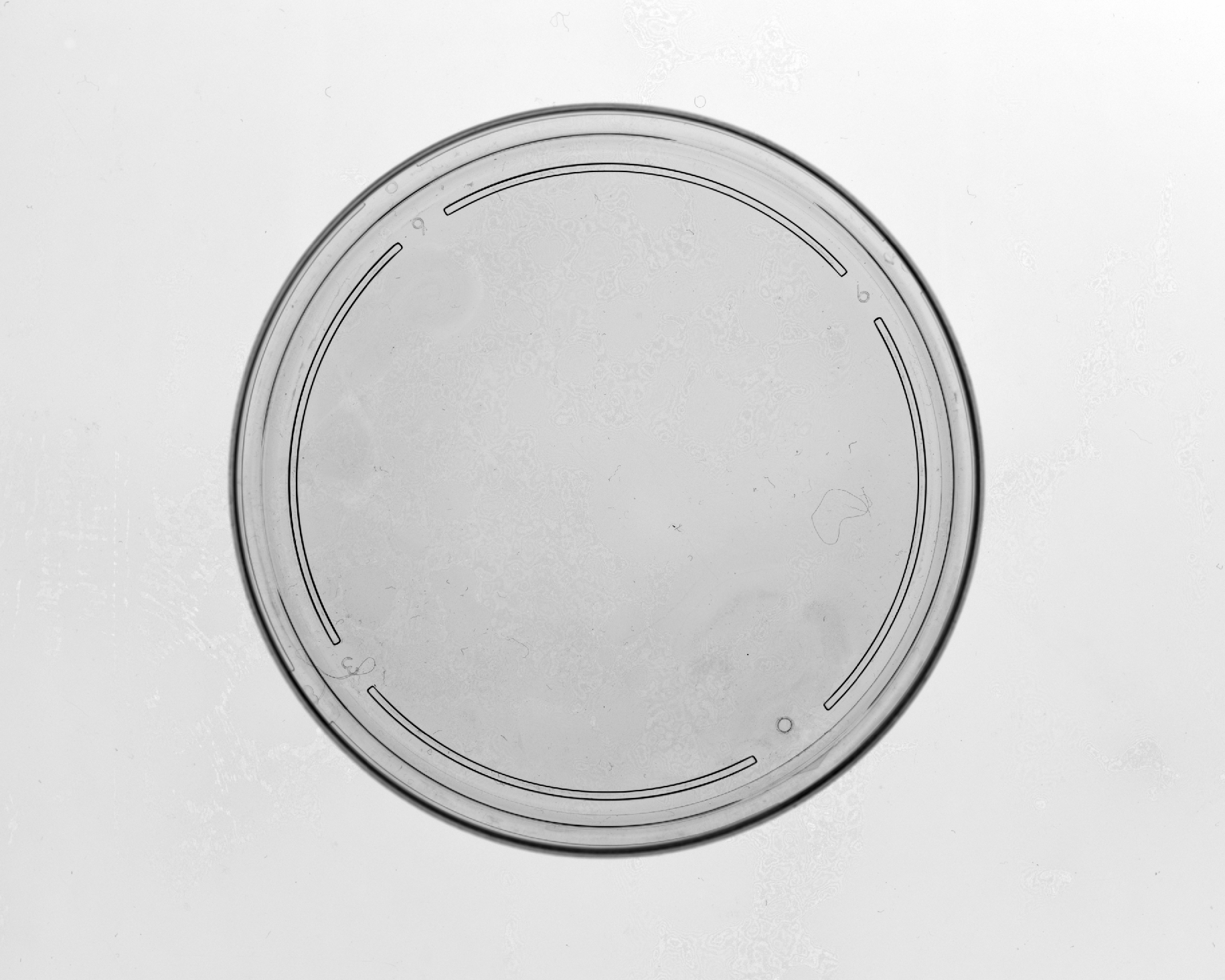

Supplement: Supplementary file 12 — Source Data for Figure 4 [file EMBR-24-e57234-s012.zip › Figure 4/4B/E564P M+6h.tif]

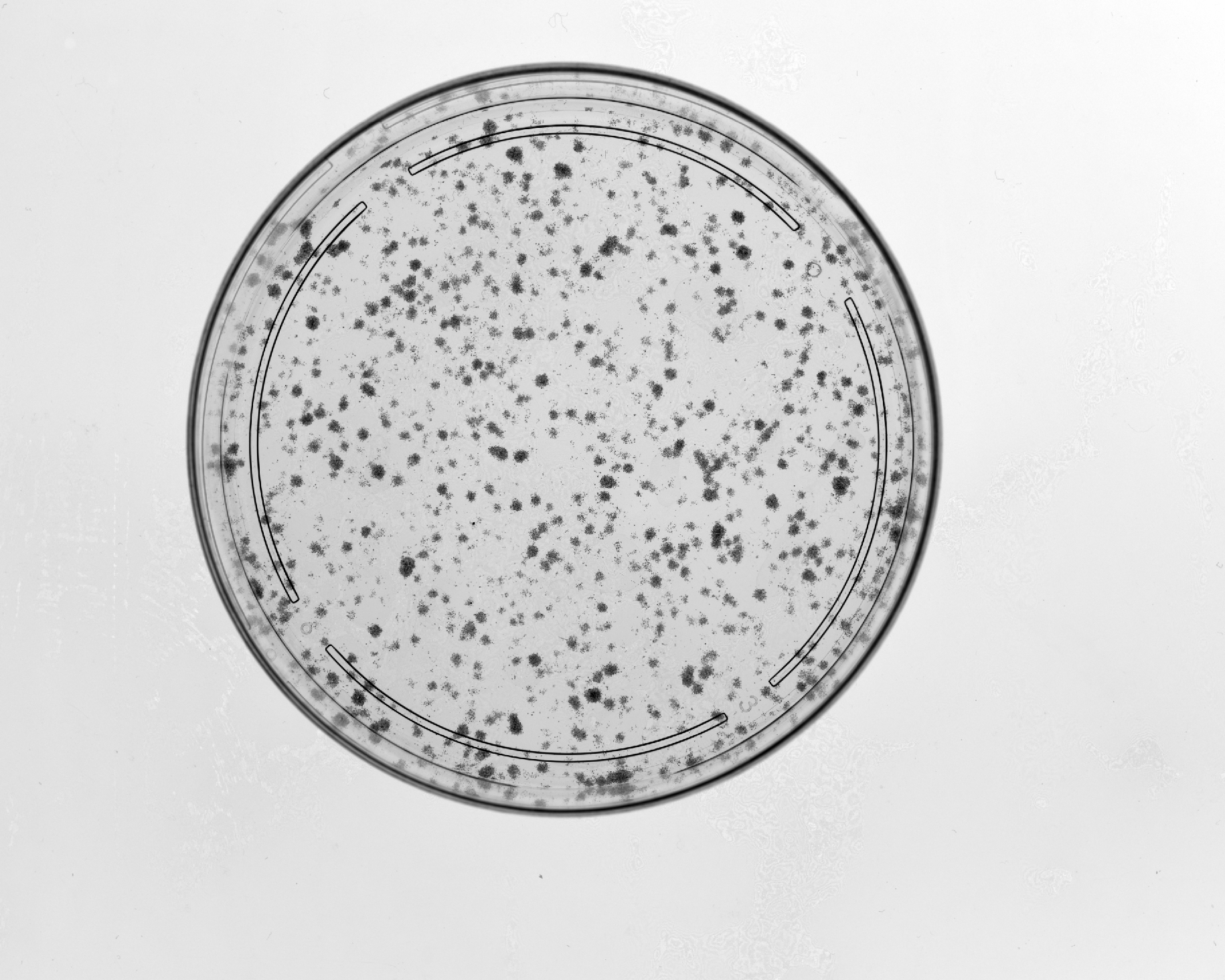

Supplement: Supplementary file 12 — Source Data for Figure 4 [file EMBR-24-e57234-s012.zip › Figure 4/4B/TP53BP1 KO M+6h (3MBPP1).tif]

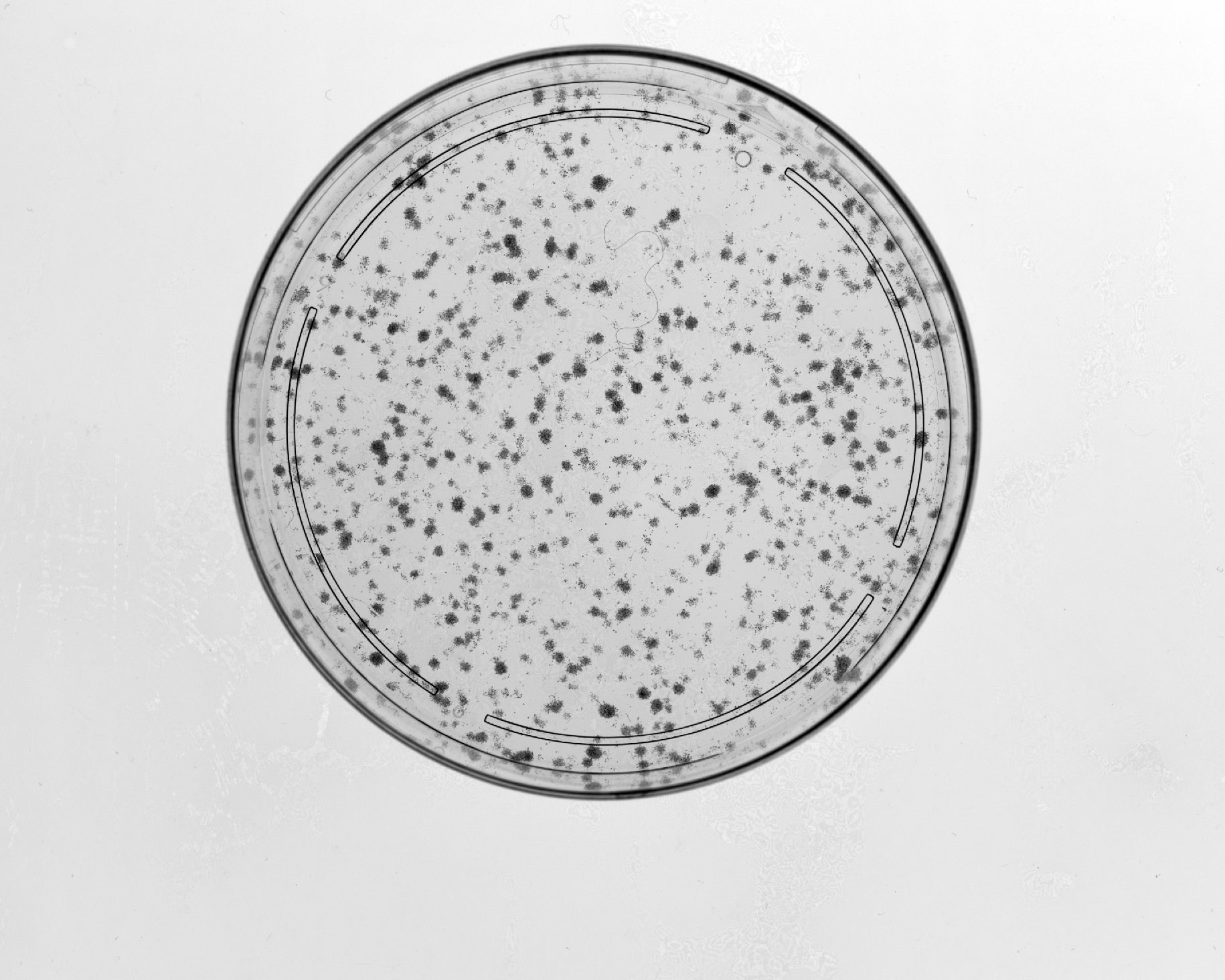

Supplement: Supplementary file 12 — Source Data for Figure 4 [file EMBR-24-e57234-s012.zip › Figure 4/4B/TP53BP1 KO M+6h.tif]

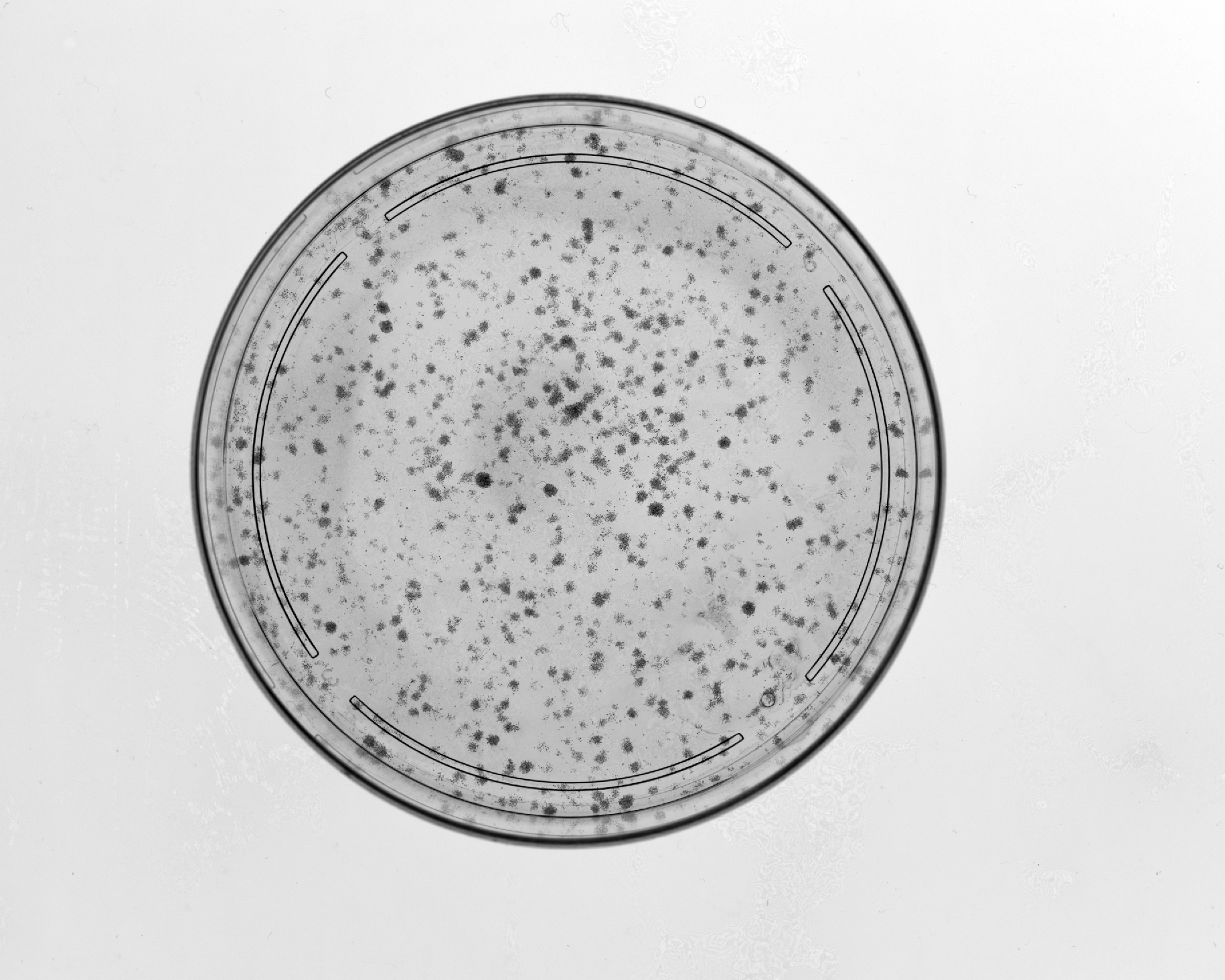

Supplement: Supplementary file 12 — Source Data for Figure 4 [file EMBR-24-e57234-s012.zip › Figure 4/4B/WT M+6h (3MBPP1).tif]

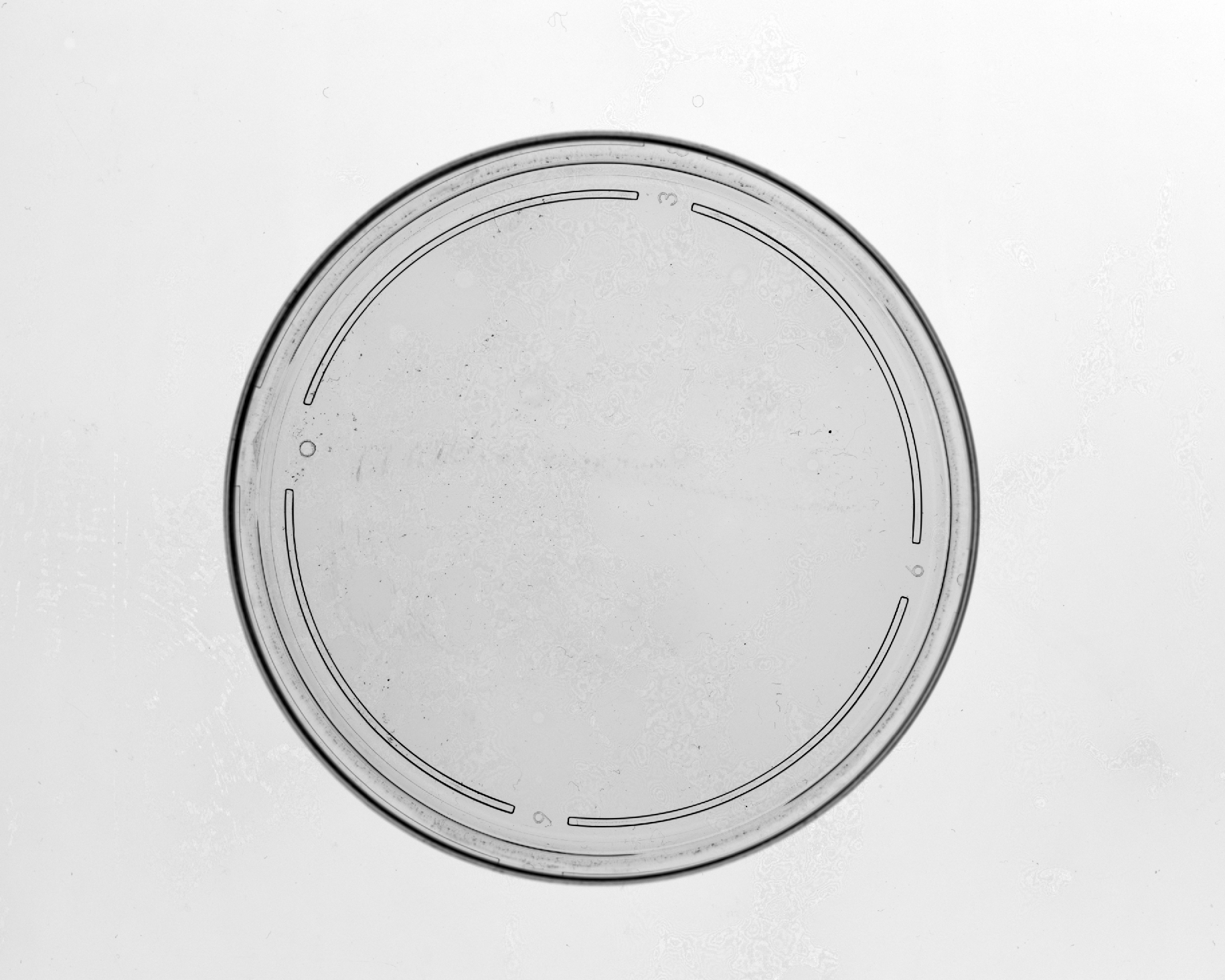

Supplement: Supplementary file 12 — Source Data for Figure 4 [file EMBR-24-e57234-s012.zip › Figure 4/4B/WT M+6h.tif]
