# Supplementary material for: PLK1 promotes the mitotic surveillance pathway by controlling cytosolic 53BP1 availability
Source: EMBO Rep. 2023 Oct 27;24(12):e57234. doi: 10.15252/embr.202357234 (PMC10702821; doi:10.15252/embr.202357234)
Supplement: Supplementary file 13 [file EMBR-24-e57234-s002.pdf]

EMBO reports

PLK1 promotes the mitotic surveillance pathway by controlling cytosolic 53BP1 availability

Matteo Burigotto, Vincenza Vigorito, Colin Gliech, Alessia Mattivi, Sabrina Ghetti, Alessandra Bisio, Graziano Lolli, Andrew Holland, and Luca Fava
DOI: 10.15252/embr.202357234

Corresponding author(s): Luca Fava (luca.fava@unitn.it) , Matteo Burigotto (matteo.burigotto@kcl.ac.uk)

Review Timeline:

Submission Date:	24th Mar 23
Editorial Decision:	11th May 23
Revision Received:	19th Aug 23
Editorial Decision:	19th Sep 23
Revision Received:	4th Oct 23
Accepted:	9th Oct 23

Editor: Ioannis Papaioannou / Achim Breiling

Transaction Report:

(Note: With the exception of the correction of typographical or spelling errors that could be a source of ambiguity, letters and reports are not edited. Depending on transfer agreements, referee reports obtained elsewhere may or may not be included in this compilation. Referee reports are anonymous unless the Referee chooses to sign their reports.)

Dear Luca,

Thank you for submitting your research manuscript for consideration by EMBO reports. It has now been seen by four experts in the field, and we have received the full set of their reports that I have already shared with you (they are included again below). The referees acknowledge that the topic is significant and the findings potentially interesting, but they also identify a number of limitations and raise several concerns.

In light of the referees' reports, I have asked you to provide us with a point-by-point response outlining whether you are able and willing to address the comments and concerns of the referees. I would like to thank you for your detailed feedback and your revision plan, as well as our subsequent discussion, which were very helpful in reaching a fair and balanced decision on your manuscript.

Given the constructive comments of the reviewers, we would like to invite you to revise your manuscript along the lines of your revision plan, with the understanding that the referee suggestions must be taken on board and all of their concerns fully addressed in a complete point-by-point response. Acceptance of the manuscript will depend on a positive outcome of a second round of review. If you have any questions or comments, we can also discuss the revisions in a video chat, if you like.

We realize that it is difficult to revise to a specific deadline. In the interest of protecting the conceptual advance provided by the work, we usually recommend a revision within 3 months (August 10th). Please discuss with me the revision progress ahead of this time if you require more time to complete the revisions.

Please note that you can format your manuscript either as a Report or as an Article. For Reports, the manuscript should not exceed 27,000 characters (including spaces but excluding Materials & Methods and References) and 5 main plus 5 Expanded View figures. The Results and Discussion sections must be combined, which will help to shorten the manuscript text by eliminating some redundancy that is inevitable when discussing the same experiments twice. For an Article there are no length limitations, but it should have more than 5 main figures and the Results and Discussion sections must be separate. In both cases, the entire Materials and Methods must be included in the main manuscript file.

*****
IMPORTANT NOTE:
We perform an initial quality control of all revised manuscripts before re-review. Your manuscript will FAIL this control and the handling will be DELAYED if the following APPLIES:

1) If a data availability section providing access to data deposited in public databases is missing (please see below for more information).

2) If your manuscript contains statistics and error bars based on n=2. Please use scatter plots in these cases. No statistics should be calculated if n=2.

When submitting your revised manuscript, please carefully review the instructions that follow below. Failure to include requested items will delay the evaluation of your revision.
*****

When submitting your revised manuscript, we will require:

1) A .docx formatted version of the manuscript text (including legends for main figures, EV figures and tables). Please make sure that the changes are highlighted to be clearly visible.

2) Individual production quality figure files as .eps, .tif, .jpg (one file per figure).
Please download our Figure Preparation Guidelines (figure preparation pdf) from our Author Guidelines pages https://www.embopress.org/page/journal/14693178/authorguide for more info on how to prepare your figures.

3) A .docx formatted letter INCLUDING the reviewers' reports and your detailed point-by-point responses to their comments. As part of the EMBO Press transparent editorial process, the point-by-point response is part of the Review Process File (RPF), which will be published alongside your paper unless you opt out of this (please see below for further information).

4) A complete author checklist, which you can download from our author guidelines (). Please insert information in the checklist that is also reflected in the manuscript. The completed author checklist will also be part of the RPF (please see below for more information).

5) Please note that all corresponding authors are required to supply an ORCID ID for their name upon submission of a revised manuscript (). Please find instructions on how to link your ORCID ID to your account in our manuscript tracking system in our Author guidelines
()

6) We replaced Supplementary Information with Expanded View (EV) Figures and Tables that are collapsible/expandable online. A maximum of 5 EV Figures can be typeset. EV Figures should be cited as 'Figure EV1, Figure EV2" etc... in the text and their respective legends should be included in the main text after the legends of regular figures.

- For the figures that you do NOT wish to display as Expanded View figures, they should be bundled together with their legends in a single PDF file called *Appendix*, which should start with a short Table of Content. Appendix figures should be referred to in the main text as: "Appendix Figure S1, Appendix Figure S2" etc. See detailed instructions regarding expanded view here:

- Additional Tables/Datasets should be labeled and referred to as Table EV1, Dataset EV1, etc. Legends have to be provided in a separate tab in case of .xls files. Alternatively, the legend can be supplied as a separate text file (README) and zipped together with the Table/Dataset file.

7) Before submitting your revision, primary datasets (and computer code, where appropriate) produced in this study need to be deposited in appropriate public databases (see <
https://www.embopress.org/page/journal/14693178/authorguide#dataavailability>).
Specifically, we would kindly ask you to provide public access to the following datasets/data:
- Mass spectrometry data.

Please remember to provide a reviewer password if the datasets are not yet public.

The accession numbers and database should be listed in a formal "Data availability " section (placed after Materials & Methods) that follows the model below (see also < https://www.embopress.org/page/journal/14693178/authorguide#dataavailability>):

Data availability

The datasets (and computer code) produced in this study are available in the following databases:

- RNA-seq data: Gene Expression Omnibus GSE46843 (https://www.ncbi.nlm.nih.gov/geo/query/acc.cgi?acc=GSE46843)
- [data type]: [name of the resource] [accession number/identifier/doi] ([URL or identifiers.org/DATABASE:ACCESSION])

*** Note: all links should resolve to a page where the data can be accessed. ***

*** Note: the Data Availability Section is restricted to new primary data that are part of this study. ***

8) We request authors to consider both actual and perceived competing interests. Please review the new policy () and update your competing interests statement if necessary. Please name this section 'Disclosure and competing interests statement' and place it after the Acknowledgements section.

9) Figure legends and data quantification:
The following points must be specified in each figure legend:

- the name of the statistical test used to generate error bars and P values,
- the number (n) of independent experiments (please specify technical or biological replicates) underlying each data point,
- the nature of the bars and error bars (s.d., s.e.m.)
- If the data are obtained from n {less than or equal to} 2, use scatter plots showing the individual data points.

Discussion of statistical methodology can be reported in the Materials and Methods section, but figure legends should contain a basic description of n, P and the test applied.

See also the guidelines for figure legend preparation:
https://www.embopress.org/page/journal/14693178/authorguide#figureformat

- Please also include scale bars in all microscopy images.

10) We now request publication of original source data with the aim of making primary data more accessible and transparent to the reader. Our source data coordinator will contact you to discuss which figure panels we would need source data for and will also provide you with helpful tips on how to upload and organize the files.

11) Our journal encourages inclusion of *data citations in the reference list* to directly cite datasets that were re-used and obtained from public databases. Data citations in the article text are distinct from normal bibliographical citations and should directly link to the database records from which the data can be accessed. In the main text, data citations are formatted as follows: "Data ref: Smith et al, 2001" or "Data ref: NCBI Sequence Read Archive PRJNA342805, 2017". In the Reference list, data citations must be labeled with "[DATASET]". A data reference must provide the database name, accession number/identifiers and a resolvable link to the landing page from which the data can be accessed at the end of the reference. Further instructions are available at .

12) Please also note our reference format:
.

13) We now use CRediT to specify the contributions of each author in the journal submission system. CRediT replaces the author contribution section, which should be removed from the manuscript. Please use the free text box to provide more detailed descriptions. See also guide to authors:
.

14) As part of the EMBO publications' Transparent Editorial Process, EMBO reports publishes online a Review Process File to accompany accepted manuscripts. This File will be published in conjunction with your paper and will include the referee reports, your point-by-point response and all pertinent correspondence relating to the manuscript.

You can opt out of this by letting the editorial office know (emboreports@embo.org). If you do opt out, the Review Process File link will point to the following statement: "No Review Process File is available with this article, as the authors have chosen not to make the review process public in this case."

We would also welcome the submission of cover suggestions, or motifs to be used by our Graphics Illustrator in designing a cover.

I look forward to seeing a revised version of your manuscript when it is ready. Please let me know if you have any questions or comments regarding the revision.

Best regards,

Ioannis

Ioannis Papaioannou, PhD
Editor
EMBO reports

Referee #1:

The manuscript by Burigotto et al describes a mechanism monitoring mitotic timing in cells prone to chromosome segregation defects. 53BP1 acts as an essential component of this mitotic surveillance pathway (MSP) and it was known to dynamically associate with kinetochores. However, the relevance of this localization pattern for the MSP function remained elusive.
Here, the authors identify CENP-F as a kinetochore receptor for 53BP1 and demonstrate that inhibiting CENP-F-mediated kinetochore recruitment of 53BP1 does not reduce MSP functionality. Instead, they show that removal of 53BP1 from kinetochores can be regulated by PLK1 kinase activity and is important for MSP to be correctly executed. This is an interesting study of good scientific and technical quality and most of the authors' claims are convincingly supported by the presented data. It is also relevant to the mitosis and (potentially to) cancer fields as it aims to elucidate the exact regulatory mechanisms of the MSP. However, several main points and a couple of other issues listed below need to be addressed to render this manuscript acceptable at EMBO Reports.

Major issues:

1. Related to the Figures 1E, F, H; 2E, F and EV2B, C, even though the manipulations of CENP-F (siRNA, E564P mutant) in cells seem not to affect normal growth, the authors should repeat the corresponding kinetochore localization analyses in cells synchronized in one stage of mitosis to exclude any possible differences due to delays in mitotic progression. Since most of the

examples shown are prometaphase stages, the use of Eg5 inhibitor/s could be a good choice.

2. In all examples of kinetochore localization, it is not clear how the signal (AU) was normalized. Was CREST signal used as a reference and data are presented as a ratio?

3. All quantifications do not show statistical significance in the figures and should be added (as it is indicated in the methods section that this analysis has been done).

4. The quantifications are missing for the Figures 1H and EV3A. For the Fig. EV3A the examples of cells shown are not convincingly demonstrating 53BP1-V5 striping from kinetochores and should be replaced by better ones. In the quantifications shown in Fig. EV3B, the corresponding pictures of cells are missing.

5. In the quantification shown in Fig. 4A and B, the corresponding pictures of cells are missing.

Minor issues

1. The generation of PLK1-AS and CENP-F E564P double mutant cells (Fig. 4C) should be mentioned in the text and explained in the methods section.

2. Fig EV1 is wrongly annotated as EV2 on the figure shown on the page 50.

3. It would be interesting to see authors' speculation in the discussion section on how PLK1-mediated phosphorylation events could inhibit loss of 53BP1 from kinetochores. For instance, could the reported or predicted PLK1-phosphorylated residues on CENP-F and/or 53BP1 indicate a possible mechanism?

Referee #2:

In their manuscript, the authors have established endogenously tagged 53BP1 cells, and establish CENPF as a docking site for 53bp1 at kinetochores during mitosis. However, the authors do not convincingly show a phenotype associated with a loss of 53bp1 localization at kinetochores in cells in which the identified docking site is mutated. Although the evidence for the identified docking site is solid, it remains unclear what the role of 53bp1 at kinetochores is, and the relevance of this finding is therefore limited.

In a second part, Plk1 is identified as a kinase that displaces 53BP1 from kinetochores. Although the amount of data is limited, Plk1 inhibition and use of an analogue sensitive allele convincingly show that PLK1 inhibition leads to 53BP1 retention on kinetochores. This is associated with a phenotype, but since PLK1 has many roles, it cannot be concluded that the observed phenotype is related to PLK1-mediated 53bp1 dynamics at kinetochores. To test this, a phospho-site mutant lacking Plk1 sites in 53bp1 should be made, and phenotypes should be tested in such a context.

Furthermore, I am not convinced by the narrative that the observed phenotypes are specific to a pathway the authors refer to as MSP. P53 activation happens in response to many insults, and this effect is 53BP1 dependent (see also comment below). 53bp1p-/- cells have a defect in p53 activation (expected, but not per se due to duration in mitosis in my view). Many statements are over-interpretation of the data.

Combined, the key finding of the manuscript is that CENPF is the docking site of 53BP1 in mitosis, but a role for this localization has not been identified. In my view some phenotypic effect that can be specifically attributed to 53bp1 localization at kinetochores is required to justify publication.

Comments:

1. The amount of the data is limited, some of the figure panels could be left out of combined with other figures.

2. Abstract: the abstract is written in a dogmatic way, as if the 'mitotic surveillance pathway' is a highly accepted pathway. In my opinion it is not, I would stay away from using MSP in this way, it is not a commonly used term and is easily confused with the spindle assembly checkpoint which also safeguards mitotic progression. Moreover, the mitotic surveillance pathway is mostly studied in a very specific context, centrosome depletion or kinesin-mediated mitotic delay. Also, generic p53 activation is used as a measure of MSP, and p21 upregulation is used as 'senescence'. This is a gross overinterpretation of results.

3. Figure 1: CENF depletion: could it be that CENPF depletion leads to disruption of the kinetochore? CENPA localization appears normal, but are the timing of mitosis, chromosome segregation, APC/C activation affected?

4. Figure 1G/H are hardly described. Could this data be accompanied by images and explanation? Is it surprising that CENPF depletion does not lead to effects? Does CENPF depletion lead to missegregating chromosomes?

5. Figure 2A can be left out. A screenshot from public data. The text in the results section suffices.

6. Figure 2: the mutant of CENPF is very nice, but the amount of the data is very limited. Fig 2 and 3 could easily be combined.

7. Figure 2F: appears to be n=1, if true, than biological replicates are needed. Same for Figure 4A/B: appears to be n=1. Replicates and statistics are required.

8. Page 2: 'Thus, we conclude that although not sufficient to fully activate the MSP, 53BP1 release from KTs is an essential step in MSP activation.' This conclusion is based on experiments with Plk1 inhibition, which acts on many pathways and substrates. This conclusion cannot be made based on the results.

9. Figure 3/b and 3D/E: Centrinone treatment leads to a p53 response, which limits clonogenic outgrowth. 53pb1 inactivation prevents a centrinone-induced p53 response. This phenotype is not specific for centrinone. Nutlin-induced p53 stabilization and cell cycle arrest, for instance, can also be prevented by 53Bp1 inactivation (Brummelkamp et al, Nature Chem Bio, 2006). The authors use 53BP1 -/- as a positive control for MSP deficiency. This is a stretch, since many more aspects of 53bp1 are affected (including DNA repair), and the fact that 53BP1-/- cells just have a generic p53 defect.

10. Same comment as above, but on the experiment with the nocodazole treatment: how is this related to MSP? In my perception, this again is a generic p53 response, that is dependent on 53BP1, and CENPF has no role in it.

11. Figure 4A: kinases involved in 53BP1 loading: there are no effects when kinase inhibitors are applied for 20 minutes. It could well be that 20 minutes incubation is too short to see effects. It is essential to use 6h, like in the assay in figure 4, to reassure that the inhibitors are applied in a setting where effects can be picked up.

12. Figure 5D/E: Plk1 inhibition restores clonogenic potential. This assay is hard to interpret. The conclusion:'Strikingly, PLK1 inhibition restored cell cycle re-entry in WT cells, demonstrating that PLK1 activity during extended prometaphase is crucial for MSP function (Fig. 4D-E)'. The conclusion is not correct, because PLK1 has many roles and substrates, and how this assays is

a readout for MSP function is unclear to me.

13. Some textual issues:

Page 2: acidi ->acid

Page 5: likewise -> like

Referee #3:

Review of EMBO Reports manuscript 57234V1 entitled "The mitotic surveillance pathway requires PLK1-dependent 53BP1 displacement from kinetochores" by Luca L. Fava and coworkers

The mitotic surveillance pathway or mitotic stopwatch pathway (MSP) is a biological reaktive clock that measures the time spent in mitosis and terminally arrests cells in the subsequent G1 phase if this time was abnormally long. Since an abnormally long mitosis is likely to result in chromosome segregation errors, the MSP is believed to constitute a metazoan cancer protection mechanism. While it is clear that a tp53-53BP1-Usp28 complex is required for MSP and that 53BP1 localizes to outer kinetochores in early mitosis, exactly how time in mitosis is measured remains enigmatic.

In the manuscript at hand, Burigotto et al. report the following key findings: i) Plk1-dependent displacement of 53BP1 from kinetochores is required for MSP. ii) CENP-F constitutes the anchor of 53BP1 at kinetochores. iii) Preventing kinetochore recruitment of 53BP1 by depletion or mutation of CENP-F does not lower the MSP threshold to shorter mitotic duration, as one might expect, but instead renders Plk1 dispensable for MSP. This indicated that the eviction of 53BP1 from kinetochores is not (or at least not only) the timer of mitotic duration and that an independent "cytosolic clock" must exist.

Although this study falls short in unraveling the crucial MSP clock, it nevertheless advances our understanding of this fascinating pathway. Moreover, the author's conclusions are fully supported by clear-cut data. This is a straightforward body of work and I have only few minor points of criticism as outlined below.

1) The MSP threshold assay (Fig. 1G) needs to be explained in the results & discussion section. At least briefly.
2) CENP-F is identified as an interaction partner of 53BP1 by IP from nocodazole-arrested cells. Yet, as pointed out by the authors, 53BP1 disappears from kinetochores in prometaphase - even in presence of nocodazole. The authors might want to meet this seeming contradiction by saying that 10 h after release from thymidine RPE1 cells have only just entered mitosis.
3) The authors cite studies that report the dynein-dependent stripping of CENP-F and 53BP1 from outer kinetochores. Why then is Plk1 essential for displacing 53BP1? This point should be addressed!
4) Likewise, the authors should speculate about how Plk1-dependent phosphorylation might result in disassembly of the CENP-F-53BP1 interaction. Are there any consensus Plk1 phorphorylation sites within the regions of CENP-F and/or 53BP1 that are required for their interaction?
5) The sentence "Strikingly, when PLK1 inhibition was performed in cells in which 53BP1 loading to KT was prevented due to the CENP-FE564P mutation, the MSP override observed in WT cells was also abrogated (Fig. 4D-E)." is not very intuitive. Similarly, the model in figure 5 is not easily accessible to the layman reader. The manuscript could be improved in conveying this key conclusion.
6) Typo at the end of the introduction: "amino acidi substitution"

Referee #4:

This paper from Burigotto and colleagues sets out to investigate the mitotic surveillance pathway (MSP) and how kinetochores may play an important role. This is motivated by the finding that 53BP1 is, 1) required for the MSP independently of its role in the DNA damage response, and 2) transiently associates with kinetochores in early mitosis. The MSP is defined as a mechanism that arrests cells in the G1 phase of the cell cycle if the duration a cell spends in mitosis exceeds a certain threshold or there is a mitotic disturbance. This requires the formation of a ternary complex of 53BP1, USP28 and p53, however the underlying damage signals are unknown and how this is mechanistically linked to the mitotic duration is also unknown. Regarding the latter, Fong et al, 2016 proposed the idea that delocalisation of 53BP1 from kinetochores may directly activates G1 arrest. Thus the question being addressed here is important as it could provide new insight into the underlying mechanism of the MSP.

The authors identify CENP-F as the kinetochore-receptor for 53BP1. My mapping the interaction site, and making a mutant that

abrogates binding, the authors directly tested whether 53BP1 recruitment to kinetochores is required for the MSP (it would be good to see this interaction reconstituted with purified proteins/fragments). The outcome is that kinetochore localisation is dispensable for MSP. This is an important result and I think it is important that this, albeit negative result, is reported. In this regard I think the current title is inappropriate as it gives the impression that kinetochores are required for the MSP. This is not necessarily the case.

The title is rooted in the series of experiments that seek to show how PLK1 activity is needed for the G1 arrest and that this effect is abolished when 53BP1 cannot bind kinetochores. To this reviewer the experiment suggests that it is free cytoplasmic 53BP1 that is important for a functional MSP. Can the authors show that this is the case? is it teh accumlation fo cytoplasmic protein (compelxes) that sets the threshold for teh MSP int he following interphase? Crucially, the kinetochores role in this process is only seen if 53BP1 is forced to stay on kinetochores (by inhibiting PLK1). i.e. it is a gain-of-function phenotype and not necessarily a physiological event during a normal cell cycle. This should be clearerly stated in the text.

In fact, 53BP1 is lost from kinetochores in a normal mitosis as microtubule-kinetochore attachments form. Is this process PLK1 dependent? The authors do not show if this loss is delayed/abolished when PLK1 is inhibited, they only show that 53BP1 is kinetochore bound after 16 h nocodazole treatment. Fig EV3B goes in this direction but it is not stated what mitotic phase the async population are in. Much more work is needed here to understand the role of PLK1.

PLK1 is also implicated in SAC maintenance, mitotic exit and centrosome function. The authors should address how this might effect interpretation of the data. For example, what is the fate of mitotic cells under the various conditions described? For this live cell imaging of WT and mutant cells in presence and absence of PLK1 activity of essential.

As this stands I feel that there is not enough to convince me that PLK1 mediated stripping of 53BP1 from kinetochores is a key part of the MSP mechanism in a normal mitosis.

I do also have some comments about quality of the data and analysis:

1. The immunofluorescence and microscopy are not of a high quality. The microscopes described in methods look appropriate so I have concerns about the signal-to-noise in the experiments. This is important as the quantifications are key to interpretation of results (especially Fig 4).
2. The methods state that single kinetochore measurements were made on maximum intensity projections. I don't think this is appropriate as you are potentially merging kinetochores that are in the same x,y position but different z.
3. There are no statistical analysis presented. This is not acceptable and makes it impossible to interpret the data.

Point-by-point response to Reviewers' comments (our responses are in blue font)

Firstly, we would like to thank the editor and all four reviewers for their time and commitment to strengthen our manuscript. By addressing all key reviewer's points, we have improved the soundness of our work and, at the same time, broadened the scope of our manuscript. We hope that our work will now match the standards for publication of the EMBO Reports. Please find our point-by-point rebuttal below. The textual changes in our manuscript have been highlighted in yellow.

Referee #1:

The manuscript by Burigotto et al describes a mechanism monitoring mitotic timing in cells prone to chromosome segregation defects. 53BP1 acts as an essential component of this mitotic surveillance pathway (MSP) and it was known to dynamically associate with kinetochores. However, the relevance of this localization pattern for the MSP function remained elusive.
Here, the authors identify CENP-F as a kinetochore receptor for 53BP1 and demonstrate that inhibiting CENP-F-mediated kinetochore recruitment of 53BP1 does not reduce MSP functionality. Instead, they show that removal of 53BP1 from kinetochores can be regulated by PLK1 kinase activity and is important for MSP to be correctly executed. This is an interesting study of good scientific and technical quality and most of the authors' claims are convincingly supported by the presented data. It is also relevant to the mitosis and (potentially to) cancer fields as it aims to elucidate the exact regulatory mechanisms of the MSP. However, several main points and a couple of other issues listed below need to be addressed to render this manuscript acceptable at EMBO Reports.

We thank this reviewer for acknowledging the relevance of our study as well as its technical robustness.

Major issues:

1. Related to the Figures 1E, F, H; 2E, F and EV2B, C, even though the manipulations of CENP-F (siRNA, E564P mutant) in cells seem not to affect normal growth, the authors should repeat the corresponding kinetochore localization analyses in cells synchronized in one stage of mitosis to exclude any possible differences due to delays in mitotic progression. Since most of the examples shown are prometaphase stages, the use of Eg5 inhibitor/s could be a good choice.

Thanks for raising our attention on the possible impact of CENP-F^{E564P} mutation on mitotic progression. To experimentally tackle this criticism, we performed live imaging on WT and CENP-F^{E564P} cells undergoing both unperturbed mitotic traverse as well as mitosis in the presence of high concentrations of nocodazole (to assess spindle checkpoint proficiency). Neither normal mitotic timing nor spindle checkpoint proficiency were affected by the CENP-F^{E564P} mutation, demonstrating thereby that phenotypes caused by CENP-F^{E564P} mutation cannot be ascribed to altered mitotic progression (Appendix Fig. S3A-C and Movie EV1).

Importantly, a large body of new work relying on Eg5 inhibition has also been included in the revised version (Fig. EV4B, Appendix Fig. S8), providing an additional line of evidence that altered 53BP1 kinetochore localization in CENP-F^{E564P} cells does not reflect a delay in mitotic progression.

2. In all examples of kinetochore localization, it is not clear how the signal (AU) was normalized. Was CREST signal used as a reference and data are presented as a ratio?

All KT fluorescence measurements were calculated as: mean grey value inside KT ROI minus mean grey value inside cellular background ROI. All values were then normalised dividing each KT value by the mean of the values in the control sample (WT untreated, WT asynchronous or WT “M”, depending on the specific experiment and now clearly stated in every figure legend). We used CREST signal to guide the positioning of all ROIs. However, we decided not to express KT values as a ratio on CREST signal because we noticed that: i) our CREST signal showed high variability between cells of the same samples and between different samples; ii) this variability was not paralleled by a different intensity of the other KT signals (e.g. 53BP1). In Rebuttal Figure 1 you can find an example of a representative quantification of 53BP1 fluorescence signal at individual KTs, expressed either as 53BP1 absolute value (left panel) or as a ratio to CREST signal (right panel), along with the values of CREST signal alone (middle panel).

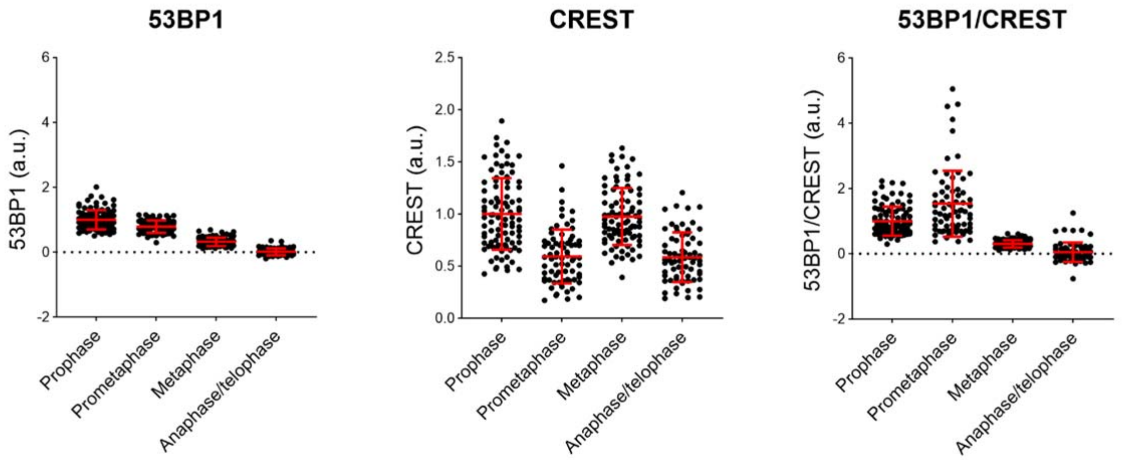

Rebuttal Figure 1. Dot plots showing the intensity of 53BP1 fluorescence (left panel) and CREST (middle panel) at individual KTs, expressed as absolute value. The right panel shows the ratio between 53BP1 and CREST fluorescence signals inside the same ROI. Mean values (red lines) ± SD are reported, normalized on the Prophase sample.

3. All quantifications do not show statistical significance in the figures and should be added (as it is indicated in the methods section that this analysis has been done).

We have now uniformly performed statistics for all figure panels in which image quantifications were performed. We have also compiled a table summarizing sample numerosity and the statistical tests performed (Table EV3).

4. The quantifications are missing for the Figures 1H and EV3A. For the Fig. EV3A the examples of cells shown are not convincingly demonstrating 53BP1-V5 striping from kinetochores and should be replaced by better ones. In the quantifications shown in Fig. EV3B, the corresponding pictures of cells are missing.

Regarding former Fig. EV3A (the 53BP1 stripping assay), we have repeated the experiment and acquired better images on a laser scanning confocal microscope (shown in Fig. EV3A). To provide an unbiased quantification of the data, a dedicated image segmentation strategy has been designed (Appendix Fig. S5), yielding the quantification requested by this reviewer (Fig. EV3B).
The former EV3B panel is now shown in Fig. EV3D; the corresponding representative pictures have now been included in Fig. EV3C, as requested.
Finally, the siRNA data in RPE1 cells have been moved Appendix Fig. S4A-B and the quantification of CENP-F knockdown efficiency is reported in Appendix Fig. S4C.

5. In the quantification shown in Fig. 4A and B, the corresponding pictures of cells are missing.

Revised versions of Fig. 4A and B are now shown in Fig. 3A and 3B, respectively. The corresponding representative pictures have been included in Appendix Fig. S6 and Fig. EV4A, respectively.

Minor issues

1. The generation of PLK1-AS and CENP-F E564P double mutant cells (Fig. 4C) should be mentioned in the text and explained in the methods section.

We have now explicitly referred to the generation of this mutant, both in the main text (line 236-237) and methods (line 355-356).

2. Fig EV1 is wrongly annotated as EV2 on the figure shown on the page 50.

We thank this reviewer for spotting this mistake that has now been corrected.

3. It would be interesting to see authors' speculation in the discussion section on how PLK1-mediated phosphorylation events could inhibit loss of 53BP1 from kinetochores. For instance, could the reported or predicted PLK1-phosphorylated residues on CENP-F and/or 53BP1 indicate a possible mechanism?

In light of specific criticisms raised by Referee #2, we went beyond speculating about the mechanism exploited by PLK1 to modulate the 53BP1:CENP-F association at KTs. In fact, we generated a 13 Ser/Thr-Ala mutant preventing 53BP1 phosphorylation at all 13 possible PLK1 consensus sites, some of which were experimentally validated before. Strikingly, this 53BP1 mutant retained the ability to undergo PLK1-dependent loss of KT affinity (Fig. EV5A and Appendix Fig. S9), demonstrating that 53BP1 phosphorylation by PLK1 is not sufficient to account for 53BP1 dissociation from KTs. In our revised discussion (lines 273-278) we speculate that CENP-F phosphorylation by PLK1 might account for the observed dynamic localization. While CENP-F is a well-established PLK1 substrate (Santamaria et al, 2010), there are neither known PLK1 phospho-sites nor PLK1 consensus sites near the 53BP1 interaction interface we mapped by yeast-two-hybrid. Thus, as the CENP-F structure is unknown and AlphaFold does not correctly predict the overall tertiary (and potentially quaternary) structure of the protein, we can only speculate that PLK1 phosphorylation allosterically impacts on the propensity of CENP-F to interact with 53BP1, e.g., masking and unmasking the 53BP1 binding site. Please note that CENP-F is a polypeptide of 3.114 aminoacidic residues, thus establishing a robust add-back strategy (e.g., relying on lentiviral expression) is not feasible.

Referee #2:

In their manuscript, the authors have established endogenously tagged 53BP1 cells, and establish CENPF as a docking site for 53bp1 at kinetochores during mitosis. However, the authors do not convincingly show a phenotype associated with a loss of 53bp1 localization at kinetochores in cells in which the identified docking site is mutated. Although the evidence for the identified docking site is solid, it remains unclear what the role of 53bp1 at kinetochores is, and the relevance of this finding is therefore limited.

We thank this reviewer for being frank about his/her scepticism regarding the relevance of the first part of our study, as this gives us a chance to better explain our perspective.
53BP1 localization at mitotic KTs is known since more than two decades (Jullien et al, 2002) without any associated function. One of the seminal studies demonstrating that 53BP1 contributes to relaying mitotic delay to p53 postulates a central role of the KT localization of 53BP1 for this function (Fong et al, 2016). Considering that this 2016 paper has been cited 169 times as of today, it can be readily appreciated that the surprising notion that 53BP1 activity does not originate at KTs, but it rather stems from the mitotic cytosol, is not of limited relevance (please note that Referee #4 contextualizes our findings exactly in the same manner).

Concretely speaking, we have not only improved the writing of our manuscript based on this reviewer's input, but we have also substantially strengthened the second part of the paper (discussed below). Perturbation of the CENP-F docking capability remains central to our endeavour.

In a second part, Plk1 is identified as a kinase that displaces 53BP1 from kinetochores. Although the amount of data is limited, Plk1 inhibition and use of an analogue sensitive allele convincingly show that PLK1 inhibition leads to 53BP1 retention on kinetochores. This is associated with a phenotype, but since PLK1 has many roles, it cannot be concluded that the observed phenotype is related to PLK1-mediated 53bp1 dynamics at kinetochores. To test this, a phospho-site mutant lacking Plk1 sites in 53bp1 should be made, and phenotypes should be tested in such a context.

As detailed below, we have performed the requested experiment (Fig. EV5A and Appendix Fig. S9). Strikingly, 53BP1 devoid of PLK1 phosphorylation still retained its ability to undergo PLK1-dependent loss of KT affinity. In our view, this does not diminish the importance of PLK1-mediated 53BP1 dynamics at KTs, but rather shows that 53BP1 is not the (only) relevant PLK1 substrate in this context.
Moreover, we extended our findings by demonstrating that PLK1-dependent displacement of 53BP1 from KTs is a prerequisite for its association with p53 protein in the mitotic cytosol (Fig. 4A and EV5B). It is hard to postulate that this phenotype is not dependent on the control of 53BP1 KT dynamics by PLK1, as mutation of the CENP-F docking site restores the ability of 53BP1 to bind p53 even in the absence of PLK1 activity (Fig. 4A and EV5B).

Furthermore, I am not convinced by the narrative that the observed phenotypes are specific to a pathway the authors refer to as MSP. P53 activation happens in response to many insults, and this effect is 53BP1 dependent (see also comment below). 53bp1p-/-

cells have a defect in p53 activation (expected, but not per se due to duration in mitosis in my view). Many statements are over-interpretation of the data.

While thanks to this reviewer's comments we improved our writing that is now more rigorous in several circumstances, we respectfully disagree with the idea that only one "general" p53 response exists, devoted to sense a "general" form of stress (our view is explained in greater detail below). We hope that by referring to specific studies that have addressed the "particular" p53 stress response investigated by our work (named "mitotic surveillance pathway" before) we will convince this reviewer that our revised narrative is appropriate to contextualize the experimental data presented in our study.

Combined, the key finding of the manuscript is that CENPF is the docking site of 53BP1 in mitosis, but a role for this localization has not been identified. In my view some phenotypic effect that can be specifically attributed to 53bp1 localization at kinetochores is required to justify publication.

Considering that our manuscript grew both in terms of experimental data as well as its narrative, we hope that this reviewer will now acknowledge both the importance and the soundness of our revised study.

Comments:

1. The amount of the data is limited, some of the figure panels could be left out of combined with other figures.

We have now merged former Fig. 1 and 2 in one individual figure. Several panels originally displayed in main figures that were deemed of limited relevance by this reviewer are now displayed either in EV figures or in Appendix.

2. Abstract: the abstract is written in a dogmatic way, as if the 'mitotic surveillance pathway' is a highly accepted pathway. In my opinion it is not, I would stay away from using MSP in this way, it is not a commonly used term and is easily confused with the spindle assembly checkpoint which also safeguards mitotic progression. Moreover, the mitotic surveillance pathway is mostly studied in a very specific context, centrosome depletion or kinesin-mediated mitotic delay. Also, generic p53 activation is used as a measure of MSP, and p21 upregulation is used as 'senescence'. This is a gross overinterpretation of results.

We recognize the inappropriate use of the term 'senescence' to describe p21 upregulation. We thank this reviewer for pointing out this mistake. We have now changed this to "p21-dependent cell cycle arrest" (line 198-199).
About the remaining criticism, there are two lines of argumentations that we want to put forward:

- firstly, we are firm about the fact that p53 activation in response to extended mitotic timing (caused by either chronic centriole depletion or various trigger of mitotic delay, i.e., defects in spindle maturation, microtubule dynamics or APC/C activation dynamics) is a dedicated p53 response with distinct genetic requirements when compared to the "canonical" DNA damage response. To this end, mitotic delay

triggers p53 activation relying on 53BP1 and USP28 while ATM, ATR, RNF8, Chk1 and Chk2 are dispensable for this kind of p53 activation. Such notions stem both from solid work relying on tissue culture models (Fong et al, 2016; Lambrus et al, 2016; Meitinger et al, 2016) as well as from in vivo ablation of centrosomes (Marjanović et al, 2015; Phan et al, 2021);

- secondly, as we are dealing with a specific p53 response that has been solidly established by others and is not uncovered here, we simply face the need to refer to this pathway in a concise manner. The definition of "mitotic surveillance pathway" has been put forward in (Lambrus & Holland, 2017) and since then several studies referred to this pathway in this way, including EMBO Reports and EMBO Journal articles exploiting this terminology in their title (Phan et al, 2021; Xiao et al, 2021). We are convinced that introducing a novel terminology here will cause even more confusion that the one this reviewer is referring to. On the other hand, describing the p53 response to mitotic delay as a general one would not be accurately reflecting the state of the art in the p53 field.

3. Figure 1: CENF depletion: could it be that CENPF depletion leads to disruption of the kinetochore? CENPA localization appears normal, but are the timing of mitosis, chromosome segregation, APC/C activation affected?

We imaged WT and CENP-F^{E564P} cells undergoing both unperturbed mitotic traverse as well as mitosis in the presence of high concentrations of nocodazole. Neither normal mitotic timing, nor the presence of lagging chromosomes in anaphase, nor the time to undergo mitotic slippage in the presence of nocodazole were affected by the CENP-F^{E564P} mutation, demonstrating thereby that no phenotype caused by the CENP-F^{E564P} mutation can be ascribed to altered mitotic progression or fidelity of this mutant (Appendix Fig. S3A-B and Movie EV1). The fact that the time required for mitotic slippage in the presence of nocodazole was not affected suggests instead that APC/C activation and spindle checkpoint proficiency are also unaltered (Appendix Fig. S3C).

4. Figure 1G/H are hardly described. Could this data be accompanied by images and explanation? Is it surprising that CENPF depletion does not lead to effects? Does CENPF depletion lead to missegregating chromosomes?

Thanks for raising this criticism. As we strengthened the analysis of our CENP-F^{E564P} mutant regarding the absence of chromosomal missegregation (Appendix Fig. S3A-B and Movie EV1), we decided to repeat this assay in the CENP-F^{WT} and CENP-F^{E564P} cells, reaching a similar conclusion (i.e., no substantial difference between genotypes). The new data are now shown in Fig. 2F and the assay is better described (lines 198-200). Moreover, the corresponding movie stills are displayed in Appendix Fig. S44D.
Regarding the old siRNA data, they have instead been moved to Appendix Fig. S4A-C as an additional line of evidence supporting the notion of an absence of CENP-F contribution to the timer (lines 200-202).

5. Figure 2A can be left out. A screenshot from public data. The text in the results section suffices.

The presented data are not screenshots. We generated the figure ourselves utilizing publicly available data, appropriately referencing the source. Considering that the

distribution of the datapoints displayed in the graphics reveals much more information than our text can do, we moved the panel to Fig. EV2A rather than completely removing it.

6. Figure 2: the mutant of CENPF is very nice, but the amount of the data is very limited. Fig 2 and 3 could easily be combined.

Fig. 2 has been merged with Fig. 1. Fig. 3 instead conveys a message that is conceptually distinct, and thus we prefer to keep it separate.

7. Figure 2F: appears to be n=1, if true, than biological replicates are needed. Same for Figure 4A/B: appears to be n=1. Replicates and statistics are required.

New independent biological replicates have been performed for the requested experiments. In addition to that, all new experiments have been reproduced multiple times and statistics has been performed throughout the manuscript. The number of cells analysed, the number of replicates and the statistical test used are now reported in Table EV3.

8. Page 2: 'Thus, we conclude that although not sufficient to fully activate the MSP, 53BP1 release from KTs is an essential step in MSP activation.' This conclusion is based on experiments with Plk1 inhibition, which acts on many pathways and substrates. This conclusion cannot be made based on the results.

Clonogenicity is simple to measure yet its variations can stem from alteration of a multitude of different processes. On the other hand, PLK1 is highly pleiotropic, leaving room for other alternative explanations regarding its contribution to the observed phenotype.
To experimentally tackle this important criticism, we set out to look for a readout of MSP activation that is:

a. Compatible with single cell measurement
b. Assessable in the same mitosis in which PLK1 is being inhibited.

To this end, we inferred 53BP1 binding to p53 by performing proximity ligation assays (PLA).
Strikingly, PLK1 inhibition not only promoted 53BP1 KT retention but also prevented its binding to p53 in the mitotic cytosol (Fig. 4A and EV5B). Genetically interfering with 53BP1 docking at KTs (CENP-F^{E564P}) was sufficient to restore 53BP1 capability to bind to p53 in the mitotic cytosol, even in the absence of PLK1 activity (Fig. 4A and EV5B).
Thus, we provide a direct measure of MSP activation in individual mitotic cells, we confirm the involvement of PLK1 to MSP activation and we extend these observations by demonstrating that preventing 53BP1 KT localization is sufficient to abolish the PLK1 contribution to the pathway.
It is important to mention that the PLA signals measured in our study likely reflect the assembly of a protein complex comprising not only 53BP1 and p53, but also USP28, as demonstrated in complementary immunoprecipitation experiments performed by Franz Meitinger, Arshad Deasi, Karen Oegema and colleagues in a recent preprint (Fig. 3 of (Meitinger et al, 2022)). While their study demonstrates that the MSP time threshold and MSP protein complex assembly critically rely on PLK1, it offers no explanation on how PLK1 might impact on the described phenomena, strengthening thereby the relevance of our paper. We sincerely hope that this reviewer will now agree with this view.

To better emphasize the data discussed here as the main finding of our paper, we changed the title of our manuscript to:

"PLK1 promotes the mitotic surveillance pathway by controlling 53BP1 cytosolic availability"

and the running title to

"The mitotic cytosol controls the MSP"

9. Figure 3/b and 3D/E: Centrinone treatment leads to a p53 response, which limits clonogenic outgrowth. 53pb1 inactivation prevents a centrinone-induced p53 response. This phenotype is not specific for centrinone. Nutlin-induced p53 stabilization and cell cycle arrest, for instance, can also be prevented by 53Bp1 inactivation (Brummelkamp et al, Nature Chem Bio, 2006). The authors use 53BP1 -/- as a positive control for MSP deficiency. This is a stretch, since many more aspects of 53bp1 are affected (including DNA repair), and the fact that 53BP1-/- cells just have a generic p53 defect.

We are aware of the Brummelkamp paper as well as of more recent data demonstrating that not only 53BP1 but also USP28 confer resistance to Nutlin (Cuella-Martin et al, 2016). The two studies demonstrate that 53BP1 knockdown/knock-out PARTIALLY reduce p53 functionality in response to Nutlin. Five different studies instead demonstrate that centriole depletion SOLELY relies on 53BP1 and USP28 to elicit a p53 response, with no measurable residual p53 function in response to this insult when 53BP1 and USP28 were deleted, both in vitro and in vivo (Lambrus et al, 2016; Fong et al, 2016; Meitinger et al, 2016; Phan et al, 2021; Xiao et al, 2021). Thus, we are not establishing this positive control here, it has been readily established in the five abovementioned studies (published by four different research groups).
In addition to our already detailed motivation to refuse the oversimplified concept of a "general" p53 response, we would like to argue that pharmacologic MDM2 inhibition by Nutlin is an artificial mean to activate p53 in the absence of stress, as opposed to the MSP that responds to a specific stressor that can arise during healthy somatic cell divisions or in the presence of centrosomal malfunctions such as those promoted by microcephaly causing mutations. Thus, it is our opinion that partially overlapping genetic requirements for p53 activation in response to Nutlin vs MSP do not invalidate our choice of using 53BP1 KO as positive control for the lack of MSP proficiency.

10. Same comment as above, but on the experiment with the nocodazole treatment: how is this related to MSP? In my perception, this again is a generic p53 response, that is dependent on 53BP1, and CENPF has no role in it.

This point has already been addressed in response to the previous comment.

11. Figure 4A: kinases involved in 53BP1 loading: there are no effects when kinase inhibitors are applied for 20 minutes. It could well be that 20 minutes incubation is too short to see effects. It is essential to use 6h, like in the assay in figure 4, to reassure that the inhibitors are applied in a setting where effects can be picked up.

As requested, we performed the experiment exposing the cells to 6h of kinase inhibition before imaging (Fig. 3A and Appendix Fig. S6). The results we obtained are similar to the original ones, ruling out the possibility that the lack of effects that we originally reported reflects an insufficient kinase inhibition.

12. Figure 5D/E: Plk1 inhibition restores clonogenic potential. This assay is hard to interpret. The conclusion:'Strikingly, PLK1 inhibition restored cell cycle re-entry in WT cells, demonstrating that PLK1 activity during extended prometaphase is crucial for MSP function (Fig. 4D-E)'. The conclusion is not correct, because PLK1 has many roles and substrates, and how this assays is a readout for MSP function is unclear to me.

We have already addressed this criticism in response to point 8 raised by this reviewer. The fact that the formation of 53BP1-p53 complexes measured in Fig. 4A and EV5B is responsible for the differences in clonogenicity remains to be formally established. We have now discussed those data using a more careful language (lines 279-283) and we also explicitly refer to the limitations of our study in the discussion paragraph (289-291). We have also given credit to the Meitinger pre-print that lends independent support to our conclusions (line 289).

13. Some textual issues:

Page 2: acidi ->acid

Page 5: likewise -> like

Thank you, we have corrected accordingly.

Referee #3:

Review of EMBO Reports manuscript 57234V1 entitled "The mitotic surveillance pathway requires PLK1-dependent 53BP1 displacement from kinetochores" by Luca L. Fava and coworkers

The mitotic surveillance pathway or mitotic stopwatch pathway (MSP) is a biological reaktive clock that measures the time spent in mitosis and terminally arrests cells in the subsequent G1 phase if this time was abnormally long. Since an abnormally long mitosis is likely to result in chromosome segregation errors, the MSP is believed to constitute a metazoan cancer protection mechanism. While it is clear that a tp53-53BP1-Usp28 complex is required for MSP and that 53BP1 localizes to outer kinetochores in early mitosis, exactly how time in mitosis is measured remains enigmatic.

In the manuscript at hand, Burigotto et al. report the following key findings: i) Plk1-dependent displacement of 53BP1 from kinetochores is required for MSP. ii) CENP-F constitutes the anchor of 53BP1 at kinetochores. iii) Preventing kinetochore recruitment of 53BP1 by depletion or mutation of CENP-F does not lower the MSP threshold to shorter mitotic duration, as one might expect, but instead renders Plk1 dispensable for MSP. This indicated that the eviction of 53BP1 from kinetochores is not (or at least not only) the timer of mitotic duration and that an independent "cytosolic clock" must exist.

Although this study falls short in unraveling the crucial MSP clock, it nevertheless advances our understanding of this fascinating pathway. Moreover, the author's conclusions are fully supported by clear-cut data. This is a straightforward body of work and I have only few minor points of criticism as outlined below.

Many thanks to this reviewer appreciating strengths and weaknesses of our study in such a balanced fashion.

1) The MSP threshold assay (Fig. 1G) needs to be explained in the results & discussion section. At least briefly.

Please note that the CENP-F siRNA used in former Fig. 1G was raising some concerns regarding a spurious contribution of chromosomal missegregation caused by this treatment (comment nr. 4 by Referee #2). As we now show that the CENP-F^{E564P} mutation has no impact on chromosome segregation fidelity (Appendix Fig. S3B), we have repeated the same assay in the presence of this mutation (Fig. 2F). As the new assay aligns very well with the old one, we kept the old data in Appendix Fig. S4.
The assay is briefly described in lines 197-201.

2) CENP-F is identified as an interaction partner of 53BP1 by IP from nocodazole-arrested cells. Yet, as pointed out by the authors, 53BP1 disappears from kinetochores in prometaphase - even in presence of nocodazole. The authors might want to meet this seeming contradiction by saying that 10 h after release from thymidine RPE1 cells have only just entered mitosis.

Many thanks for suggesting this improvement. This might indeed greatly facilitate the navigation through our data. We have replaced “10 h” with “M”; “12 h”, “14 h” and “16 h” after thymidine release were re-named as “M + 2h”, “M + 4h” and “M+ 6h”, respectively in our figure panels (Fig. 3C, 4A-C, EV3C-D, EV4B, EV5A-B, Appendix Fig. S7-9).

3) The authors cite studies that report the dynein-dependent stripping of CENP-F and 53BP1 from outer kinetochores. Why then is Plk1 essential for displacing 53BP1? This point should be addressed!

We have addressed this point experimentally by demonstrating that PLK1 is required for 53BP1 KT removal not only in the absence of microtubules (nocodazole) but also in their presence (Eg5 inhibition, a condition that does not prevent dynein dependent “stripping” (Auckland et al, 2020)). The new data are displayed in Fig. EV4B and Appendix Fig. S8. Thus, while stripping might be an important component of 53BP1 removal from KTs in an unperturbed mitosis, in the context of a delayed mitosis 53BP1 is not capable of displaying KT loss of affinity unless promoted by PLK1. This has been discussed accordingly in the manuscript (lines 240-248).

4) Likewise, the authors should speculate about how Plk1-dependent phosphorylation might result in disassembly of the CENP-F-53BP1 interaction. Are there any consensus Plk1 phorphorylation sites within the regions of CENP-F and/or 53BP1 that are required for their interaction?

In light of specific criticisms raised by Referee #2, we went beyond speculating about the mechanism exploited by PLK1 to modulate the 53BP1:CENP-F association at KTs. In fact, we generated a 13 Ser/Thr-Ala mutant preventing 53BP1 phosphorylation at all 13

possible PLK1 consensus sites, some of which were experimentally validated before. Strikingly, this 53BP1 mutant retained the ability to undergo PLK1-dependent loss of KT affinity (Fig. EV5A and Appendix Fig. S9), demonstrating that 53BP1 phosphorylation by PLK1 is not sufficient to account for 53BP1 dissociation from KTs. In our revised discussion (lines 273-278) we speculate that CENP-F phosphorylation by PLK1 might account for the observed dynamic localization. While CENP-F is a well-established PLK1 substrate (Santamaria et al, 2010), there are neither known PLK1 phospho-sites nor PLK1 consensus sites near the 53BP1 interaction interface we mapped by yeast-two-hybrid. Thus, as the CENP-F structure is unknown and AlphaFold does not correctly predict the overall tertiary (and potentially quaternary) structure of the protein, we can only speculate that PLK1 phosphorylation allosterically impacts on the propensity of CENP-F to interact with 53BP1, e.g., masking and unmasking the 53BP1 binding site. Please note that CENP-F is a polypeptide of 3.114 aminoacidic residues, thus establishing a robust add-back strategy (e.g., relying on lentiviral expression) is not feasible.

5) The sentence "Strikingly, when PLK1 inhibition was performed in cells in which 53BP1 loading to KT was prevented due to the CENP-FE564P mutation, the MSP override observed in WT cells was also abrogated (Fig. 4D-E)." is not very intuitive. Similarly, the model in figure 5 is not easily accessible to the layman reader. The manuscript could be improved in conveying this key conclusion.

We have improved both our narrative (see all changes highlighted in yellow throughout the manuscript) as well as our model (Fig. 5), that is now supported by new experimental data.

6) Typo at the end of the introduction: "amino acidi substitution"

Thank you, we have corrected this typo.

Referee #4:

This paper from Burigotto and colleagues sets out to investigate the mitotic surveillance pathway (MSP) and how kinetochores may play an important role. This is motivated by the finding that 53BP1 is, 1) required for the MSP independently of its role in the DNA damage response, and 2) transiently associates with kinetochores in early mitosis. The MSP is defined as a mechanism that arrests cells in the G1 phase of the cell cycle if the duration a cell spends in mitosis exceeds a certain threshold or there is a mitotic disturbance. This requires the formation of a ternary complex of 53BP1, USP28 and p53, however the underlying damage signals are unknown and how this is mechanistically linked to the mitotic duration is also unknown. Regarding the latter, Fong et al, 2016 proposed the idea that delocalisation of 53BP1 from kinetochores may directly activates G1 arrest. Thus the question being addressed here is important as it could provide new insight into the underlying mechanism of the MSP.

The authors identify CENP-F as the kinetochore-receptor for 53BP1. My mapping the interaction site, and making a mutant that abrogates binding, the authors directly tested whether 53BP1 recruitment to kinetochores is required for the MSP (it would be good to see this interaction reconstituted with purified proteins/fragments). The outcome is that kinetochore localisation is dispensable for MSP. This is an important result and I think it is important that this, albeit negative result, is reported. In this regard I think the current title is inappropriate as it gives the impression that kinetochores are required for the MSP. This is not necessarily the case.

We agree with this reviewer and thank him/her for this constructive point. We have now changed the title of our manuscript to:
"PLK1 promotes the mitotic surveillance pathway by controlling 53BP1 cytosolic availability" and the running title to: "The mitotic cytosol controls the MSP".

The title is rooted in the series of experiments that seek to show how PLK1 activity is needed for the G1 arrest and that this effect is abolished when 53BP1 cannot bind kinetochores. To this reviewer the experiment suggests that it is free cytoplasmic 53BP1 that is important for a functional MSP. Can the authors show that this is the case? is it teh accumlation fo cytoplasmic protein (compelxes) that sets the threshold for teh MSP int he following interphase? Crucially, the kinetochores role in this process is only seen if 53BP1 is forced to stay on kinetochores (by inhibiting PLK1). i.e. it is a gain-of-function phenotype and not necessarily a physiological event during a normal cell cycle. This should be clearerly stated in the text.

We acknowledge the relevance of this point, and we agree that it would be insightful to highlight cytosolic protein complexes. To experimentally tackle this issue, we took advantage of proximity ligation assays (PLAs) to visualize 53BP1 association with p53 in the mitotic cytosol. Strikingly, 53BP1-p53 PLA signals accumulate over time during extended prometaphase while their accumulation is abolished when 53BP1 is forced to stay at KTs by PLK1 inhibition. Crucially, our data demonstrate that PLK1 exerts this effect by controlling 53BP1 KT localization, as PLK1 inhibition in a CENP-F^{E564P} context fails to prevent the accumulation of cytosolic complexes.
We now openly discuss the limitations of our study in the revised discussion. Among them, we still ignore the physiological role of 53BP1 KT localization in a normal mitosis and we clearly acknowledge that the inhibitory contribution of the KT to the MSP can only be highlighted in artificial conditions (i.e., PLK1 inhibition), lines 289-292. We also speculate that a short time window in which 53BP1 is unavailable for the formation of cytosolic protein complexes might concur to the generation of a robust timer threshold (lines 293-296).

In fact, 53BP1 is lost from kinetochores in a normal mitosis as microtubule-kinetochore attachments form. Is this process PLK1 dependent? The authors do not show if this loss is delayed/abolished when PLK1 is inhibited, they only show that 53BP1 is kinetochore bound after 16 h nocodazole treatment. Fig EV3B goes in this direction but it is not stated what mitotic phase the async population are in. Much more work is needed here to understand the role of PLK1.

We agree with this reviewer that our original manuscript was not thorough in addressing the contribution of PLK1 in 53BP1 removal from kinetochores over time, particularly in conditions in which microtubules are not directly perturbed. To this end, we monitored 53BP1 KT localization in cells exposed to increasingly prolonged prometaphase arrest caused not only by nocodazole (Fig. 3C and Appendix Fig. S7), but also by Eg5 inhibition (Fig. EV4B and Appendix Fig. S8). Also in this latter condition, PLK1 activity appeared essential for the time-dependent loss of affinity of 53BP1 for the KT.

PLK1 is also implicated in SAC maintenance, mitotic exit and centrosome function. The authors should address how this might effect interpretation of the data. For example, what is the fate of mitotic cells under the various conditions described? For this live cell imaging of WT and mutant cells in presence and absence of PLK1 activity of essential.

Assessing 53BP1 binding to p53 by PLA (Fig. 4A and EV5B) enabled us to directly monitor the contribution of PLK1 within the same mitotic division in which the kinase was inhibited. This allowed us to readily rule out the possibility that PLK1 indirectly contributed to the above-mentioned binding phenotype by favouring mitotic slippage, by altering mitotic exit or centrosome function. Please note that also our clonogenic assays are performed by selective harvest of mitotic cells by shake off (a very robust procedure), suggesting that also this specific read out is not impacted by the promotion of mitotic slippage upon PLK1 inhibition.
As for live imaging, accurate measurement of the time threshold for MSP activation –/+ PLK1 activity has been performed in a complementary preprint by Franz Meitinger, Arshad Deasi, Karen Oegema and colleagues (Fig. 3 of (Meitinger et al, 2022)). Considering that performing live imaging here would further postpone the publication of our work and will likely duplicate the endeavour of Meitinger and colleagues, we hope that this reviewer will agree that re-assessing MSP functionality -/+ PLK1 inhibition by live imaging will fall outside the timeframe of this revision.

As this stands I feel that there is not enough to convince me that PLK1 mediated stripping of 53BP1 from kinetochores is a key part of the MSP mechanism in a normal mitosis.

We hope that we could now convince this reviewer that in normal mitosis cells undergo anaphase before sufficient MSP protein complexes accumulate (a conclusion that is also supported by the pre-print by (Meitinger et al, 2022) by IP experiments). In an extended mitosis instead PLK1 represents a prerequisite for the MSP, controlling the cytosolic availability of 53BP1 that becomes available for p53 interaction in the mitotic cytosol. The molecular nature of the cytosolic “stopwatch” remains to be established, as this requires neither KTs nor PLK1.
We have now substantially improved the writing of our manuscript, to better reflect strengths and weaknesses of our revised data. We are confident that this reviewer will now recognize that our work is suitable for publication in EMBO Reports as a Report type of contribution.

I do also have some comments about quality of the data and analysis:

1. The immunofluorescence and microscopy are not of a high quality. The microscopes described in methods look appropriate so I have concerns about the signal-to-noise in the experiments. This is important as the quantifications are key to interpretation of results (especially Fig 4).

We have invested a vast amount of time and effort to systematically improve the quality of the microscopy images throughout the manuscript relying on a laser scanning confocal microscope :

Fig. 1E
Fig. EV3A
Fig. EV3C
Fig. EV4A
Fig. EV5B
Appendix Fig. S6-9

2. The methods state that single kinetochore measurements were made on maximum intensity projections. I don't think this is appropriate as you are potentially merging kinetochores that are in the same x,y position but different z.

We based our fluorescence signal quantifications at individual kinetochores working on z-projections based on similar approaches performed by other leading laboratories in the field of kinetochore biology (e.g., Cleveland, Musacchio, Facchinetti, Sunkel). Rebuttal Figure 2 shows an example of our quantification strategy, in which we pay careful attention in placing our circular ROIs on the non-overlapping kinetochores only.

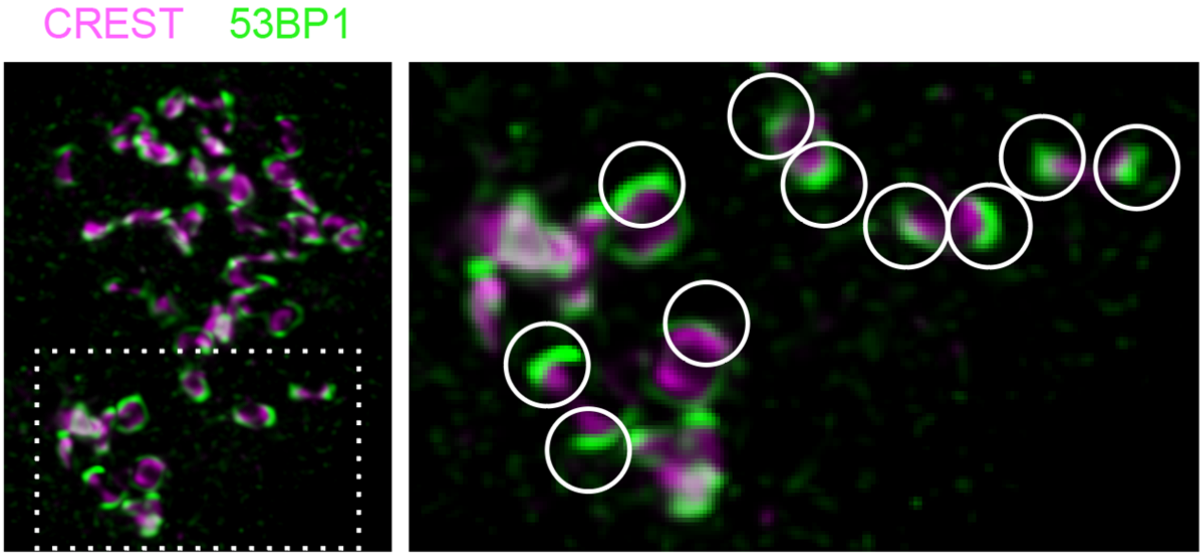

Rebuttal Figure 2. Representative image showing an immunofluorescence micrograph (z-projection) of an RPE1 cell stained against CREST and 53BP1. A blow-up of the region in the dotted square (left panel) is shown on the right, highlighting how KT ROIs (white circles) have been placed in all single KT quantifications performed in this manuscript.

3. There are no statistical analysis presented. This is not acceptable and makes it impossible to interpret the data.

We have now uniformly performed statistics for all figure panels in which image quantifications were performed. We have also compiled a table summarizing sample numerosity and the statistical tests performed (Table EV3).

References

Auckland P, Roscioli E, Coker HLE & McAinsh AD (2020) CENP-F stabilizes kinetochore-microtubule attachments and limits dynein stripping of corona cargoes. *The Journal of cell biology* **219:**

Cuella-Martin R, Oliveira C, Lockstone HE, Snellenberg S, Grolmusova N & Chapman JR (2016) 53BP1 Integrates DNA Repair and p53-Dependent Cell Fate Decisions via Distinct Mechanisms. *Molecular cell* **64:** 51–64

Fong CS, Mazo G, Das T, Goodman J, Kim M, O'Rourke BP, Izquierdo D & Tsou M-FB (2016) 53BP1 and USP28 mediate p53-dependent cell cycle arrest in response to centrosome loss and prolonged mitosis. *Elife* **5:** e16270

Jullien D, Vagnarelli P, Earnshaw WC & Adachi Y (2002) Kinetochore localisation of the DNA damage response component 53BP1 during mitosis. *Journal of cell science* **115:** 71–79

Lambrus BG & Holland AJ (2017) A New Mode of Mitotic Surveillance. *Trends in cell biology* **0:** 314–321

Lambrus BG, Daggubati V, Uetake Y, Scott PM, Clutario KM, Sluder G & Holland AJ (2016) A USP28-53BP1-p53-p21 signaling axis arrests growth after centrosome loss or prolonged mitosis. *The Journal of cell biology* **214:** 143–153

Marjanović M, Sánchez-Huertas C, Terré B, Gómez R, Scheel JF, Pacheco S, Knobel PA, Martínez-Marchal A, Aivio S, Palenzuela L, Wolfrum U, McKinnon PJ, Suja JA, Roig I, Costanzo V, Lüders J & Stracker TH (2015) CEP63 deficiency promotes p53-dependent microcephaly and reveals a role for the centrosome in meiotic recombination. *Nature communications* **6:** 7676–14

Meitinger F, Anzola JV, Kaulich M, Richardson A, Stender JD, Benner C, Glass CK, Dowdy SF, Desai A, Shiau AK & Oegema K (2016) 53BP1 and USP28 mediate p53 activation and G1 arrest after centrosome loss or extended mitotic duration. *The Journal of cell biology* **214:** 155–166

Meitinger F, Davis RL, Martinez MB, Shiau AK, Oegema K & Desai A (2022) Control of cell proliferation by memories of mitosis. *bioRxiv***:** 2022.11.14.515741

Phan TP, Maryniak AL, Boatwright CA, Lee J, Atkins A, Tijhuis A, Spierings DC, Bazzi H, Foijer F, Jordan PW, Stracker TH & Holland AJ (2021) Centrosome defects cause microcephaly by activating the 53BP1-USP28-TP53 mitotic surveillance pathway. *The EMBO journal* **40:** e106118

Santamaria A, Wang B, Elowe S, Malik R, Zhang F, Bauer M, Schmidt A, Sillje HH, Koerner R & Nigg EA (2010) The Plk1-dependent phosphoproteome of the early mitotic spindle. *Mol. Cell Proteomics*

Xiao C, Grzonka M, Meyer-Gerards C, Mack M, Figge R & Bazzi H (2021) Gradual centriole maturation associates with the mitotic surveillance pathway in mouse development. *EMBO reports* **22:** e51127

1st Revision - Editorial Decision 19th Sep 2023

Dear Prof. Fava,

Thank you for the submission of your revised manuscript to our editorial offices. I have now received the reports from three of the four referees that have been asked to re-evaluate your study, you will find below. Referee #4 was completely unresponsive to the invitations to re-assess the manuscript. However, going through your point-by-point-response I consider his/her points as adequately addressed. As you will see, the other referees now fully support the publication of the study in EMBO reports. Referees #2 and #3 have some remaining points and suggestions to improve the manuscript I ask you to address in a final revised manuscript. Please also provide a final p-b-p-responds concerning these points.

Moreover, I have these editorial requests:

- I would suggest this slightly modified title:
PLK1 promotes the mitotic surveillance pathway by controlling cytosolic 53BP1 availability

- Please remove the running title from the title page and reduce the number of keywords to five.

- We updated our journal's competing interests policy in January 2022 and request authors to consider both actual and perceived competing interests. Please review the policy https://www.embopress.org/competing-interests and update your competing interests if necessary. Please name this section 'Disclosure and Competing Interests Statement' and put it after the Acknowledgements section.

- Please remove the paragraph 'Material availability' form the manuscript. Then add a formal 'Data Availability' section (DAS) to the manuscript text file. This section should inform if large datasets have been submitted to a public database. If no datasets have been submitted, please just state there 'No large primary datasets have been generated and deposited'. I think the latter is the case. Finally, please also update the author checklist accordingly (change the entry regarding primary datasets to 'Not Applicable').

- Please order the manuscript sections like this, using these names:
Title page - Abstract - Keywords - Introduction - Results & Discussion - Materials and Methods - Data availability section (DAS) - Acknowledgements - Disclosure and Competing Interests Statement - References - Figure legends - Expanded View Figure legends

- Please format the reference according to our journal style (we need 'et al' for > 10 authors). See:
http://www.embopress.org/page/journal/14693178/authorguide#referencesformat

- Regarding data quantification and statistics, please make sure that the number "n" for how many independent experiments were performed, their nature (biological versus technical replicates), the bars and error bars (e.g. SEM, SD) and the test used to calculate p-values is indicated in the respective figure legends (main, EV and Appendix figures). Please also check that all the p-values are explained in the legend, and that these fit to those shown in the figure. Please provide statistical testing where applicable. Please avoid the phrase 'independent experiment', but clearly state if these were biological or technical replicates. Please also indicate (e.g. with n.s.) if testing was performed, but the differences are not significant. In case n=2 please show the data as separate datapoints or bars without error bars and statistics. See also:
http://www.embopress.org/page/journal/14693178/authorguide#statisticalanalysis

If n<5, please show single datapoints for diagrams.

- Tables EV1-EV3 are datasets. Please change their name to Dataset EV1-EV3 and make sure that the original excel files are uploaded with a title and a legend on the first TAB. Moreover, please change their callouts in the manuscript text file accordingly (Dataset EV1-EV3). Finally, please remove the EV table legends from the manuscript text file.

- Please upload the movie file ZIPed together with its corresponding legend, which can be provided as a README.txt file. Please add a callout to the movie to the manuscript text (Movie EV1). Finally, please remove the legend for the Movie from the manuscript text file.

- Please review the attached screenshot, which shows an enhanced version of panel E of Appendix Fig. S1. The images for V5 WT NT (first image first row) and V5 TP53BP1 KO NT (fifth image first row) look identical. Moreover WT NT does not fit to the corresponding DNA stain and the merge. Please check.

- Thanks for providing the source data. Please review the SD checklist again and make sure that all SD is actually provided. It seems that no SD is provided for previous panel 1H (where was this moved to - or has this been removed?) and 1G/S4A. Moreover, for 1C a panel 'TEV1' is mentioned. Please check.

- Finally, please find also attached a word file of the manuscript text (provided by our publisher) with changes we ask you to include in your final manuscript text and comments. Please use the attached file as basis for further revisions and provide your final manuscript file with track changes, in order that we can see any modifications done.

In addition, I would need from you:
- a short, two-sentence summary of the manuscript (not more than 35 words).
- two to four short bullet points highlighting the key findings of your study (two lines each).
- a schematic summary figure as separate file that provides a sketch of the major findings (not a data image) in jpeg or tiff format (with the exact width of 550 pixels and a height of not more than 400 pixels) that can be used as a visual synopsis on our website.

On a different note, I would like to alert you that EMBO Press offers a new format for a video-synopsis of work published with us, which essentially is a short, author-generated film explaining the core findings in hand drawings, and, as we believe, can be very useful to increase visibility of the work. Please see the following link for representative examples and their integration into the article web page:

https://www.embopress.org/video_synopses

https://www.embopress.org/doi/full/10.15252/embj.2019103932

Please let me know, should you be interested to engage in commissioning a similar video synopsis for your work. Operation instructions are available and intuitive.

I look forward to seeing the final revised version of your manuscript when it is ready. Please let me know if you have questions regarding the revision.

Best,

Achim Breiling
Senior Editor
EMBO Reports

Referee #1:

In this revised version of their paper the authors not only have addressed all of my criticisms to my satisfaction but also went beyond requested narrative revisions and included new mechanistic data regarding PLK1-mediated 53BP1/CENP-F kinetochore dynamics. I am now convinced that this work is relevant and important to the field and I am impressed by the quality of the new data. Therefore, I am happy to recommend publication in EMBO reports.

Referee #2:

In their revised manuscript, the authors have addressed the comments that were raised.
The authors have added a significant amount of data that strengthen their manuscript. The p53-53BP1 formation analysis makes this manuscript much stronger. I find that their data is solid and warrants publication, however, I find that at some points in the manuscript the conclusions and interpretations should be altered to make the conclusions better reflect the data:

1. Abstract: 'When 53BP1 abnormally persists at kinetochores, ..., MSP operativity blunted.'
These data refer to a situation of Plk1 inhibition. Since Plk1 has many targets, the blunted MSP cannot be causally linked solely to persistent 53BP1 KT localization as it is currently stated in the abstract. Only using a 53BP1 mutant that shows persistent kinetochore localization should justify this conclusion.
A fairer conclusion would be: 'Plk1 inhibition, which leads to persistent 53BP1 at kinetochores, results in a blunted MSP.'

2. Abstract: '...enabled us to show that measurement of mitotic timing by the MSP..' This is an overinterpretation of the data. There is no evidence presented of time measurement in mitosis.

3. The data that CENP-F is an (in)direct docking site for 53BP1 is very solid. The CENP-F mutation that abolishes 53BP1 loculation to kinetochores is also very clean and convincing. The fact that this mutation does not lead to a phenotype on its own is then turned around and interpreted as:
- The MSP must be a cytosolic process.

- The kinetochore is a 53BP1 sink, the precludes p53 activation in the cytosol to mediate an MSP response.
These last two aspects are deductions of the negative findings of the loss of phenotype of the CENP-F phenotype.
I realize that the BI2536 Plk1i treatment can induce a p53-53BP1-PLA signal in this mutant, but we don't know which other proteins are differentially recruited to the CENP-F mutant, so this phenotype is not causally linked to 53BP1.
It would be much better, in my opinion, to reflect on this in the discussion section, but not present it in the abstract, because there is no direct evidence on the role of cytoplasmic 53BP1 in activating p53.

4. Figure 2: senescence is now replaced by p21-dependent cell cycle arrest. Also this terminology is problematic, because it is not shown to be p21-dependent. In line 198, the authors state: 'we monitored the occurrence of p21-dependent cell cycle arrest in relation to the time spent in mitosis'
This should be changed into: 'the occurrence of p21 expression'

5. Concerning my previous comment, I feel that the usage of MSP/mitotic surveillance pathway is confusing and inflated. I realize that authors debate this vision, but only few labs use this term and it is highly confusing with the spindle assembly checkpoint. In my perception, it reflects the ability of cells to activate p53 in situation of prolonged spindle checkpoint activation. It would be much clearer to readers if the pathway is referred to as p53 activation during prolonged mitosis.

Referee #3:

Review of the revised EMBO Reports manuscript 57234V2 entitled "PLK1 promotes the mitotic surveillance pathway by controlling 53BP1 cytosolic availability" by Luca L. Fava and coworkers.

I was quite fond of this study already in its previous version. In their revised manuscript, Fava and coworkers have not only satisfactorily addressed the few points raised by me but further improved their work by additional experimental data. I am especially excited about the PLA-assay, which further strengthens the authors' previous conclusion that Plk1-dependent dissociation of 53BP1 from kinetochores is required for subsequent 53BP1-p53 interaction.

I have also carefully read the other reviews and do not share the fundamental concerns raised by referee # 2. Instead, I agree with the argumentation with which Fava et al. meet this criticism.

In summary, this has become an even more impressive body of work during its revision, which is now truely fit for publication in EMBO Reports.

Referee # 2 writes: "...since PLK1 has many roles, it cannot be concluded that the observed phenotype is related to PLK1-mediated 53bp1 dynamics at kinetochores." This scepticism is largely shared by referee # 4. Below, I am suggesting two relatively easy experiments, which would meet this criticism. However, conducting these should NOT be requirement for publication, because the fact that the CENP-FE564P variant renders Plk1 dispensable is already a very strong argument against a 53BP1-independent role of Plk1 for the MSP.
1) Following pull-down of V5-tagged 53BP1 from early mitotic cells, one could add ATP/Mg2+ and recombinant Plk1 (although the 53BP1-bound Plk1 might suffice) to the beads and ask whether this results in elution of CENP-F (but not 53BP1). A sample, which is additionally supplemented with BI-2536 would serve as a control, here, to ensure that one is looking at an effect of Plk1-specific phosphorylation.
2) Mutation of 13 putative Plk1 sites did not suffice to render 53BP1's kinetochore association resistant to Plk1. While this argues that 53BP1 might not be the relevant Plk1 target, docking of Plk1 to 53BP1 might still be required (e.g. for CENP-F phosphorylation). Therefore, it might be worth while to assay the Plk1-binding deficient 53BP1 variant described by Yaffe and colleagues (van Vugt et al., 2010). Might this 53BP1-S376A be resistant to Plk1-dependent eviction from kinetochores?

Point-by-point response to Reviewers' comments (our responses are in blue font)

Referee #1:

In this revised version of their paper the authors not only have addressed all of my criticisms to my satisfaction but also went beyond requested narrative revisions and included new mechanistic data regarding PLK1-mediated 53BP1/CENP-F kinetochore dynamics. I am now convinced that this work is relevant and important to the field and I am impressed by the quality of the new data. Therefore, I am happy to recommend publication in EMBO reports.

Many thanks for recognizing the effectiveness of our effort in improving the manuscript.

Referee #2:

In their revised manuscript, the authors have addressed the comments that were raised. The authors have added a significant amount of data that strengthen their manuscript. The p53-53BP1 formation analysis makes this manuscript much stronger. I find that their data is solid and warrants publication,

We appreciate very much that this referee is now supporting publication of our work.

however, I find that at some points in the manuscript the conclusions and interpretations should be altered to make the conclusions better reflect the data:

1. Abstract: 'When 53BP1 abnormally persists at kinetochores, ..., MSP operativity blunted.'
These data refer to a situation of Plk1 inhibition. Since Plk1 has many targets, the blunted MSP cannot be causally linked solely to persistent 53BP1 KT localization as it is currently stated in the abstract. Only using a 53BP1 mutant that shows persistent kinetochore localization should justify this conclusion.
A fairer conclusion would be: 'Plk1 inhibition, which leads to persistent 53BP1 at kinetochores, results in a blunted MSP.'

Ok, we have modified the central part of our abstract to eliminate the interpretation layer, now referring exclusively to our data, as requested.

2. Abstract: '...enabled us to show that measurement of mitotic timing by the MSP..' This is an overinterpretation of the data. There is no evidence presented of time measurement in mitosis.

We disagree with this statement, as the assays presented in Figs 2F and Appendix Fig. S4 dealt precisely with this aspect, namely how the mitotic timing reflects into p21 accumulation. P21 is on the other hand the main cell cycle arrest effector of the MSP. Thus, we maintain the wording of our abstract concerning this aspect.

3. The data that CENP-F is an (in)direct docking site for 53BP1 is very solid. The CENP-F mutation that abolishes 53BP1 loculation to kinetochores is also very clean and convincing. The fact that this mutation does not lead to a phenotype on its own is then turned around and interpreted as:
- The MSP must be a cytosolic process.
- The kinetochore is a 53BP1 sink, the precludes p53 activation in the cytosol to mediate an MSP response. These last two aspects are deductions of the negative findings of the loss of phenotype of the CENP-F phenotype.

I realize that the BI2536 Plk1i treatment can induce a p53-53BP1-PLA signal in this mutant, but we don't know which other proteins are differentially recruited to the CENP-F mutant, so this phenotype is not causally linked to 53BP1. It would be much better, in my opinion, to reflect on this in the discussion section, but not present it in the abstract, because there is no direct evidence on the role of cytoplasmic 53BP1 in activating p53.

To meet this reviewer's criticism, we now state in the abstract that the MSP is NOT a kinetochore process, rather than stating that it is a cytosolic process. In the following sentence, introduced by 'we propose', we introduce the interpretation layer instead.

4. Figure 2: senescence is now replaced by p21-dependent cell cycle arrest. Also this terminology is problematic, because it is not shown to be p21-dependent. In line 198, the authors state: 'we monitored the occurrence of p21-dependent cell cycle arrest in relation to the time spent in mitosis'
This should be changed into: 'the occurrence of p21 expression'

Ok, we have changed our text accordingly.

5. Concerning my previous comment, I feel that the usage of MSP/mitotic surveillance pathway is confusing and inflated. I realize that authors debate this vision, but only few labs use this term and it is highly confusing with the spindle assembly checkpoint. In my perception, it reflects the ability of cells to activate p53 in situation of prolonged spindle checkpoint activation. It would be much clearer to readers if the pathway is referred to as p53 activation during prolonged mitosis.

We are not concerned about this issue as 'MSP' can be defined in less than two lines as we do in our abstract: '*a sensor that monitors the duration of cell division, promoting p53-dependent cell cycle arrest when a critical time threshold is surpassed*'. SAC and MSP are distinct pathways, monitoring different cues, relying on different signal transduction cascades / effectors, and eliciting distinct cell cycle consequences. We are confident that EMBO Reports readers will grasp the difference while going through the abstract of our work already, irrespectively of liking the chosen terminology or not.
We note that one emerging study by Meitinger and colleagues (presently as preprint), introduce a novel and different terminology to describe the exact same pathway, namely the "Mitotic Stopwatch Pathway". Given such circumstances, we introduced also "Mitotic stopwatch" among the keywords of the paper and the alternative name in the abstract. Luckily, 'MSP' can serve as acronym for both names.

Referee #3:

Review of the revised EMBO Reports manuscript 57234V2 entitled "PLK1 promotes the mitotic surveillance pathway by controlling 53BP1 cytosolic availability" by Luca L. Fava and coworkers.

I was quite fond of this study already in its previous version. In their revised manuscript, Fava and coworkers have not only satisfactorily addressed the few points raised by me but further improved their work by additional experimental data. I am especially excited about the PLA-assay, which further strengthens the authors' previous conclusion that Plk1-dependent dissociation of 53BP1 from kinetochores is required for subsequent 53BP1-p53 interaction.

Many thanks, we have been also very pleased to see how well the PLA data aligned to the rest of the story.

I have also carefully read the other reviews and do not share the fundamental concerns raised by referee # 2. Instead, I agree with the argumentation with which Fava et al. meet this criticism.

In summary, this has become an even more impressive body of work during its revision, which is now truely fit for publication in EMBO Reports.

Referee # 2 writes: "...since PLK1 has many roles, it cannot be concluded that the observed phenotype is related to PLK1-mediated 53bp1 dynamics at kinetochores." This scepticism is largely shared by referee # 4. Below, I am suggesting two relatively easy experiments, which would meet this criticism. However, conducting these should NOT be requirement for publication, because the fact that the CENP-FE564P variant renders Plk1 dispensable is already a very strong argument against a 53BP1-independent role of Plk1 for the MSP.
1) Following pull-down of V5-tagged 53BP1 from early mitotic cells, one could add ATP/Mg2+ and recombinant Plk1 (although the 53BP1-bound Plk1 might suffice) to the beads and ask whether this results in elution of CENP-F (but not 53BP1). A sample, which is additionally supplemented with BI-2536 would serve as a control, here, to ensure that one is looking at an effect of Plk1-specific phosphorylation.

Many thanks for this suggestion. This 'ex-cellulo' approach might also provide the basis for the convenient identification of the phospho-sites accounting for the phenomenon by means of mass spectrometry. Both endeavours are clearly beyond the scope of this manuscript, as stated by this reviewer.

2) Mutation of 13 putative Plk1 sites did not suffice to render 53BP1's kinetochore association resistant to Plk1. While this argues that 53BP1 might not be the relevant Plk1 target, docking of Plk1 to 53BP1 might still be required (e.g. for CENP-F phosphorylation). Therefore, it might be worth while to assay the Plk1-binding deficient 53BP1 variant described by Yaffe and colleagues (van Vugt et al., 2010). Might this 53BP1-S376A be resistant to Plk1-dependent eviction from kinetochores?

We have not formally excluded that PLK1 docking to 53BP1 is required to phosphorylate other substrates, e.g., CENP-F. As our discussion paragraph deals exclusively with the identity of the PLK1 substrate accounting for the reported phenomena, we prefer avoiding alluding to the possibility of 53BP1 representing the PLK1 docking site, for the seek of simplicity.

Prof. Luca Fava
University of Trento
Department of Cellular, Computational and Integrative Biology - CIBIO
Via Sommarive 9
Trento, Trento 38123 Povo
Italy

Dear Prof. Fava,

I am very pleased to accept your manuscript for publication in the next available issue of EMBO reports. Thank you for your contribution to our journal.

At the end of this email I include important information about how to proceed. Please ensure that you take the time to read the information and complete and return the necessary forms to allow us to publish your manuscript as quickly as possible.

As part of the EMBO publication's Transparent Editorial Process, EMBO reports publishes online a Review Process File to accompany accepted manuscripts. As you are aware, this File will be published in conjunction with your paper and will include the referee reports, your point-by-point response and all pertinent correspondence relating to the manuscript.

If you do NOT want this File to be published, please inform the editorial office within 2 days, if you have not done so already, otherwise the File will be published by default [contact: emboreports@embo.org]. If you do opt out, the Review Process File link will point to the following statement: "No Review Process File is available with this article, as the authors have chosen not to make the review process public in this case."

Should you be planning a Press Release on your article, please get in contact with emboreports@wiley.com as early as possible, in order to coordinate publication and release dates.

Thank you again for your contribution to EMBO reports and congratulations on a successful publication. Please consider us again in the future for your most exciting work.

Yours sincerely,

Achim Breiling
Editor
EMBO Reports

****

THINGS TO DO NOW:

Please note that you will be contacted by Wiley Author Services to complete licensing and payment information. The required 'Page Charges Authorization Form' is available here: https://www.embopress.org/pb-assets/embo-site/er_apc.pdf - please download and complete the form and return to embopressproduction@wiley.com

EMBO Press participates in many Publish and Read agreements that allow authors to publish Open Access with reduced/no publication charges. Check your eligibility: https://authorservices.wiley.com/author-resources/Journal-Authors/open-access/affiliation-policies-payments/index.html

You will receive proofs by e-mail approximately 2-3 weeks after all relevant files have been sent to our Production Office; you should return your corrections within 2 days of receiving the proofs.

Please inform us if there is likely to be any difficulty in reaching you at the above address at that time. Failure to meet our deadlines may result in a delay of publication, or publication without your corrections.

All further communications concerning your paper should quote reference number EMBOR-2023-57234V3 and be addressed to emboreports@wiley.com.

Should you be planning a Press Release on your article, please get in contact with emboreports@wiley.com as early as possible, in order to coordinate publication and release dates.

EMBO Press Author Checklist

Corresponding Author Name: Luca Fava	
Journal Submitted to: EMBO Reports	
Manuscript Number: EMBOR-2023-57234V2	

USEFUL LINKS FOR COMPLETING THIS FORM
[The EMBO Journal - Author Guidelines]
[EMBO Reports - Author Guidelines]
[Molecular Systems Biology - Author Guidelines]
[EMBO Molecular Medicine - Author Guidelines]

Reporting Checklist for Life Science Articles (updated January

This checklist is adapted from Materials Design Analysis Reporting (MDAR) Checklist for Authors. MDAR establishes a minimum set of requirements in transparent reporting in the life sciences (see Statement of Task: 10.31222/osf.io/9sm4x). Please follow the journal's guidelines in preparing your

Please note that a copy of this checklist will be published alongside your article.

Abridged guidelines for figures

1. Data

The data shown in figures should satisfy the following conditions:

- the data were obtained and processed according to the field's best practice and are presented to reflect the results of the experiments in an accurate and unbiased manner.
- ideally, figure panels should include only measurements that are directly comparable to each other and obtained with the same assay.
- plots include clearly labeled error bars for independent experiments and sample sizes. Unless justified, error bars should not be shown for technical
- if n<5, the individual data points from each experiment should be plotted. Any statistical test employed should be justified.
- Source Data should be included to report the data underlying figures according to the guidelines set out in the authorship guidelines on Data

2. Captions

Each figure caption should contain the following information, for each panel where they are relevant:

- a specification of the experimental system investigated (eg cell line, species name).
- the assay(s) and method(s) used to carry out the reported observations and measurements.
- an explicit mention of the biological and chemical entity(ies) that are being measured.
- an explicit mention of the biological and chemical entity(ies) that are altered/varied/perturbed in a controlled manner.
- the exact sample size (n) for each experimental group/condition, given as a number, not a range;
- a description of the sample collection allowing the reader to understand whether the samples represent technical or biological replicates (including how many animals, litters, cultures, etc.).
- a statement of how many times the experiment shown was independently replicated in the laboratory.
- definitions of statistical methods and measures:
  - common tests, such as t-test (please specify whether paired vs. unpaired), simple χ2 tests, Wilcoxon and Mann-Whitney tests, can be unambiguously identified by name only, but more complex techniques should be described in the methods section;
  - are tests one-sided or two-sided?
  - are there adjustments for multiple comparisons?
  - exact statistical test results, e.g., P values = x but not P values < x;
  - definition of 'center values' as median or average;
  - definition of error bars as s.d. or s.e.m.

Please complete ALL of the questions below.
Select "Not Applicable" only when the requested information is not relevant for your study.

Materials

Newly Created Materials	Information included in the manuscript?	In which section is the information available? (Reagents and Tools Table, Materials and Methods, Figures, Data Availability Section)
New materials and reagents need to be available; do any restrictions apply?	Yes	Materials and Methods

Antibodies	Information included in the manuscript?	In which section is the information available? (Reagents and Tools Table, Materials and Methods, Figures, Data Availability Section)
For **antibodies** provide the following information:<br>- Commercial antibodies: RRID (if possible) or supplier name, catalogue number and or/clone number<br>- Non-commercial: RRID or citation	Yes	Materials and Methods

DNA and RNA sequences	Information included in the manuscript?	In which section is the information available? (Reagents and Tools Table, Materials and Methods, Figures, Data Availability Section)
**Short novel DNA or RNA including primers, probes**: provide the sequences.	Yes	Table EV2

Cell materials	Information included in the manuscript?	In which section is the information available? (Reagents and Tools Table, Materials and Methods, Figures, Data Availability Section)
**Cell lines:** Provide species information, strain. Provide accession number in repository **OR** supplier name, catalog number, clone number, and/**OR** RRID.	Yes	Materials and Methods
**Primary cultures:** Provide species, strain, sex of origin, genetic modification status.	Not Applicable	
Report if the cell lines were recently **authenticated** (e.g., by STR profiling) and tested for mycoplasma contamination.	Yes	Materials and Methods

Experimental animals	Information included in the manuscript?	In which section is the information available? (Reagents and Tools Table, Materials and Methods, Figures, Data Availability Section)
**Laboratory animals or Model organisms:** Provide species, strain, sex, age, genetic modification status. Provide accession number in repository **OR** supplier name, catalog number, clone number, **OR** RRID.	Not Applicable	
**Animal observed in or captured from the field:** Provide species, sex, and age where possible.	Not Applicable	
Please detail **housing and husbandry conditions**.	Not Applicable	

Plants and microbes	Information included in the manuscript?	In which section is the information available? (Reagents and Tools Table, Materials and Methods, Figures, Data Availability Section)
**Plants:** provide species and strain, ecotype and cultivar where relevant, unique accession number if available, and source (including location for collected wild specimens).	Not Applicable	
**Microbes:** provide species and strain, unique accession number if available, and source.	Not Applicable	

Human research participants	Information included in the manuscript?	In which section is the information available? (Reagents and Tools Table, Materials and Methods, Figures, Data Availability Section)
If collected and within the bounds of privacy constraints report on age, sex and gender or ethnicity for all study participants.	Not Applicable	

Core facilities	Information included in the manuscript?	In which section is the information available? (Reagents and Tools Table, Materials and Methods, Figures, Data Availability Section)
If your work benefited from core facilities, was their service mentioned in the acknowledgments section?	Yes	Acknowledgements

Design

Study protocol	Information included in the manuscript?	In which section is the information available? (Reagents and Tools Table, Materials and Methods, Figures, Data Availability Section)

If study protocol has been **pre-registered, provide DOI in the manuscript**. For clinical trials, provide the trial registration number **OR** cite DOI.	Not Applicable	
Report the **clinical trial registration number** (at ClinicalTrials.gov or equivalent), where applicable.	Not Applicable	

Laboratory protocol	Information included in the manuscript?	In which section is the information available? (Reagents and Tools Table, Materials and Methods, Figures, Data Availability Section)
Provide DOI OR other citation details if **external detailed step-by-step protocols** are available.	Yes	Materials and Methods

Experimental study design and statistics	Information included in the manuscript?	In which section is the information available? (Reagents and Tools Table, Materials and Methods, Figures, Data Availability Section)
Include a statement about **sample size** estimate even if no statistical methods were used.	Yes	Table EV3
Were any steps taken to minimize the effects of subjective bias when allocating animals/samples to treatment (e.g. **randomization procedure**)? If yes, have they been described?	Not Applicable	
Include a statement about **blinding** even if no blinding was done.	Not Applicable	
Describe **inclusion/exclusion criteria** if samples or animals were excluded from the analysis. Were the criteria pre-established?<br><br>If sample or data points were omitted from analysis, report if this was due to attrition or intentional exclusion and provide justification.	Not Applicable	
For every figure, are **statistical tests** justified as appropriate? Do the data meet the assumptions of the tests (e.g., normal distribution)? Describe any methods used to assess it. Is there an estimate of variation within each group of data? Is the variance similar between the groups that are being statistically compared?	Yes	Materials and Methods and Table EV3

Sample definition and in-laboratory replication	Information included in the manuscript?	In which section is the information available? (Reagents and Tools Table, Materials and Methods, Figures, Data Availability Section)
In the figure legends: state number of times the experiment was **replicated** in laboratory.	Yes	Figures
In the figure legends: define whether data describe **technical or biological replicates**.	Yes	Figures

Ethics

Ethics	Information included in the manuscript?	In which section is the information available? (Reagents and Tools Table, Materials and Methods, Figures, Data Availability Section)
Studies involving **human participants**: State details of **authority granting ethics approval** (IRB or equivalent committee(s), provide reference number for approval.	Not Applicable	
Studies involving **human participants**: Include a statement confirming that **informed consent** was obtained from all subjects and that the experiments conformed to the principles set out in the WMA Declaration of Helsinki and the Department of Health and Human Services Belmont Report.	Not Applicable	
Studies involving **human participants:** For publication of **patient photos**, include a statement confirming that consent to publish was obtained.	Not Applicable	
Studies involving experimental **animals**: State details of **authority granting ethics approval** (IRB or equivalent committee(s), provide reference number for approval. Include a statement of compliance with ethical regulations.	Not Applicable	
Studies involving **specimen and field samples:** State if relevant **permits** obtained, provide details of authority approving study; if none were required, explain why.	Not Applicable	

Dual Use Research of Concern (DURC)	Information included in the manuscript?	In which section is the information available? (Reagents and Tools Table, Materials and Methods, Figures, Data Availability Section)
Could your study fall under dual use research restrictions? Please check biosecurity documents and list of **select agents and toxins** (CDC): https://www.selectagents.gov/sat/list.htm	Not Applicable	
If you used a select agent, is the security level of the lab appropriate and reported in the manuscript?	Not Applicable	
If a study is subject to dual use research of concern regulations, is the name of the **authority granting approval and reference number** for the regulatory approval provided in the manuscript?	Not Applicable	

Reporting

The MDAR framework recommends adoption of discipline-specific guidelines, established and endorsed through community initiatives. Journals have their own policy about requiring specific guidelines and recommendations to complement MDAR.

Adherence to community standards	Information included in the manuscript?	In which section is the information available? (Reagents and Tools Table, Materials and Methods, Figures, Data Availability Section)
State if relevant guidelines or checklists (e.g., **ICMJE, MIBBI, ARRIVE, PRISMA**) have been followed or provided.	Not Applicable	
For **tumor marker prognostic studies,** we recommend that you follow the **REMARK** reporting guidelines (see link list at top right). See author guidelines, under 'Reporting Guidelines'. Please confirm you have followed these guidelines.	Not Applicable	
For **phase II and III randomized controlled trials**, please refer to the **CONSORT** flow diagram (see link list at top right) and submit the CONSORT checklist (see link list at top right) with your submission. See author guidelines, under 'Reporting Guidelines'. Please confirm you have submitted this list.	Not Applicable	

Data Availability

Data availability	Information included in the manuscript?	In which section is the information available? (Reagents and Tools Table, Materials and Methods, Figures, Data Availability Section)
Have **primary datasets** been deposited according to the journal's guidelines (see 'Data Deposition' section) and the respective accession numbers provided in the Data Availability Section?	Not Applicable	
Were **human clinical and genomic datasets** deposited in a public access-controlled repository in accordance to ethical obligations to the patients and to the applicable consent agreement?	Not Applicable	
Are **computational models** that are central and integral to a study available without restrictions in a machine-readable form? Were the relevant accession numbers or links provided?	Not Applicable	
If publicly available data were reused, provide the respective **data citations in the reference list**.	Not Applicable	
