# Supplementary material for: PLK1 promotes the mitotic surveillance pathway by controlling cytosolic 53BP1 availability
Source: EMBO Rep. 2023 Oct 27;24(12):e57234. doi: 10.15252/embr.202357234 (PMC10702821; doi:10.15252/embr.202357234)
Supplement: Supplementary file 8 — Source Data for Expanded View [file EMBR-24-e57234-s004.zip › Figure EV1.zip!/Figure EV1/EV1D/Source data EV1D.pdf]

Western blot membranes relative to Fig EV1D

CENP-F

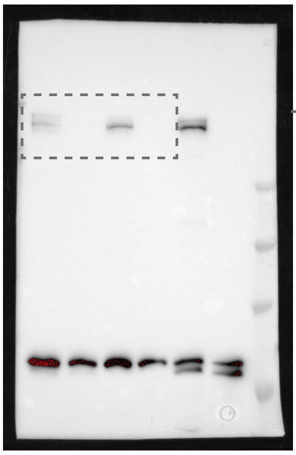

460

53BP1

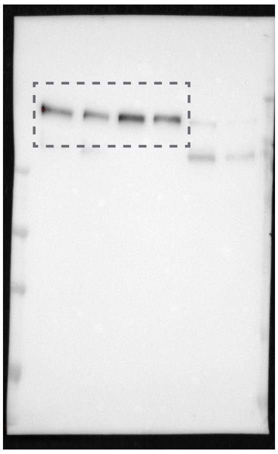

460

60

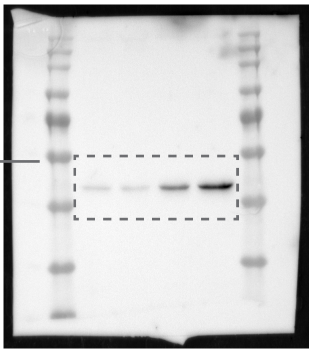

cyclin A

140

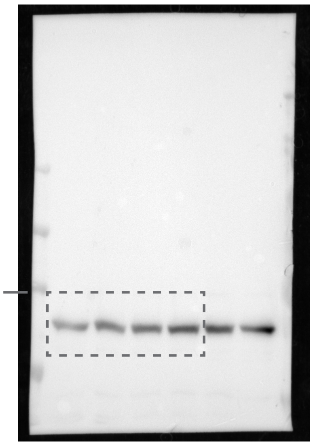

vinculin
